# Supplementary material for: Quantitative Modeling of TLR Signaling Reveals Missing Negative Feedback Guiding Identification of TANK-IKKε Checkpoint
Source: bioRxiv. 2026 Aug 16:2026.08.03.742528. Preprint. [Version 2] doi: 10.64898/2026.08.03.742528 (PMC13482443; doi:10.64898/2026.08.03.742528)

Data-Driven Modeling of the Mouse Macrophage Toll-like Receptor Signaling Pathway

Manes et al, 2026
Supplemental Material

Figure S6. All of the experimental data (normalized fold change ratios) used to train the model are plotted (blue bars; height = 0.02). The corresponding simulation results from the top 20 ranked models are also plotted (red lines). The vertical magenta line indicates the time at which LPS was added (t = 600 s).

Experimental Measurement (*FC Ratio*)

Simulation Results (top 20 ranked models)

LPS Added (t = 600 s)

TLR4

Experiment vs. Simulation: 30 nM LPS, Cell Surface / Total TLR4

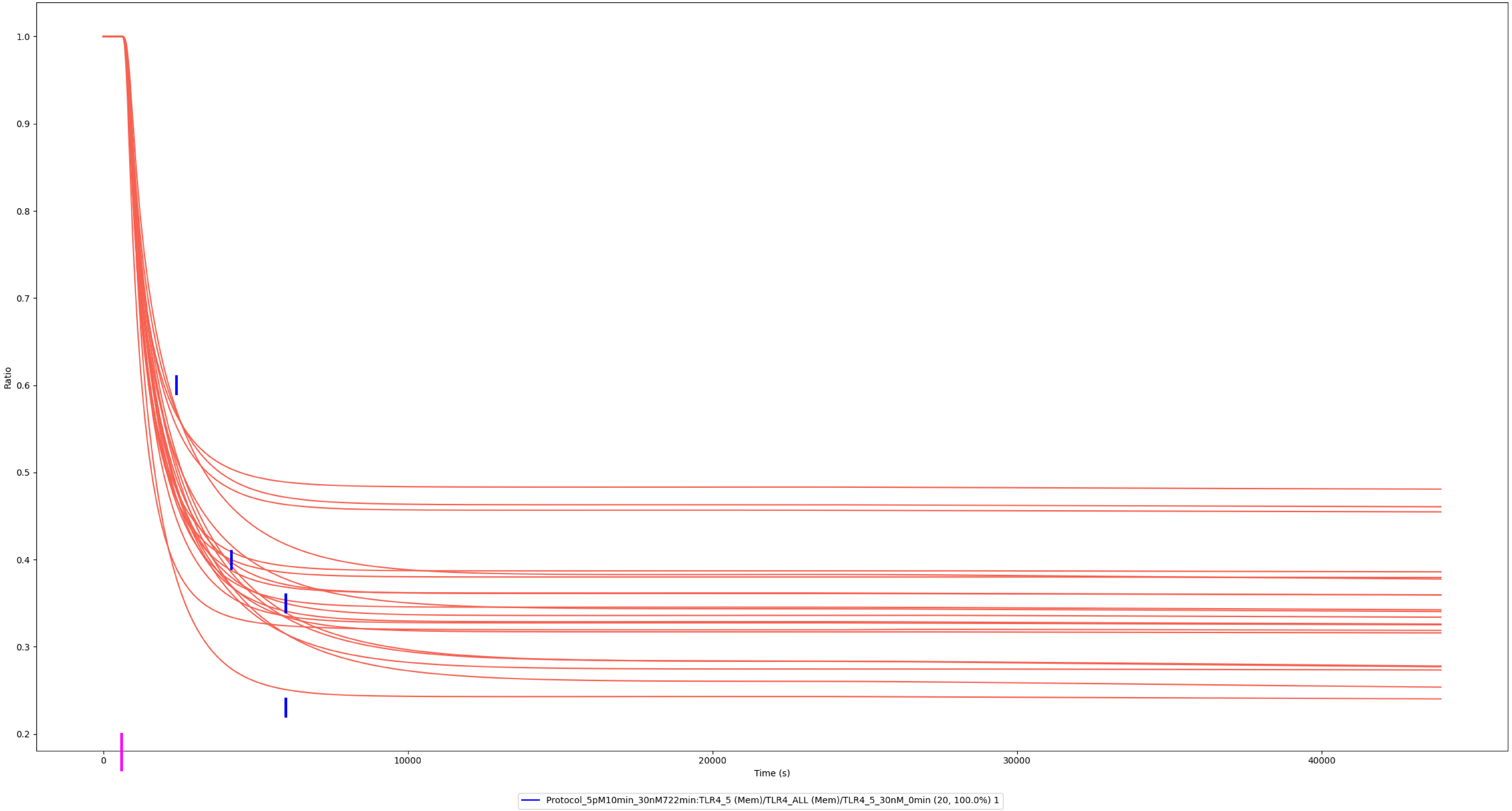

Experiment vs. Simulation: 0.03 nM LPS, Cell Surface / Total TLR4

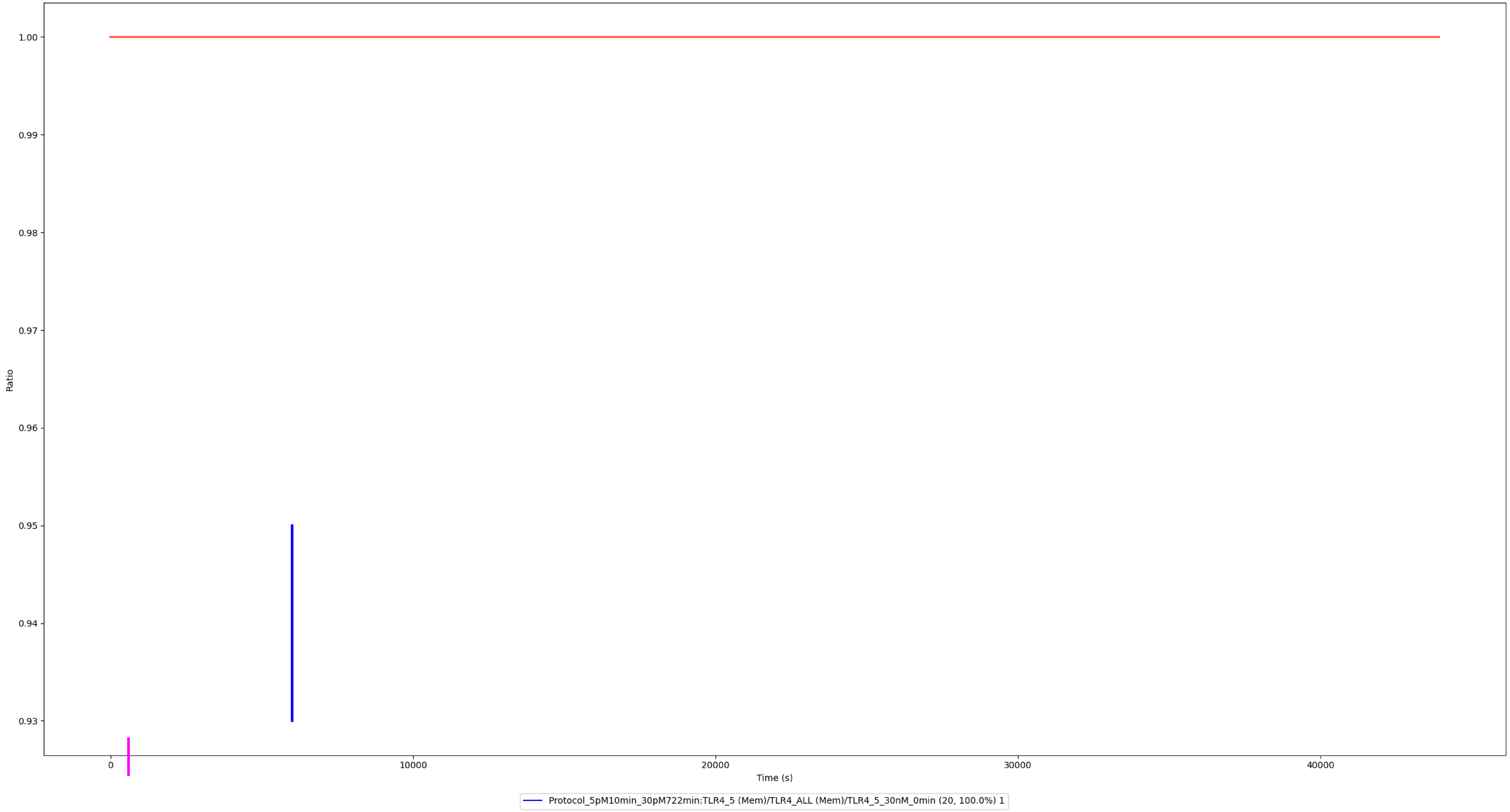

Experiment vs. Simulation: 0.3 nM LPS, Cell Surface / Total TLR4

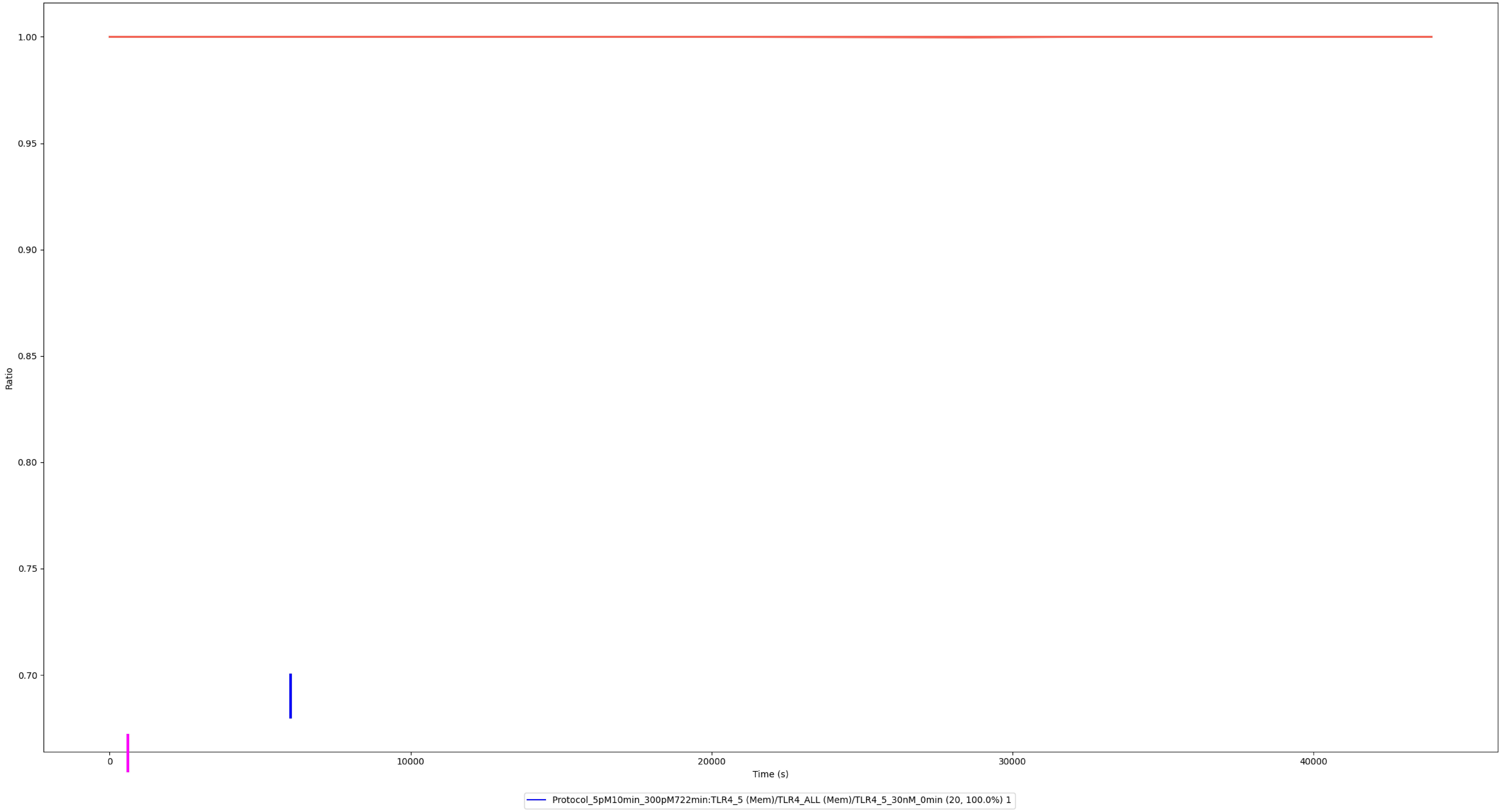

Experiment vs. Simulation: 3 nM LPS, Cell Surface / Total TLR4

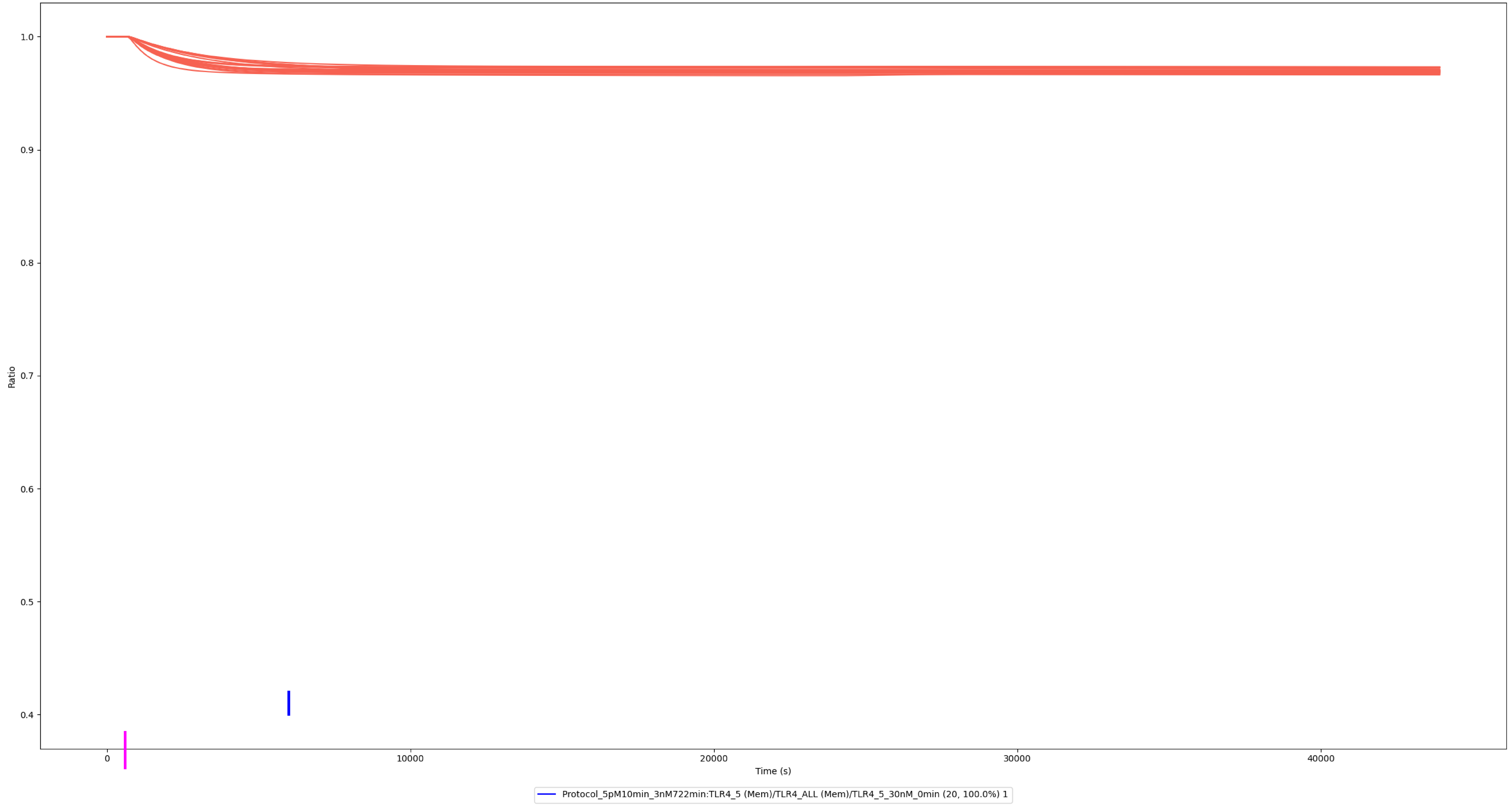

Experiment vs. Simulation: 300 nM LPS, Cell Surface / Total TLR4

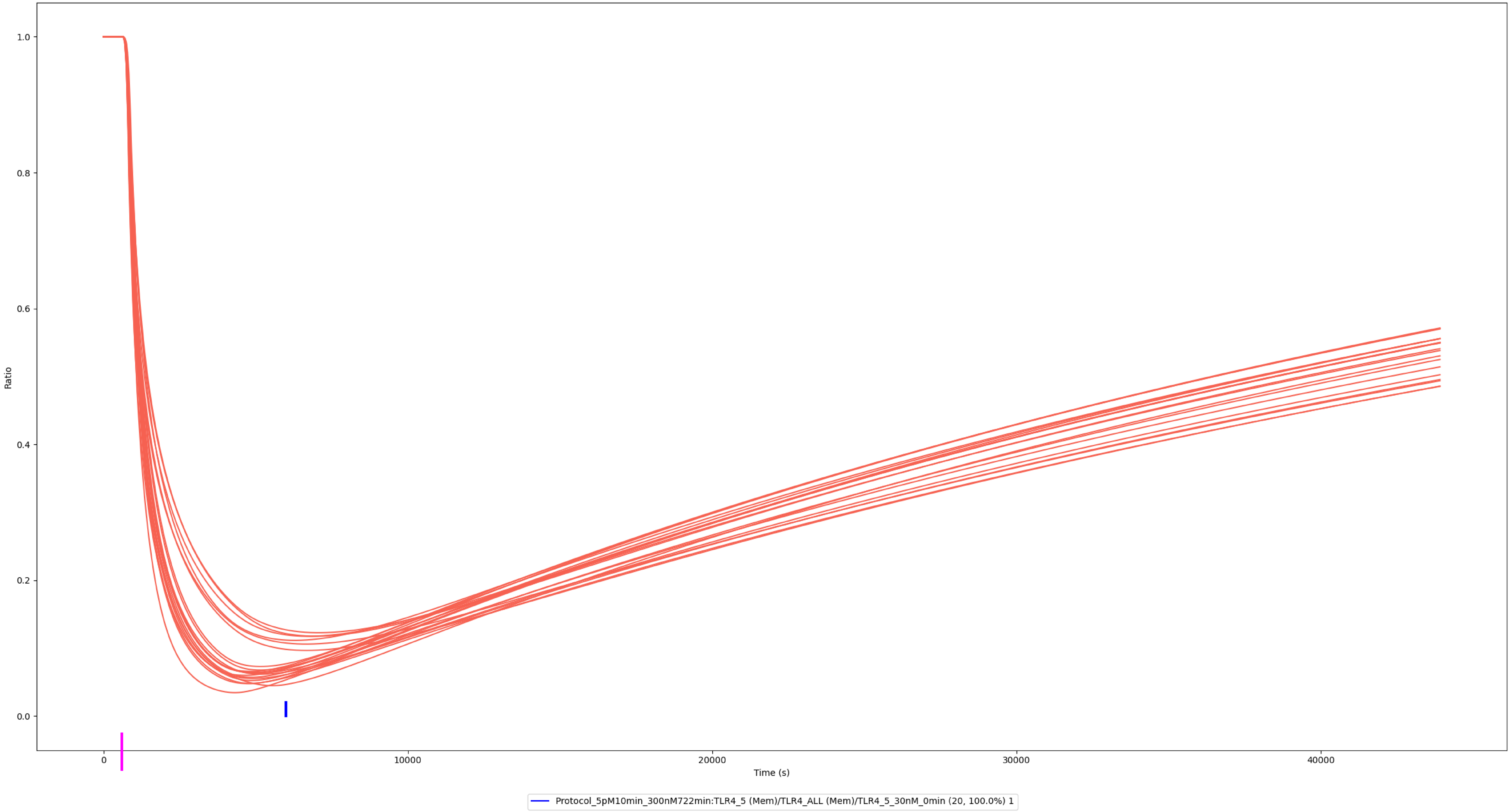

MyD88

Experiment vs. Simulation: 30 nM LPS, MAL:MyD88

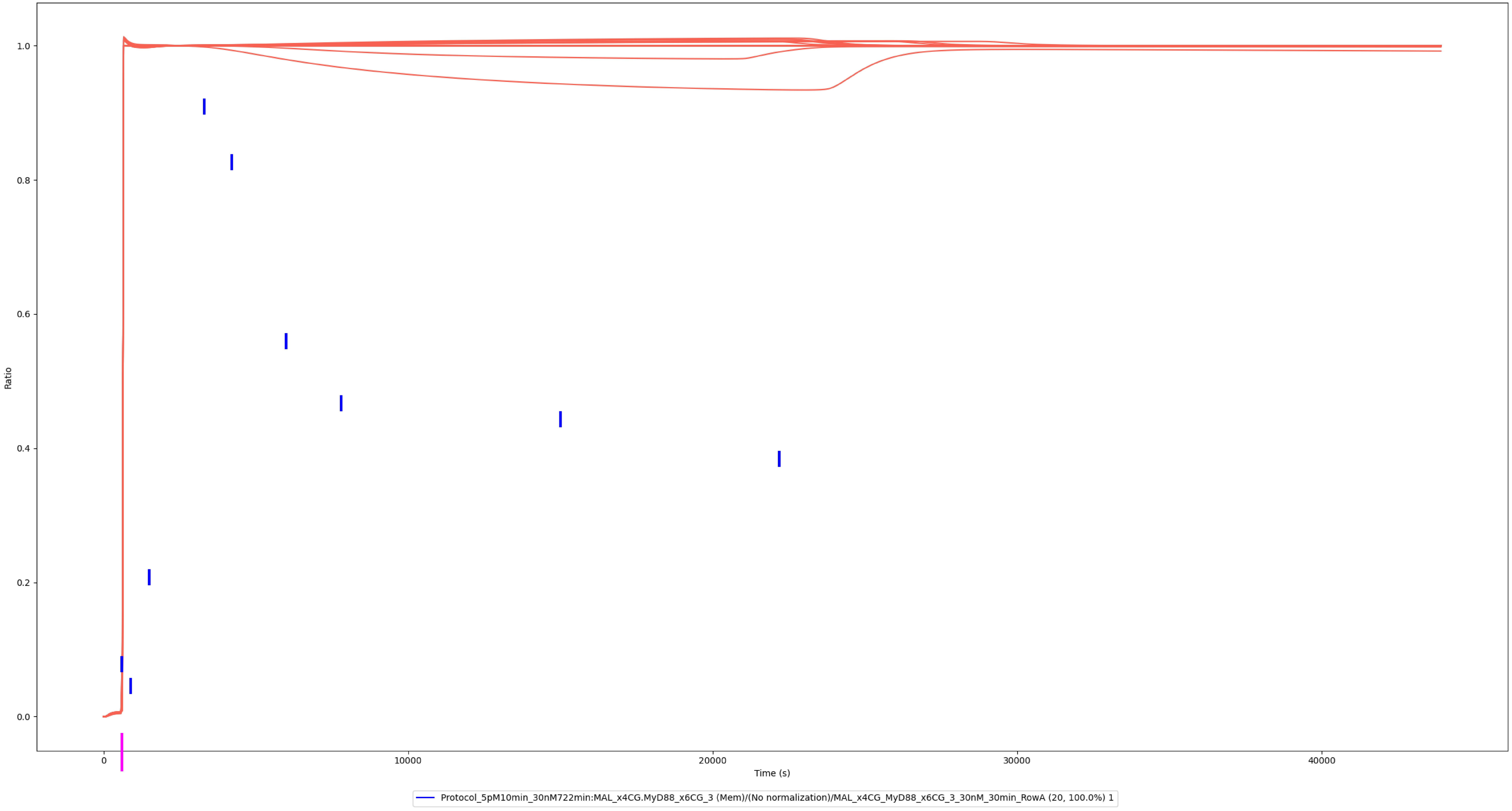

Experiment vs. Simulation: 30 nM LPS, MAL:MyD88

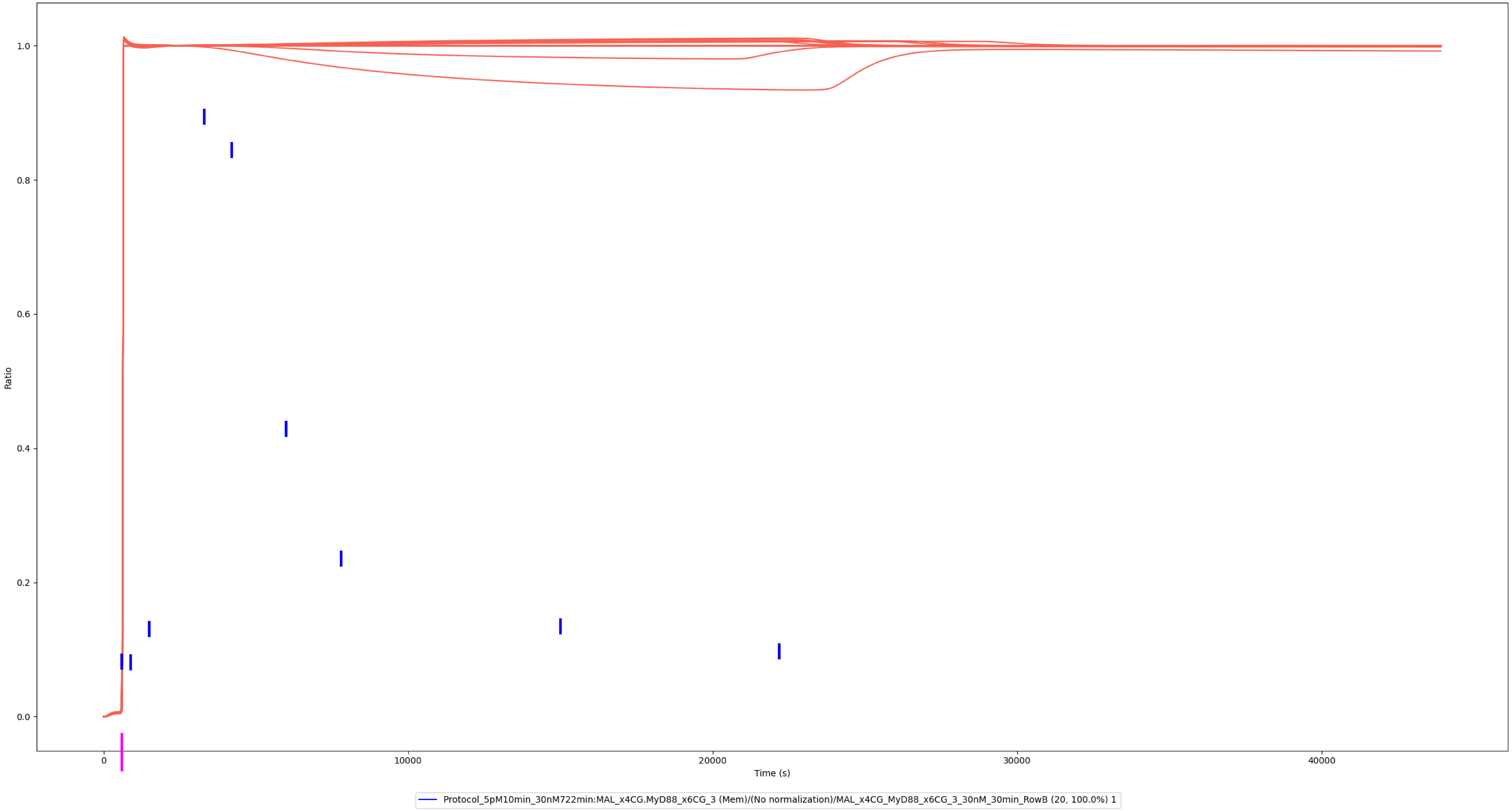

IRAK4

Experiment vs. Simulation: 30 nM LPS, MyD88:IRAK4

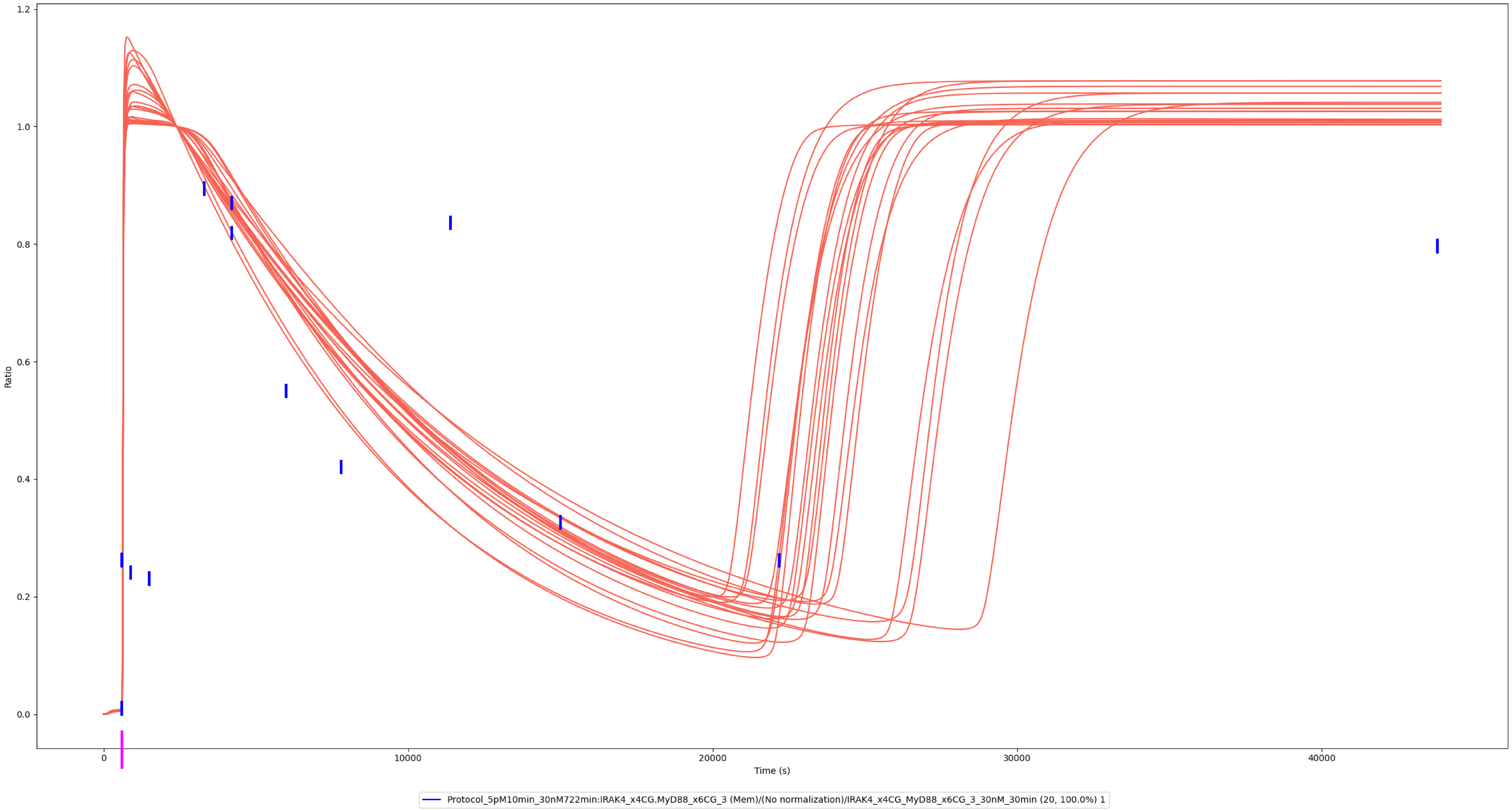

Experiment vs. Simulation: 30 nM LPS, MyD88:IRAK4

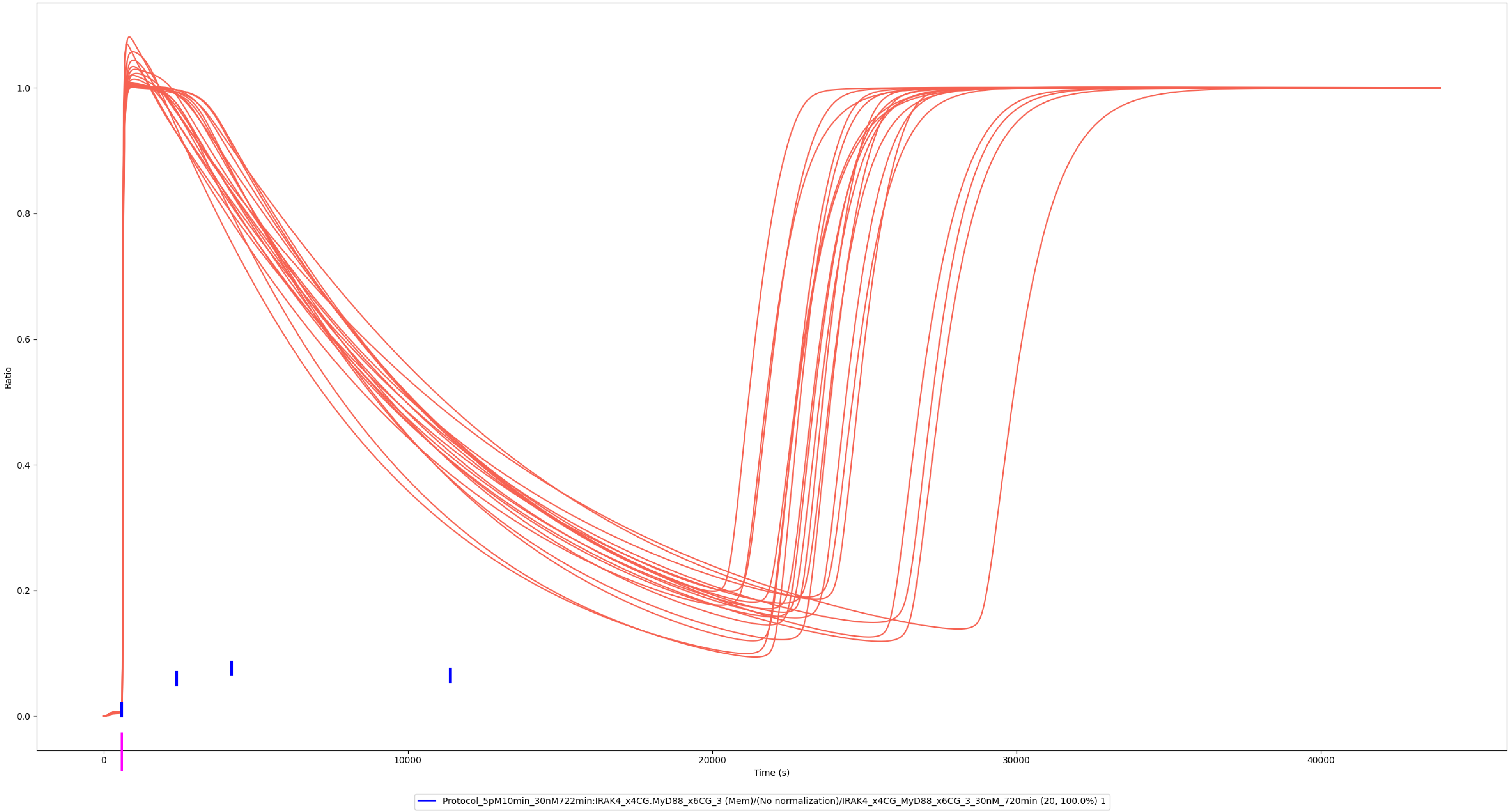

Experiment vs. Simulation: 30 nM LPS, pppp-IRAK4

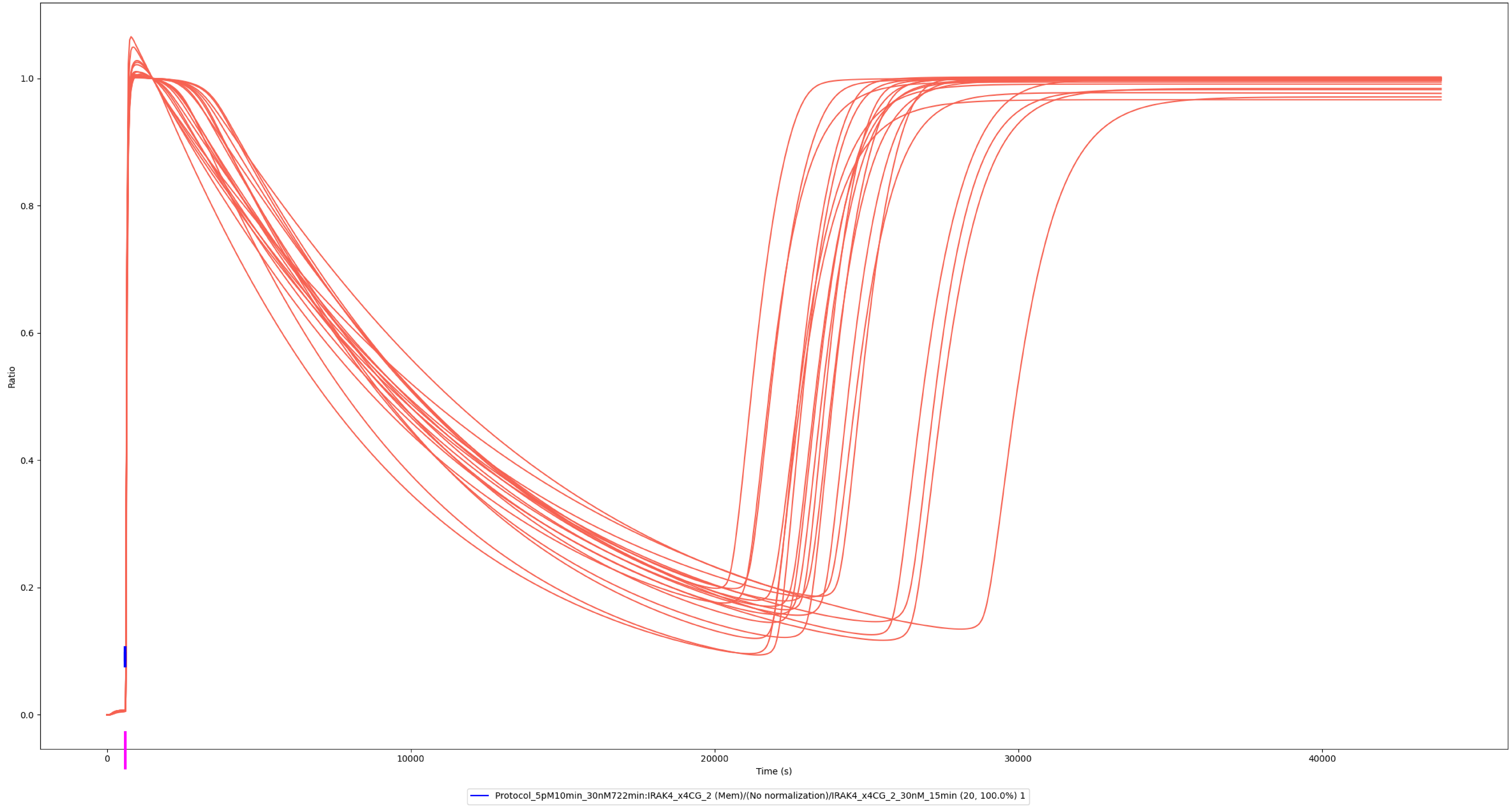

IRAK1

Experiment vs. Simulation: 30 nM LPS, IRAK4:IRAK1

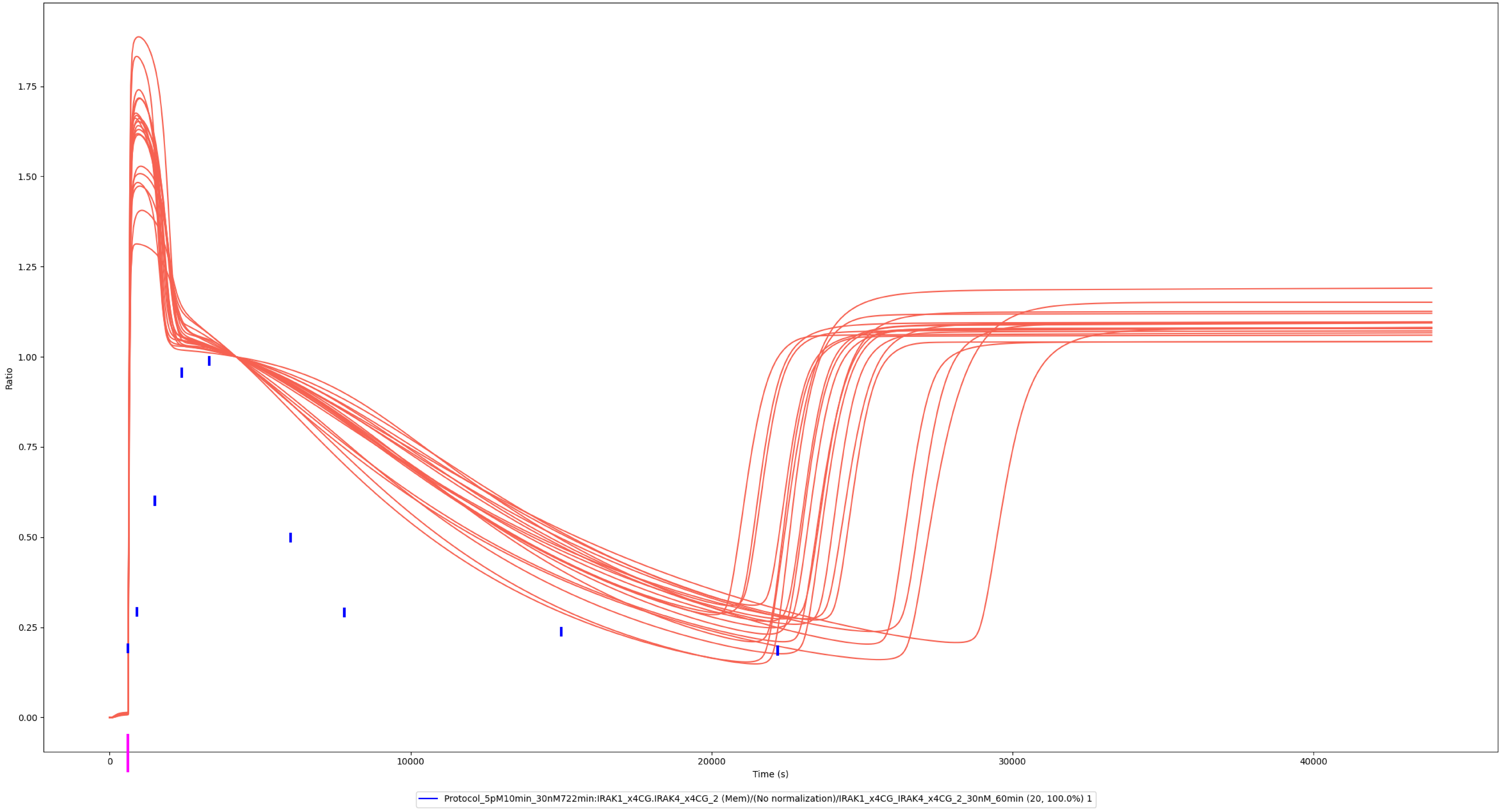

IRAK2

Experiment vs. Simulation: 30 nM LPS, IRAK4:IRAK2

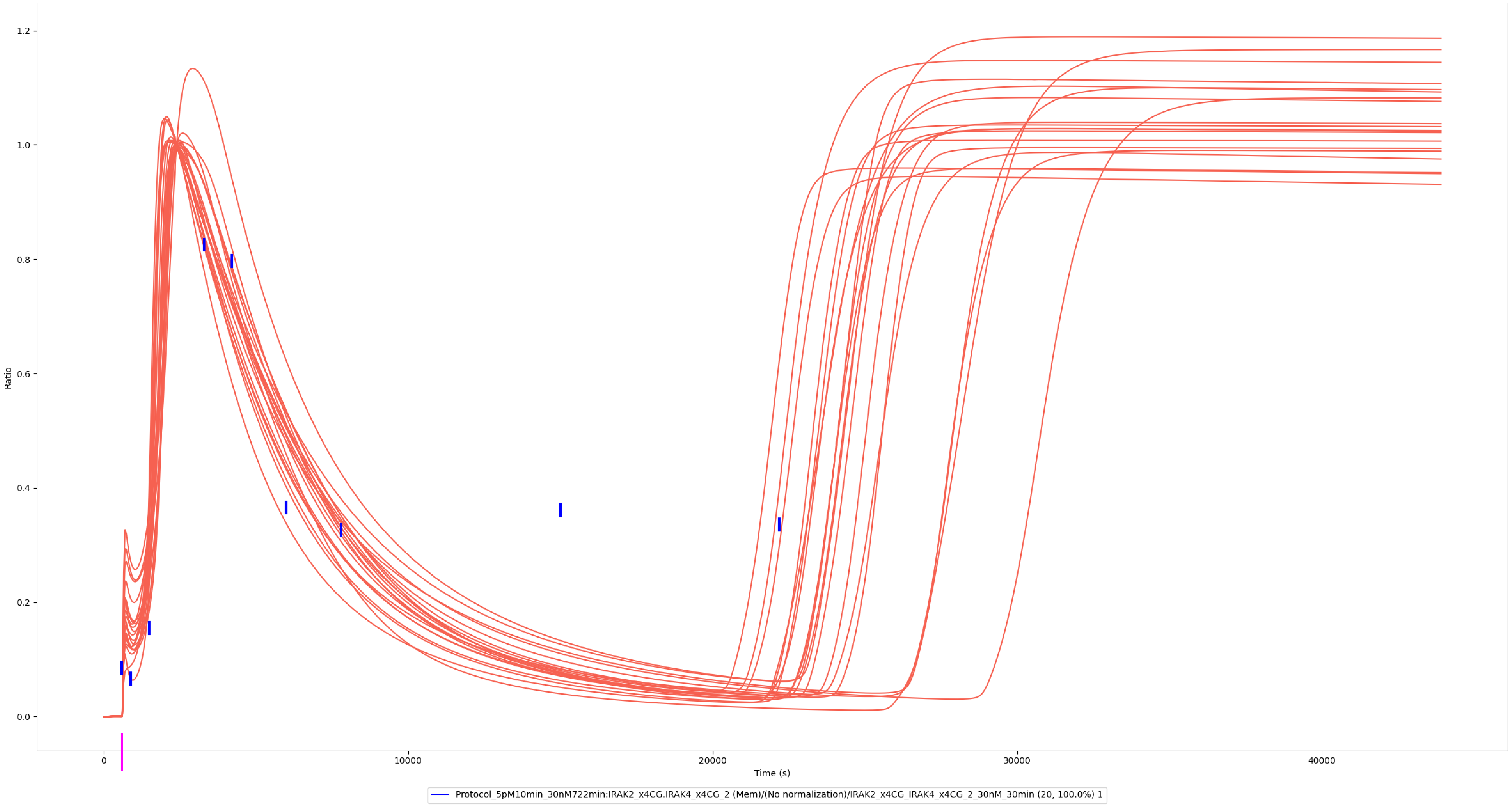

Experiment vs. Simulation: 30 nM LPS, IRAK4:IRAK2

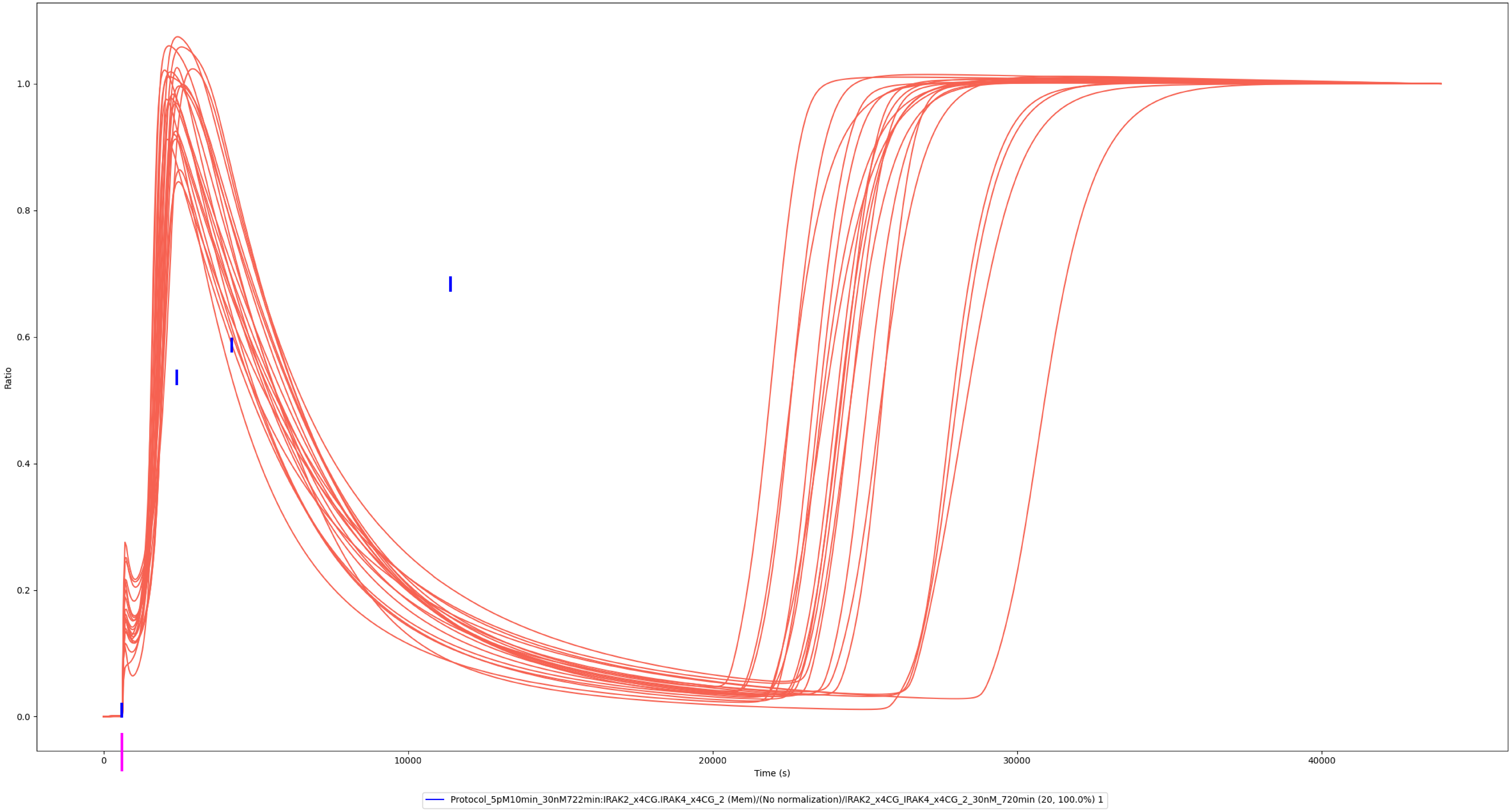

Experiment vs. Simulation: 30 nM LPS, IRAK4:pp-IRAK2

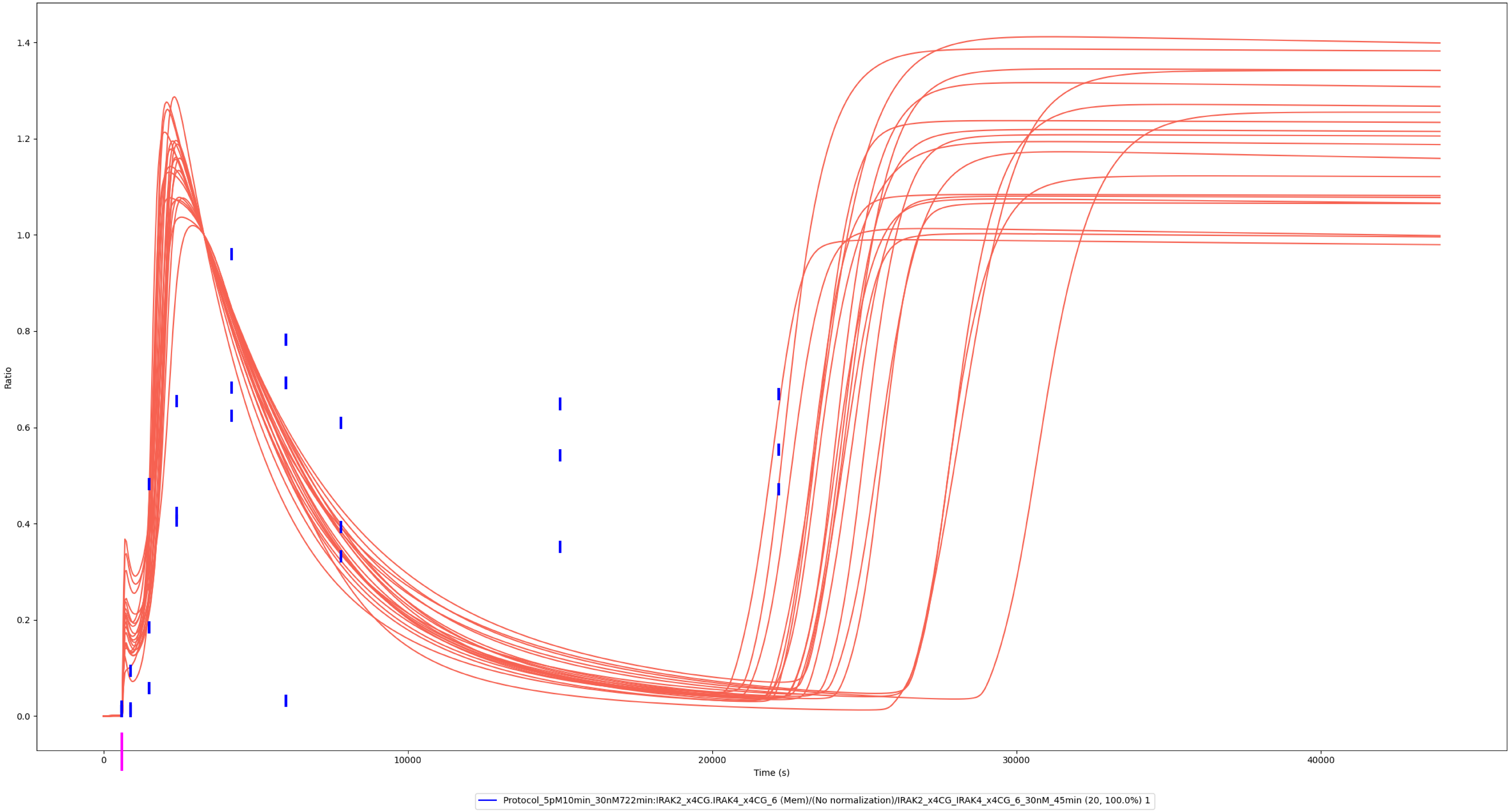

Experiment vs. Simulation: 30 nM LPS, pp-IRAK2

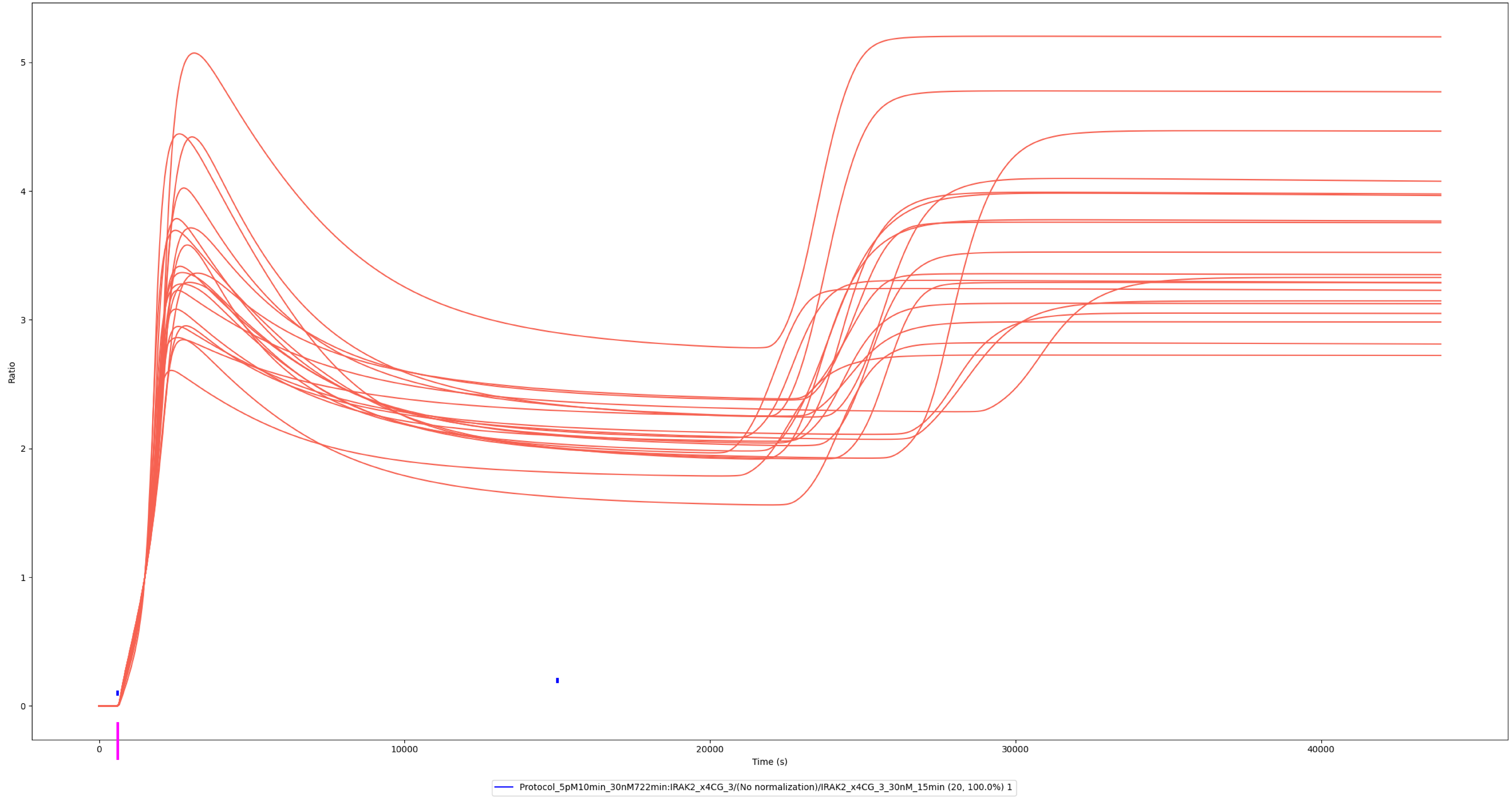

Experiment vs. Simulation: 30 nM LPS, pp-IRAK2

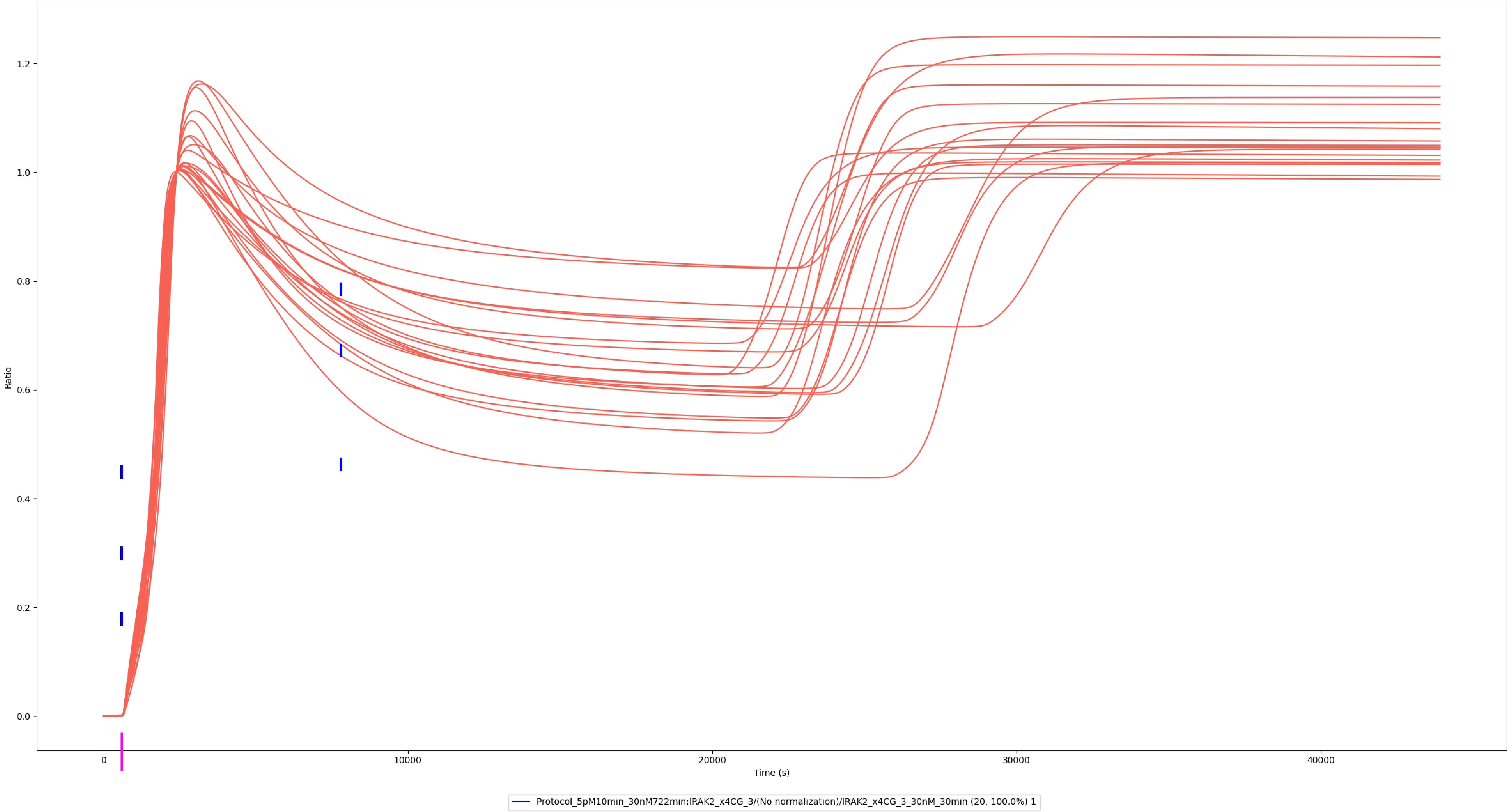

TRAF6

Experiment vs. Simulation: 30 nM LPS, IRAK1:TRAF6

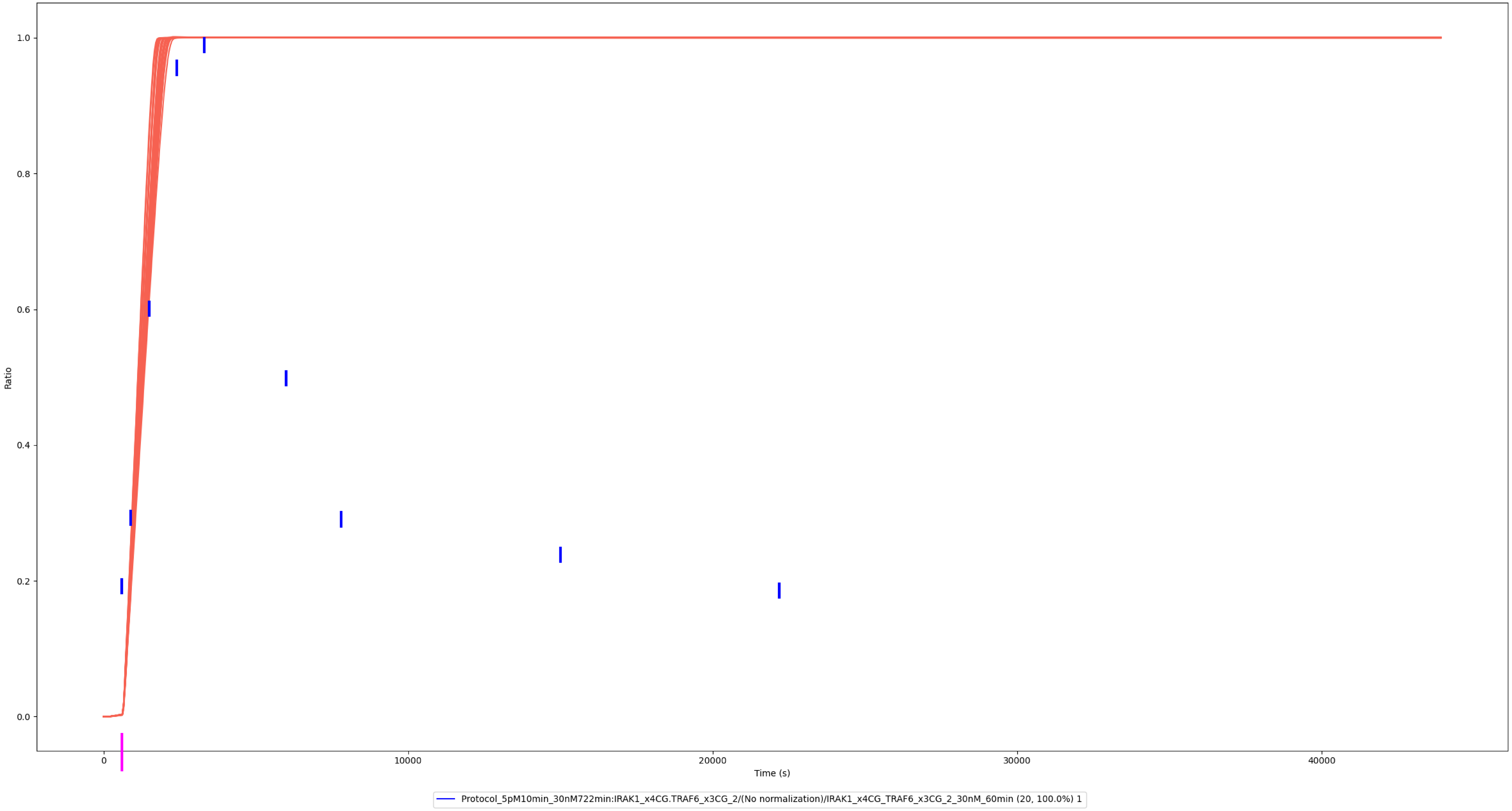

Experiment vs. Simulation: 30 nM LPS, IRAK2:TRAF6

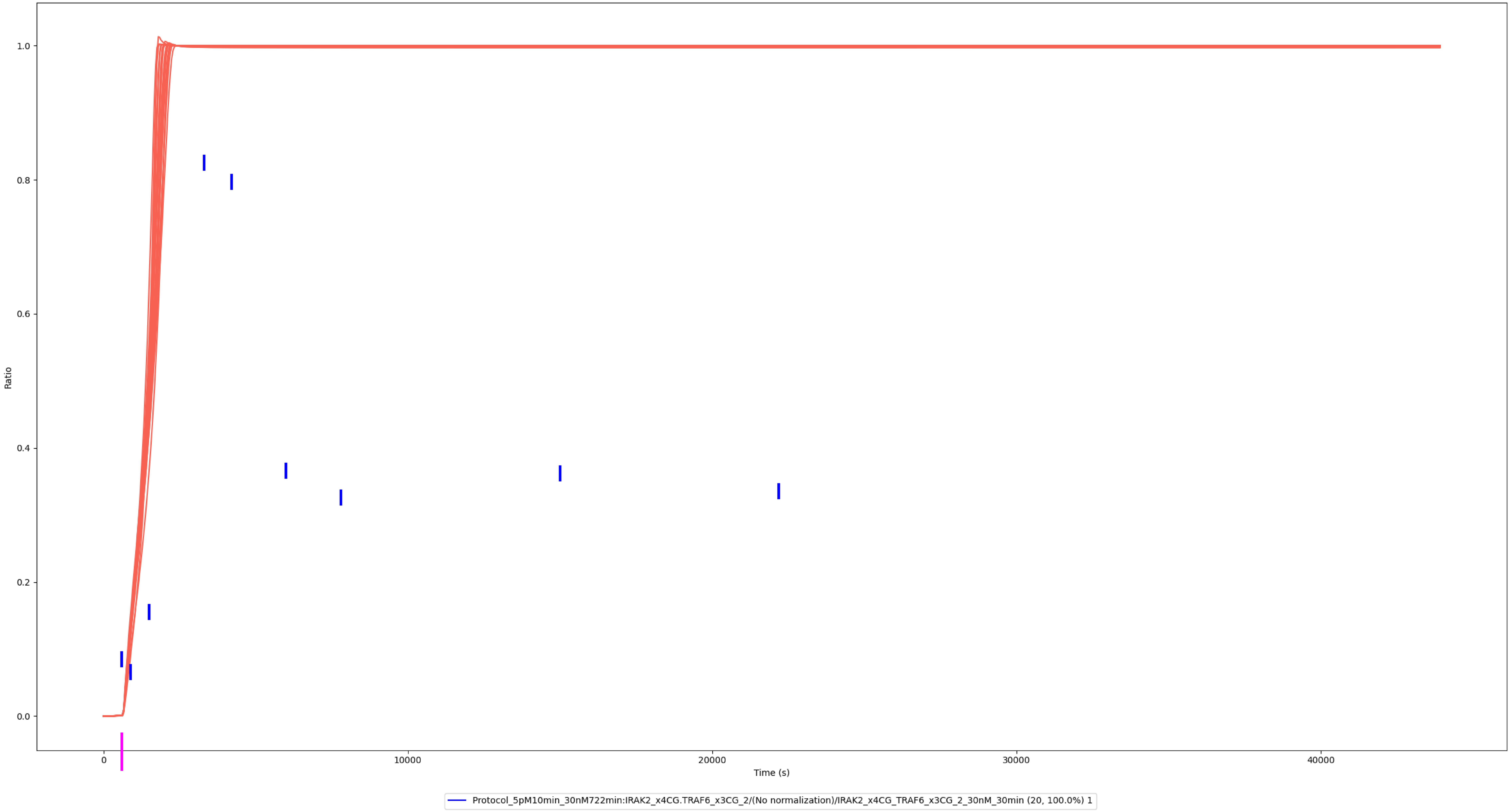

Experiment vs. Simulation: 30 nM LPS, pp-IRAK2:TRAF6

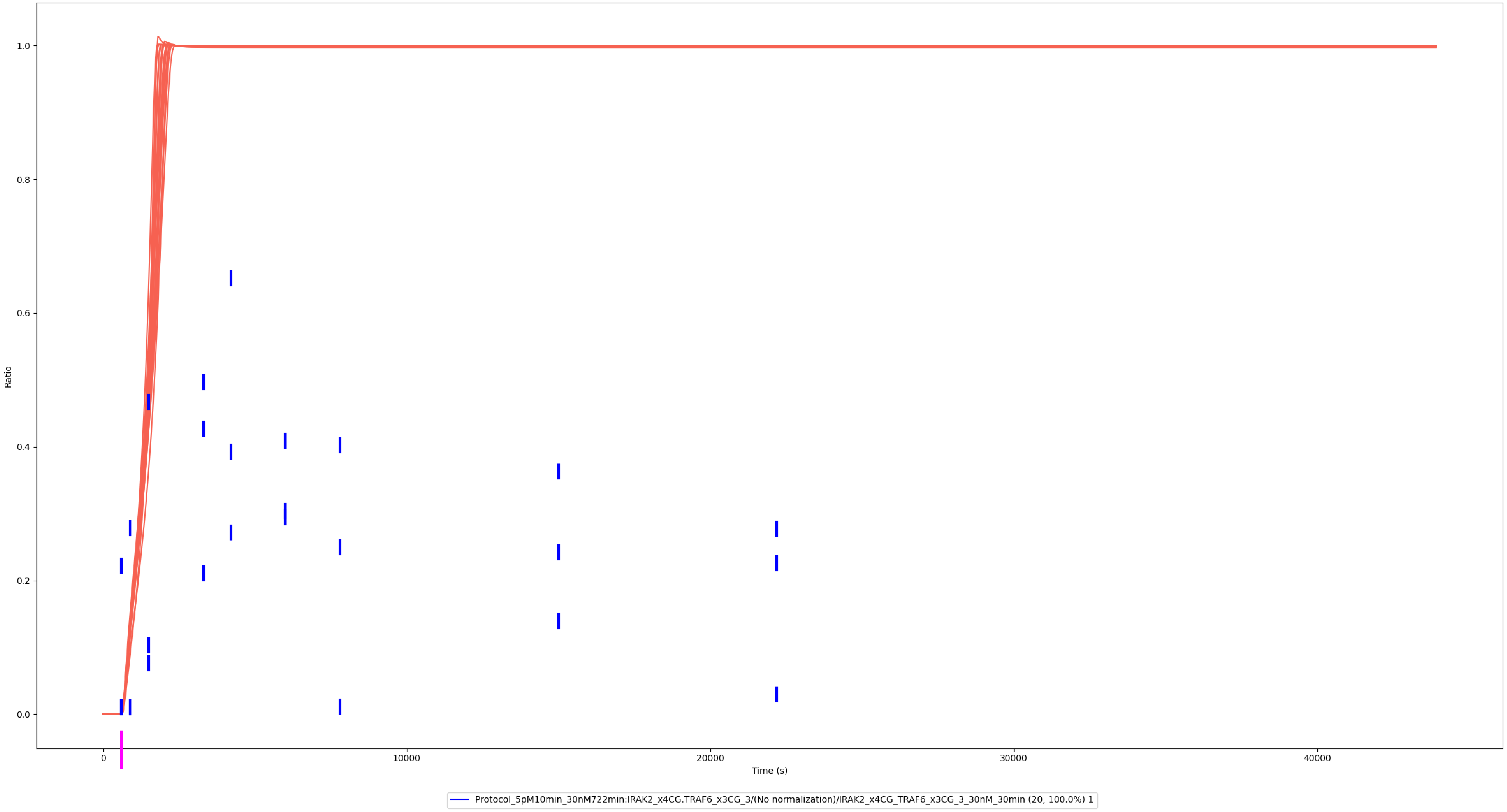

NEMO

Experiment vs. Simulation: 30 nM LPS, IRAK1:TRAF6:NEMO:IKKα/β

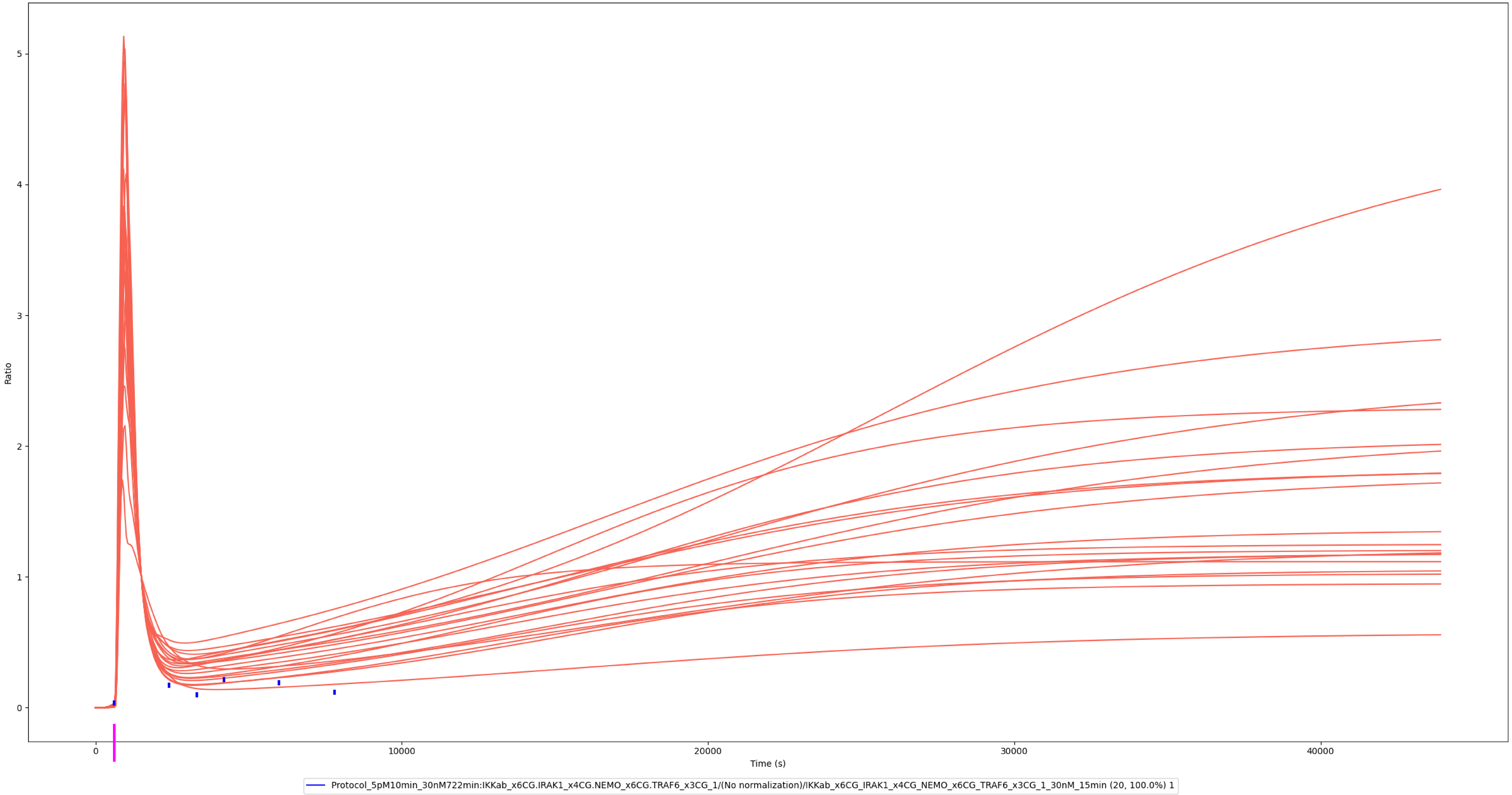

Experiment vs. Simulation: 30 nM LPS, IRAK2:TRAF6:NEMO:IKKα/β

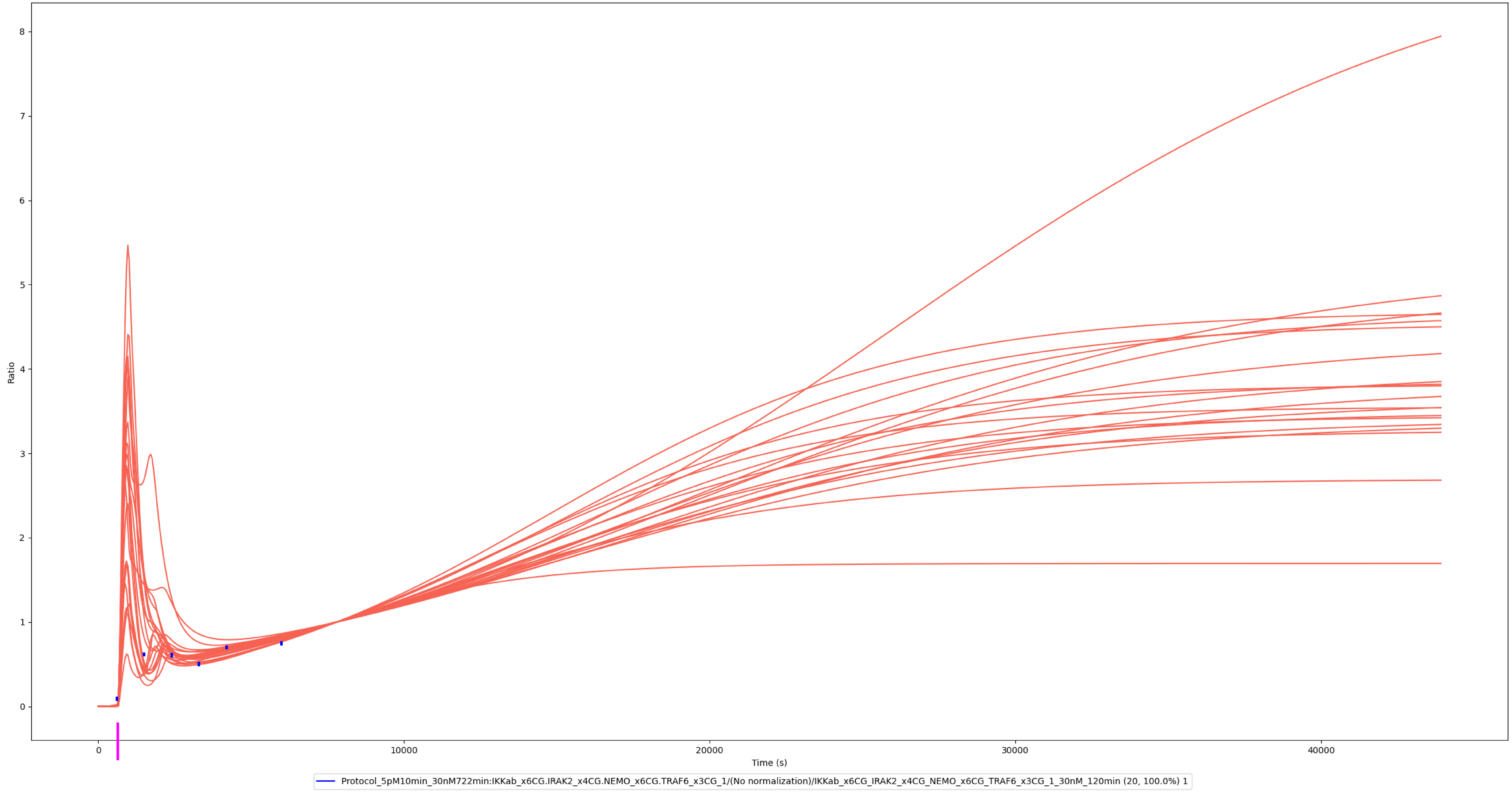

IKKα/β

Experiment vs. Simulation: 1 nM LPS, p-IKKα/β

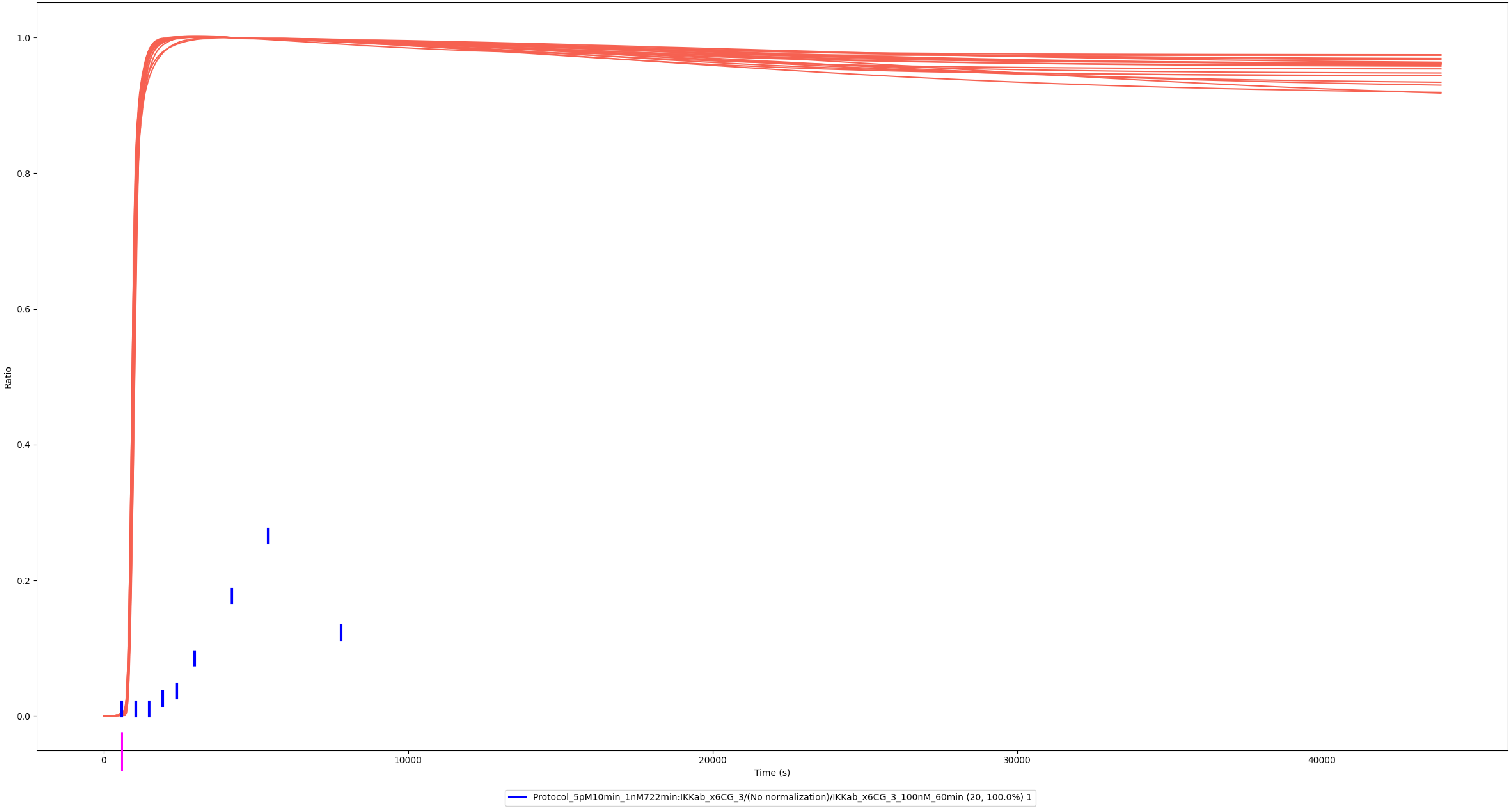

Experiment vs. Simulation: 10 nM LPS, p-IKKα/β

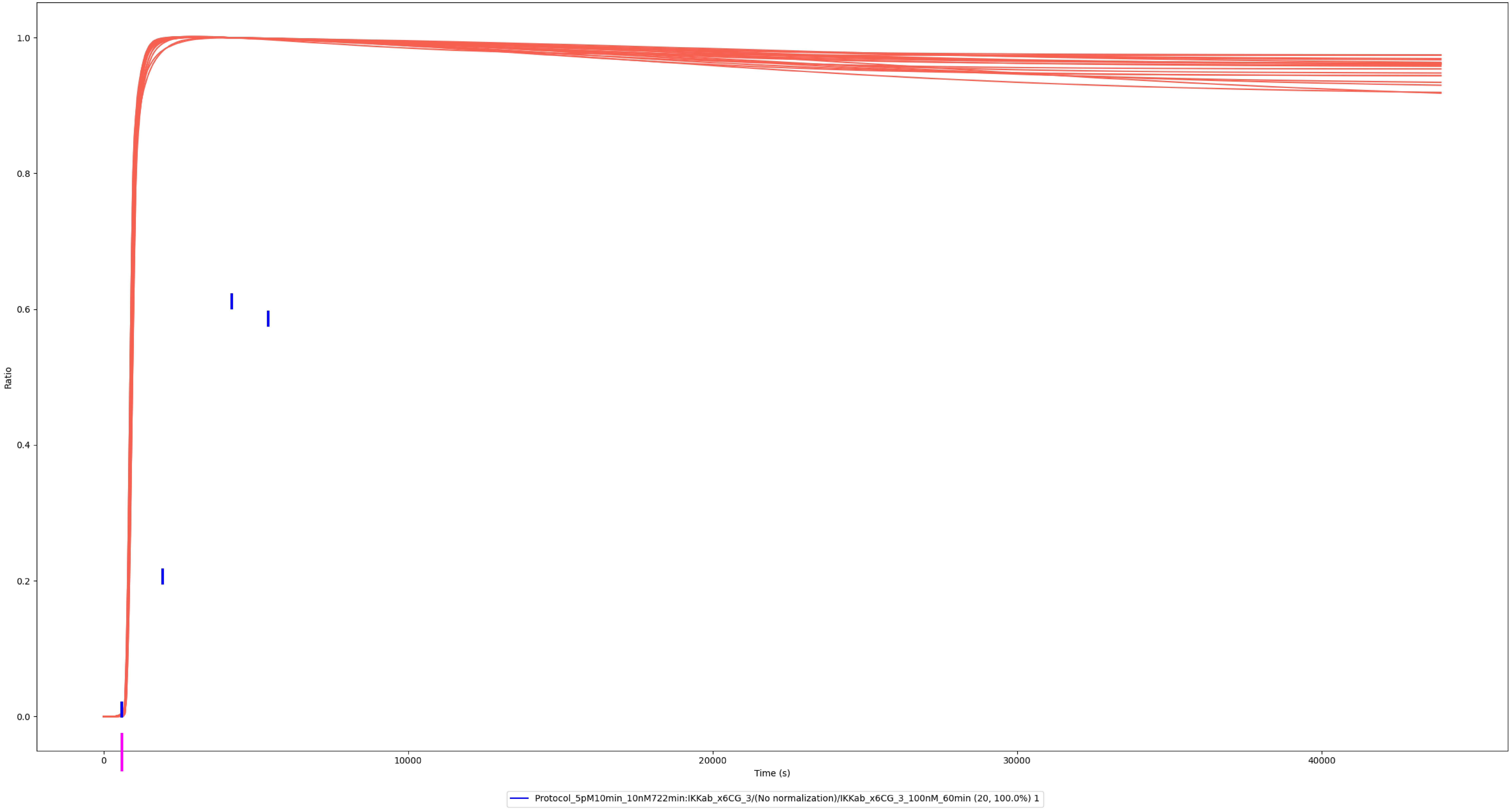

Experiment vs. Simulation: 100 nM LPS, p-IKKα/β

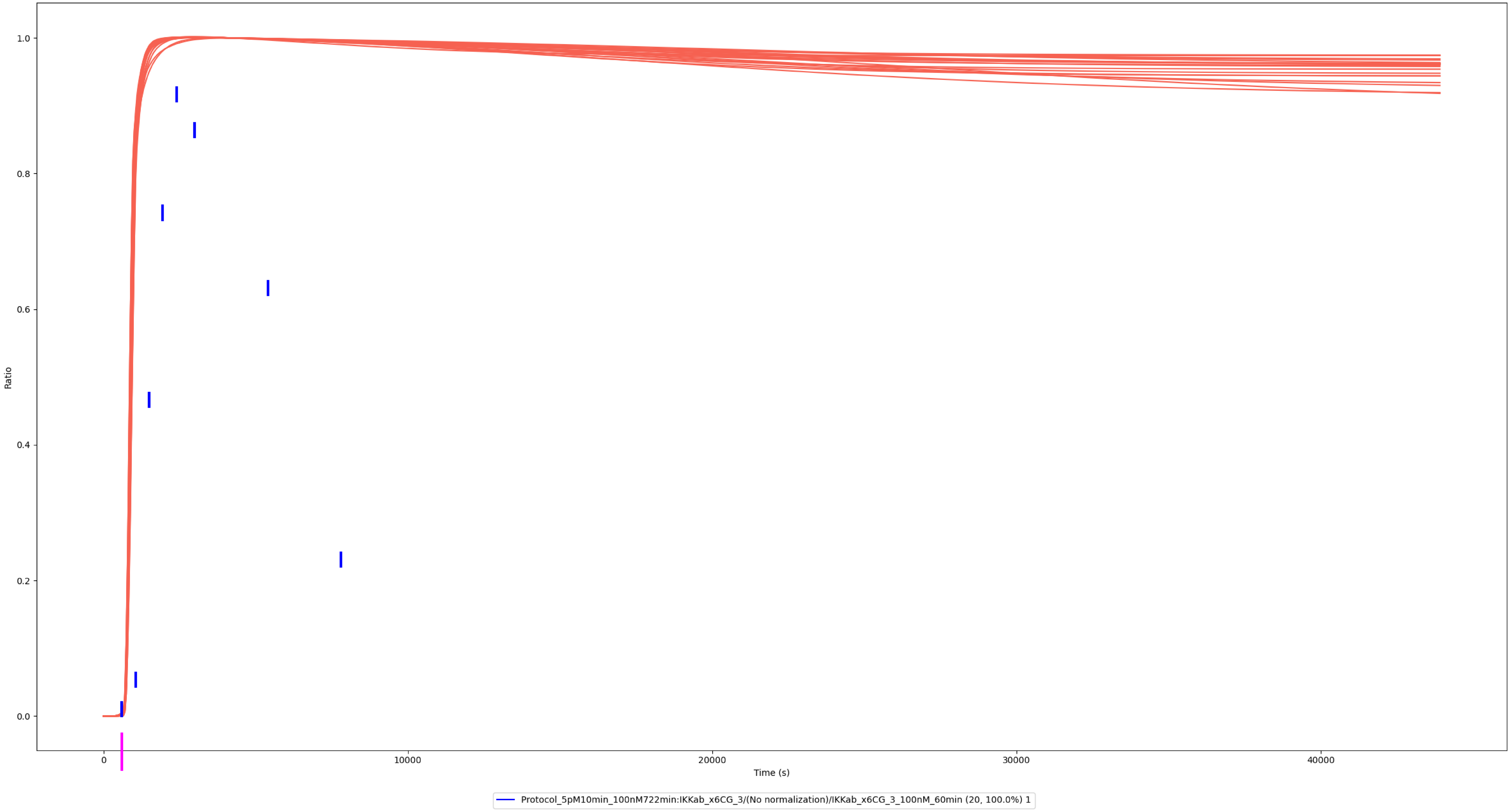

Experiment vs. Simulation: 1.5 nM LPS, pp-IKKα/β

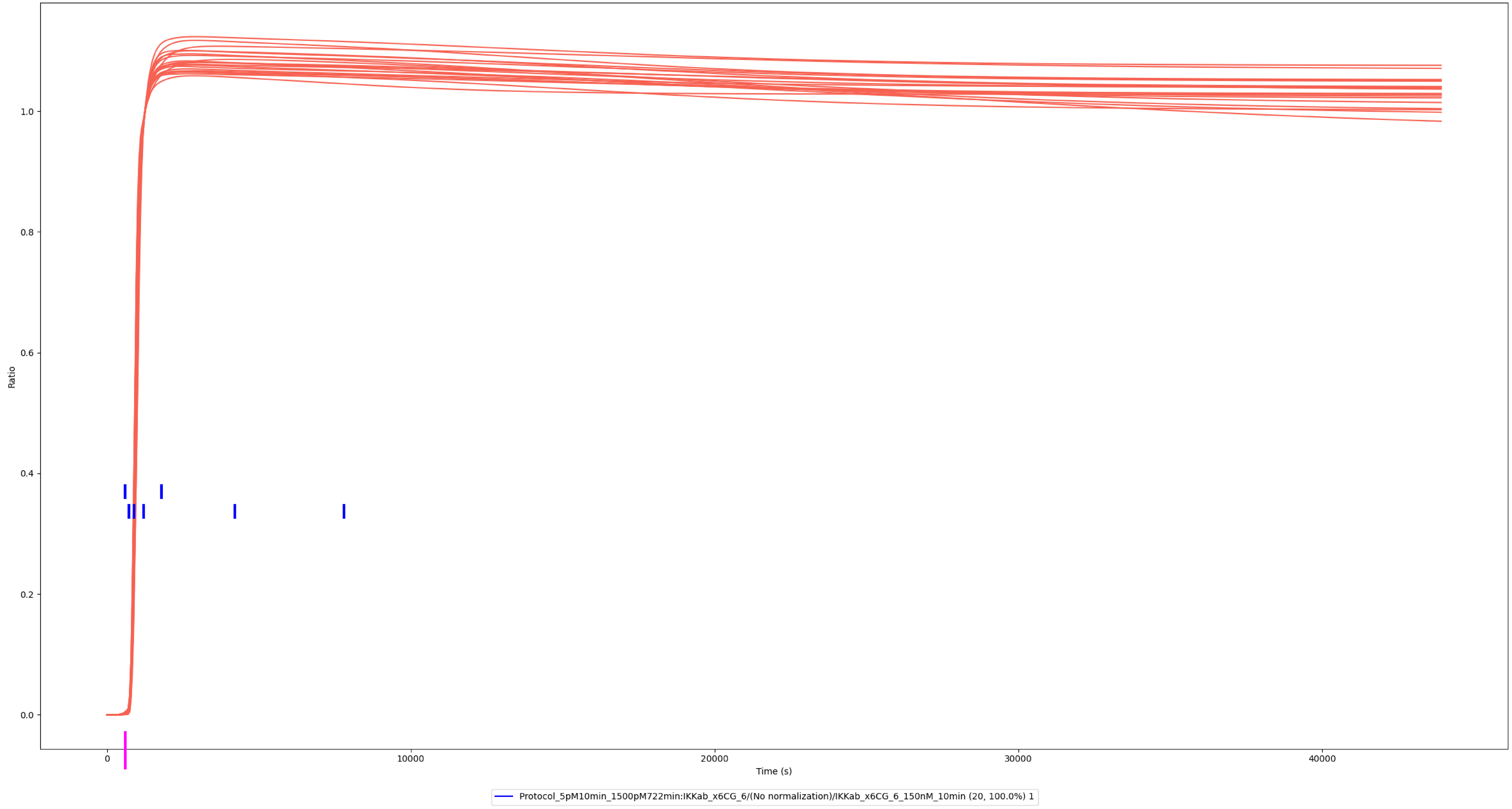

Experiment vs. Simulation: 15 nM LPS, pp-IKKα/β

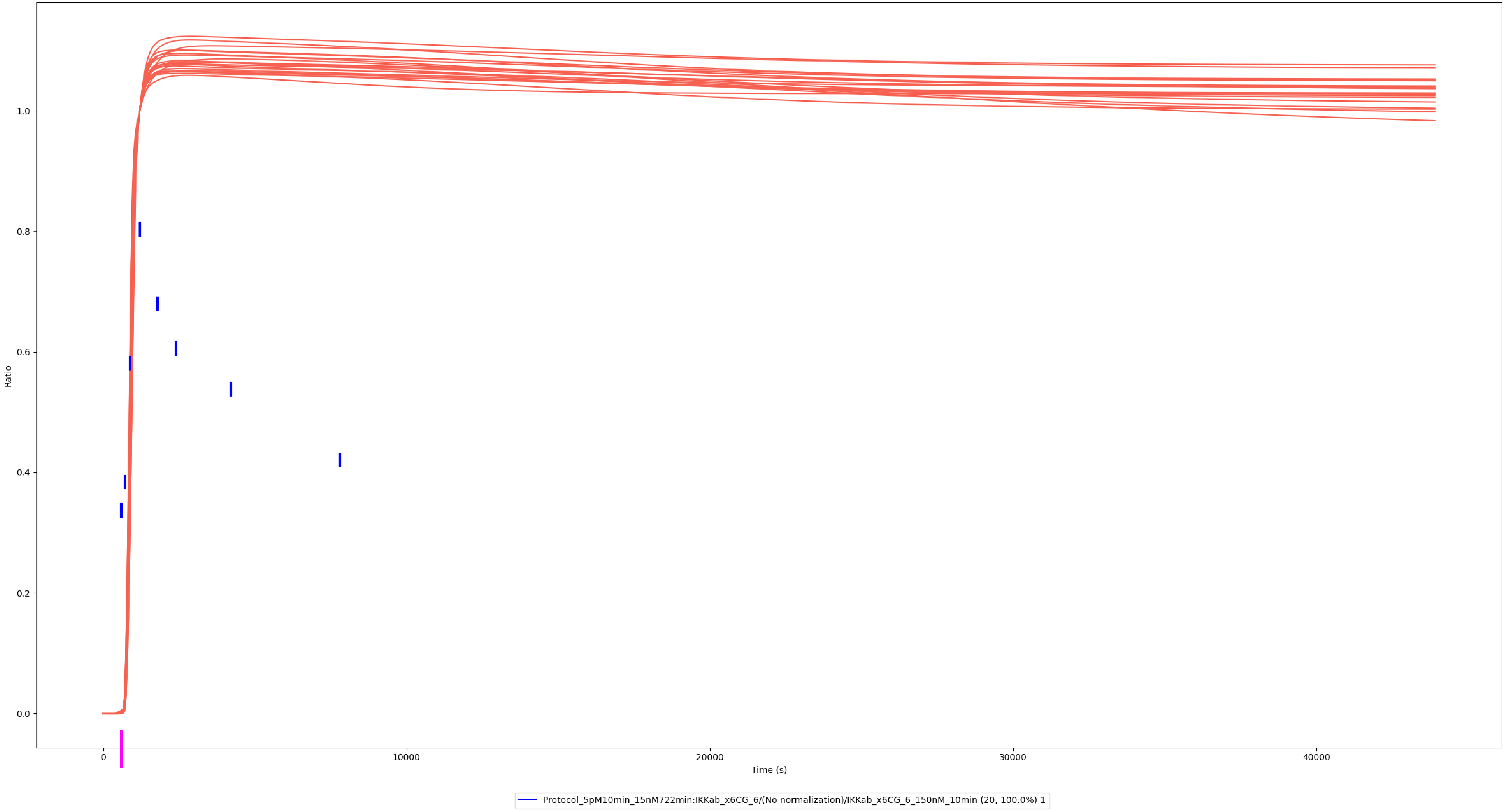

Experiment vs. Simulation: 150 nM LPS, pp-IKKα/β

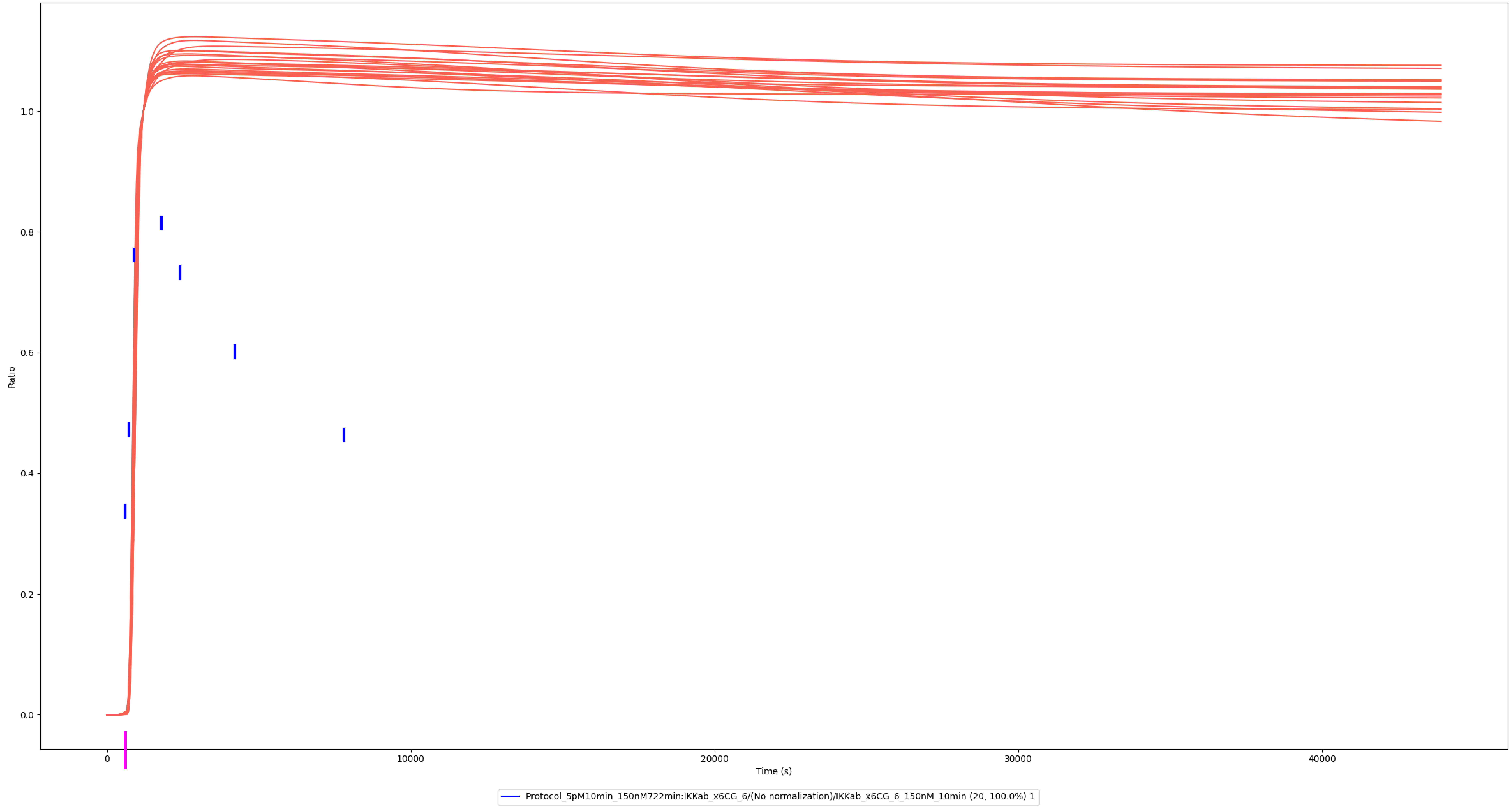

IκBα

Experiment vs. Simulation: 0.01 nM LPS, IκBα

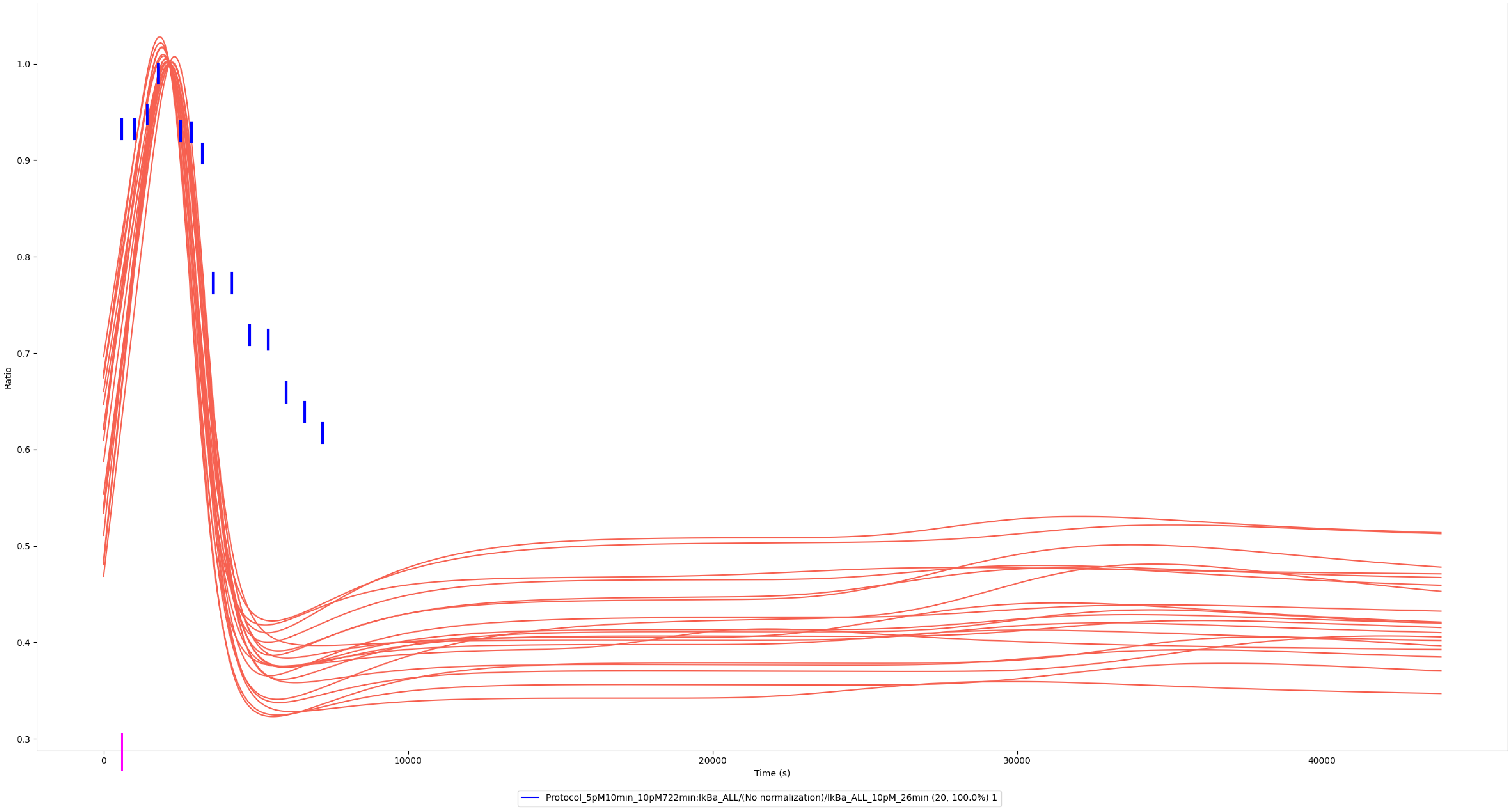

Experiment vs. Simulation: 0.1 nM LPS, IκBα

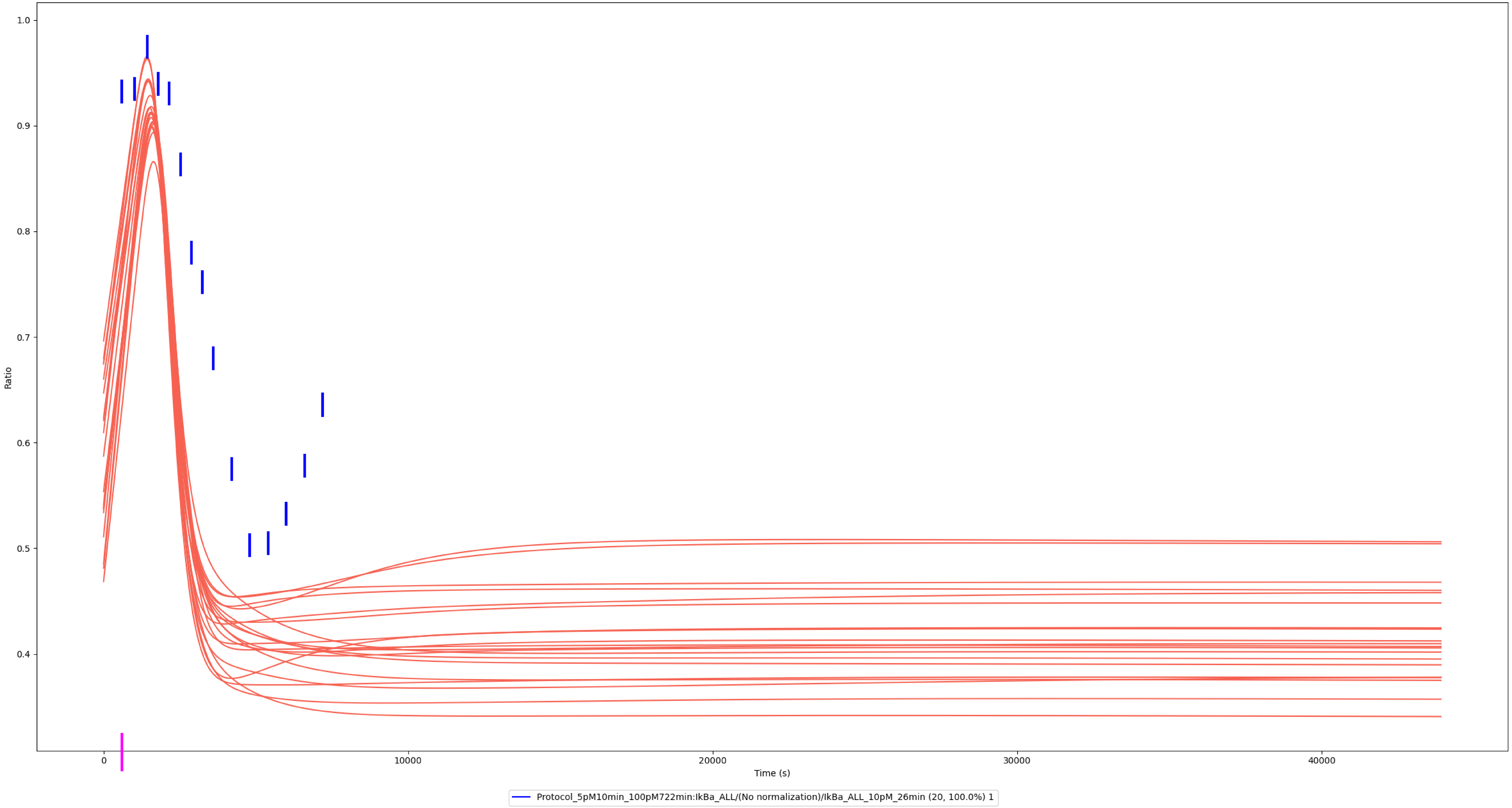

Experiment vs. Simulation: 1 nM LPS, IκBα

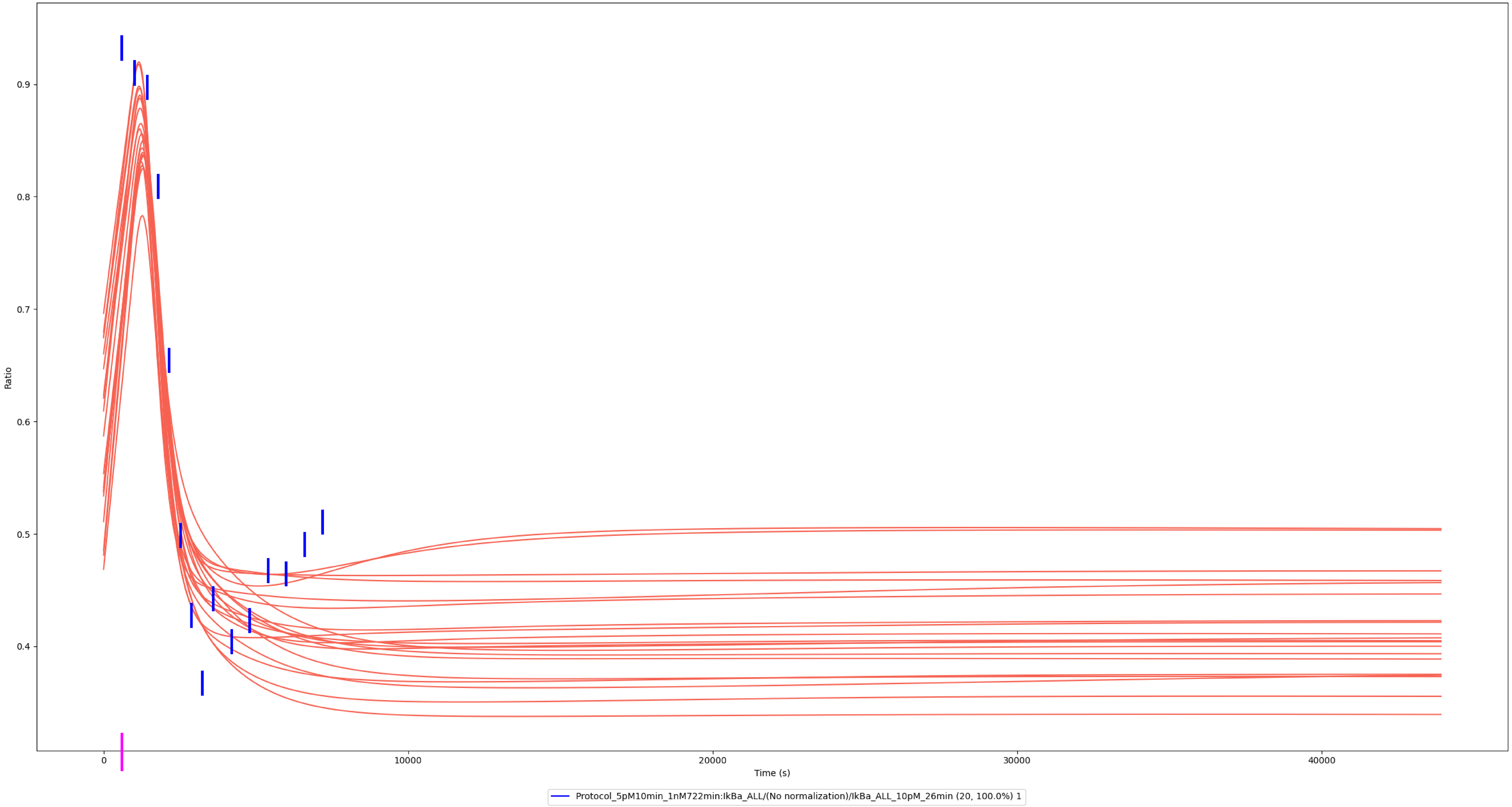

Experiment vs. Simulation: 10 nM LPS, IκBα

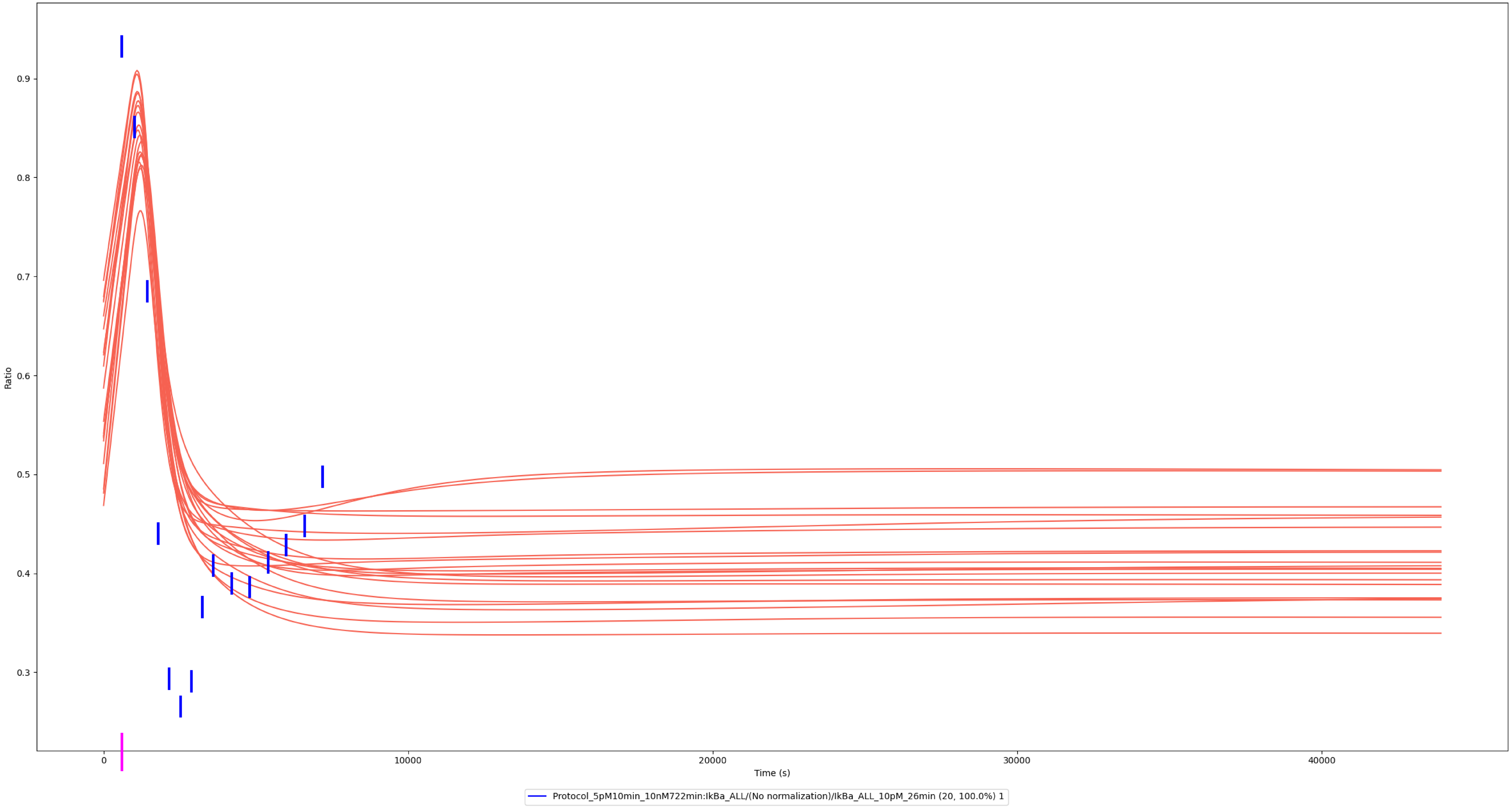

Experiment vs. Simulation: 100 nM LPS, IκBα

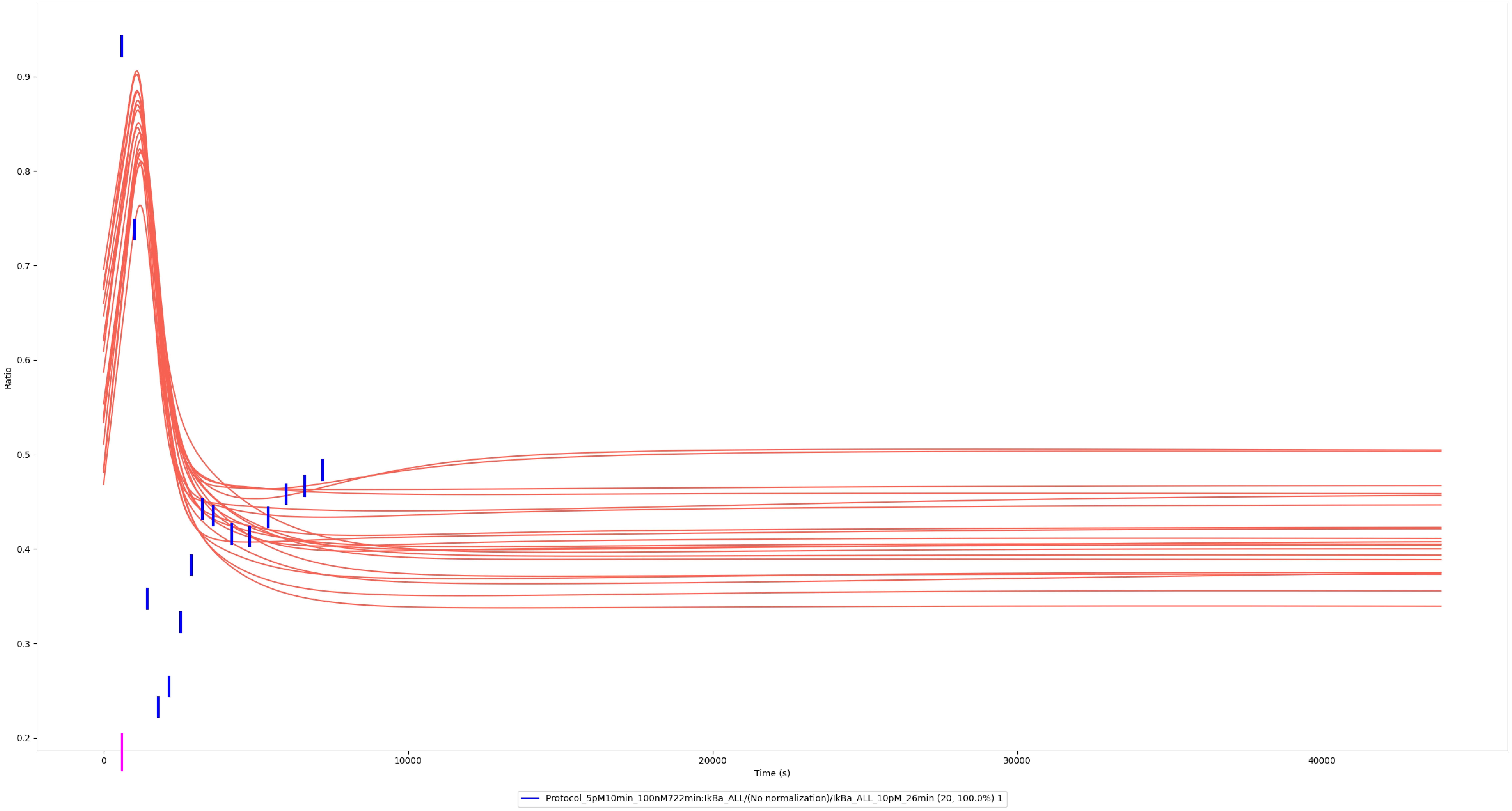

Experiment vs. Simulation: 1,000 nM LPS, IκBα

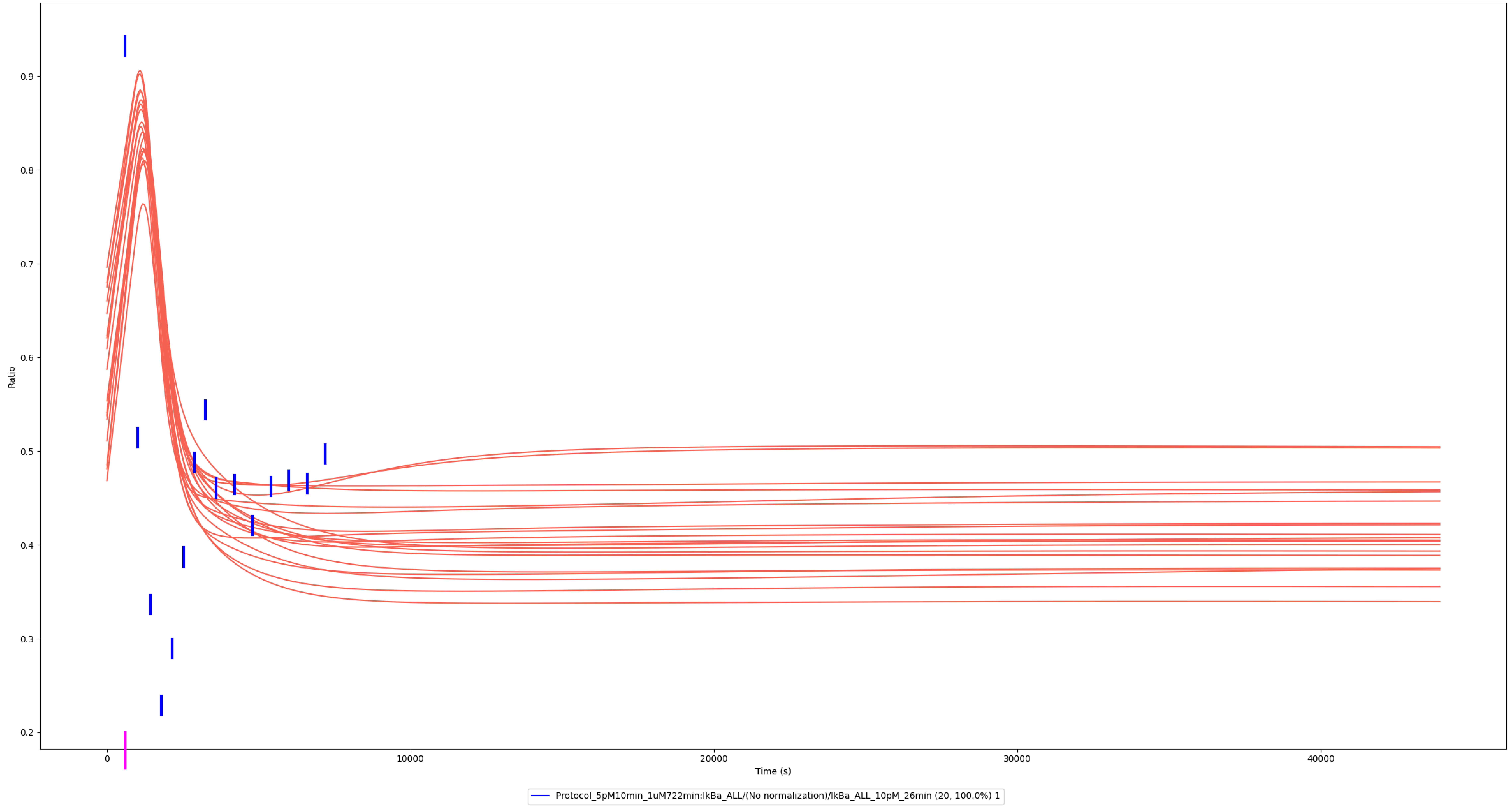

Experiment vs. Simulation: 1 nM LPS, IκBα

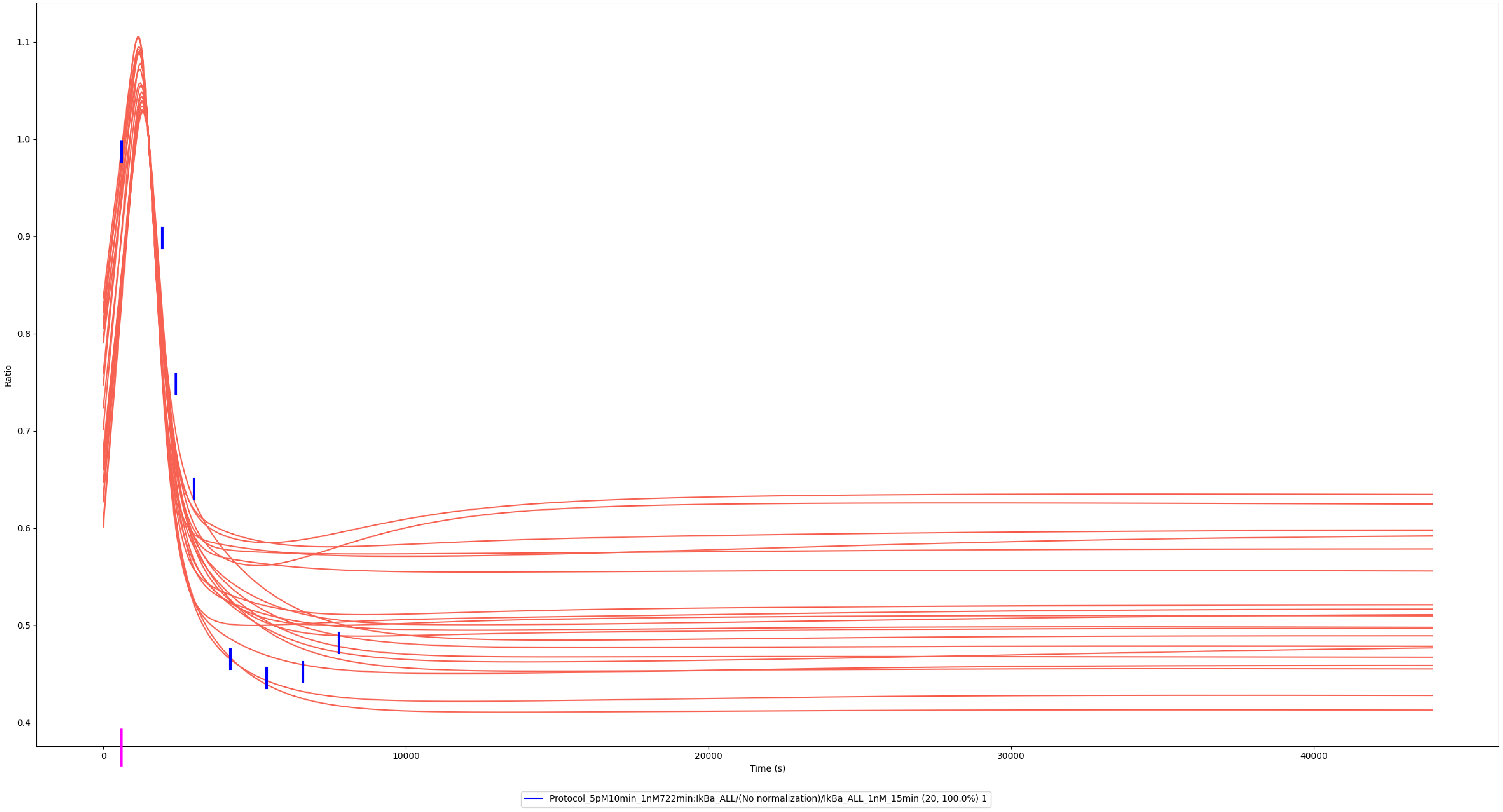

Experiment vs. Simulation: 100 nM LPS, IκBα

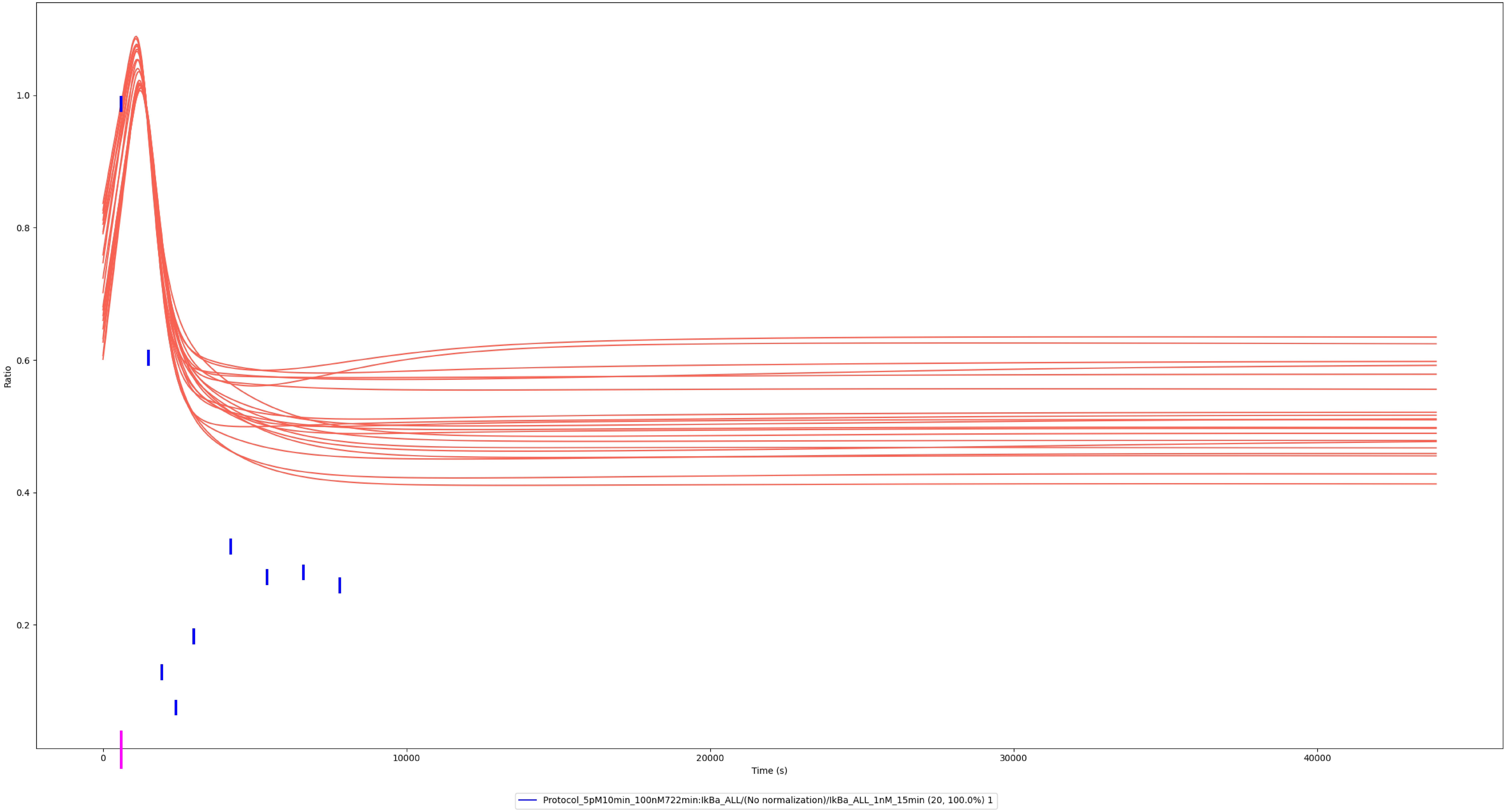

RelA

Experiment vs. Simulation: 0.3 nM LPS, Nuclear / Total RelA

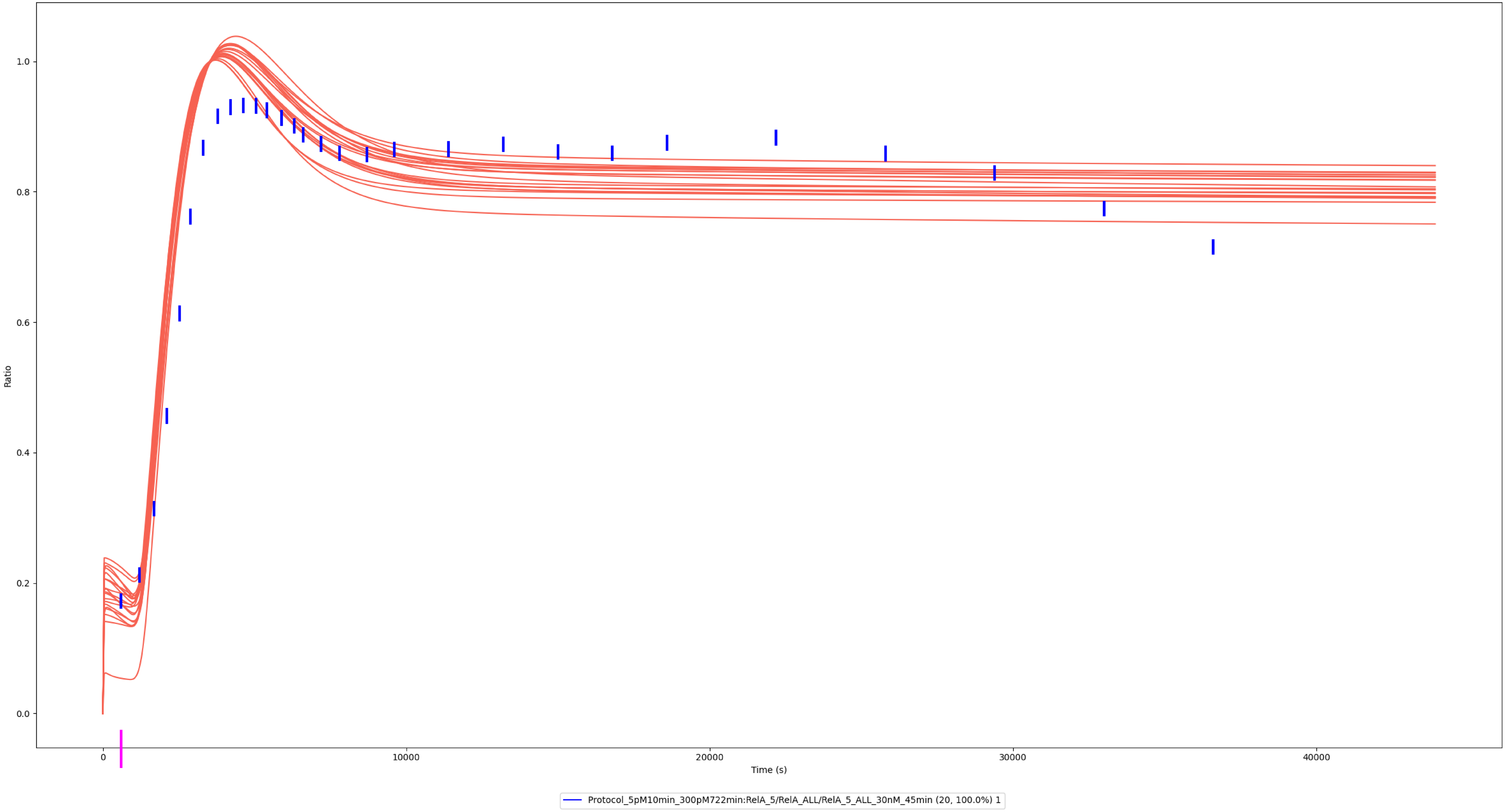

Experiment vs. Simulation: 3 nM LPS, Nuclear / Total RelA

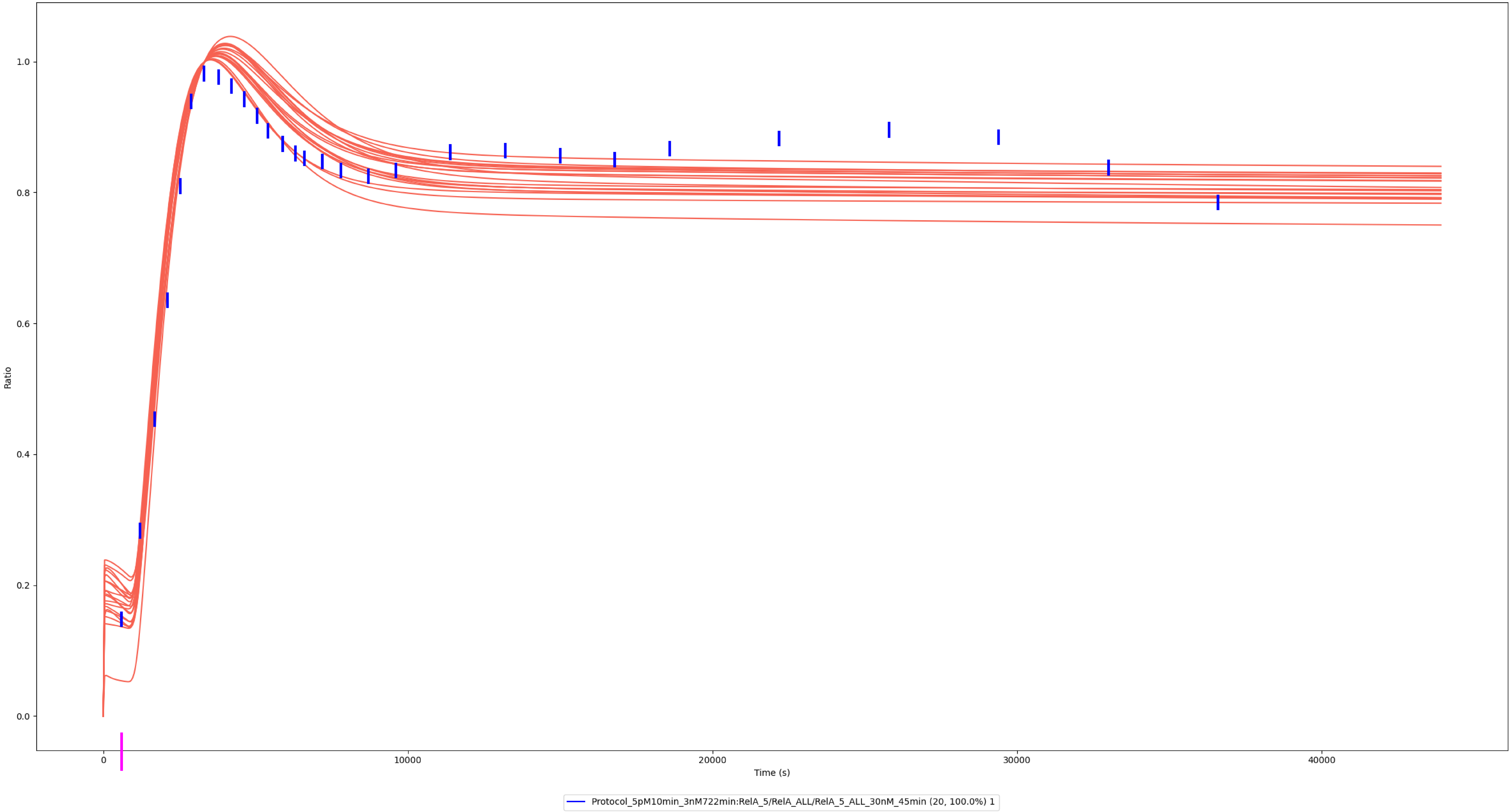

Experiment vs. Simulation: 30 nM LPS, Nuclear / Total RelA

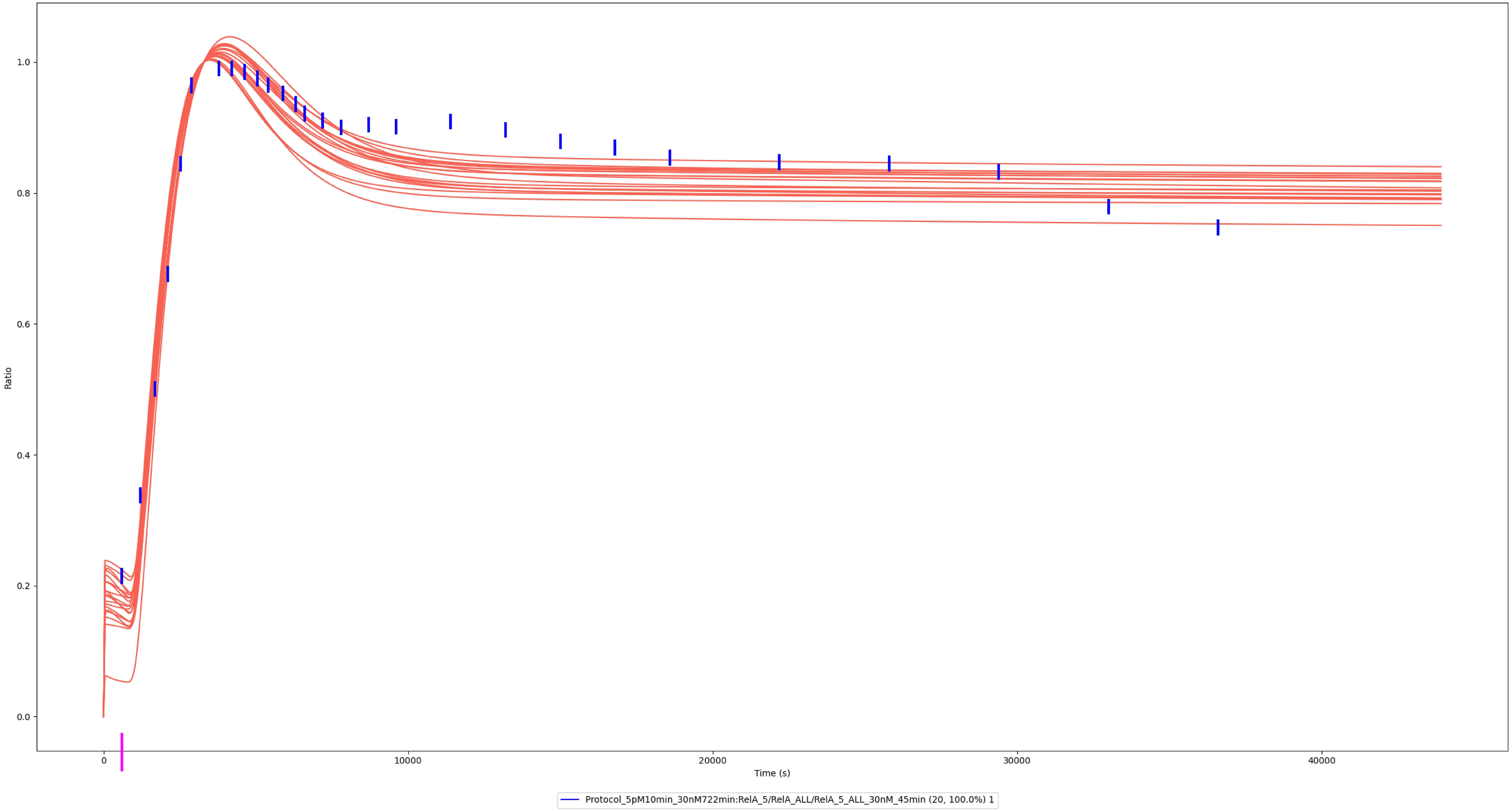

Experiment vs. Simulation: 150 nM LPS, Nuclear / Total RelA

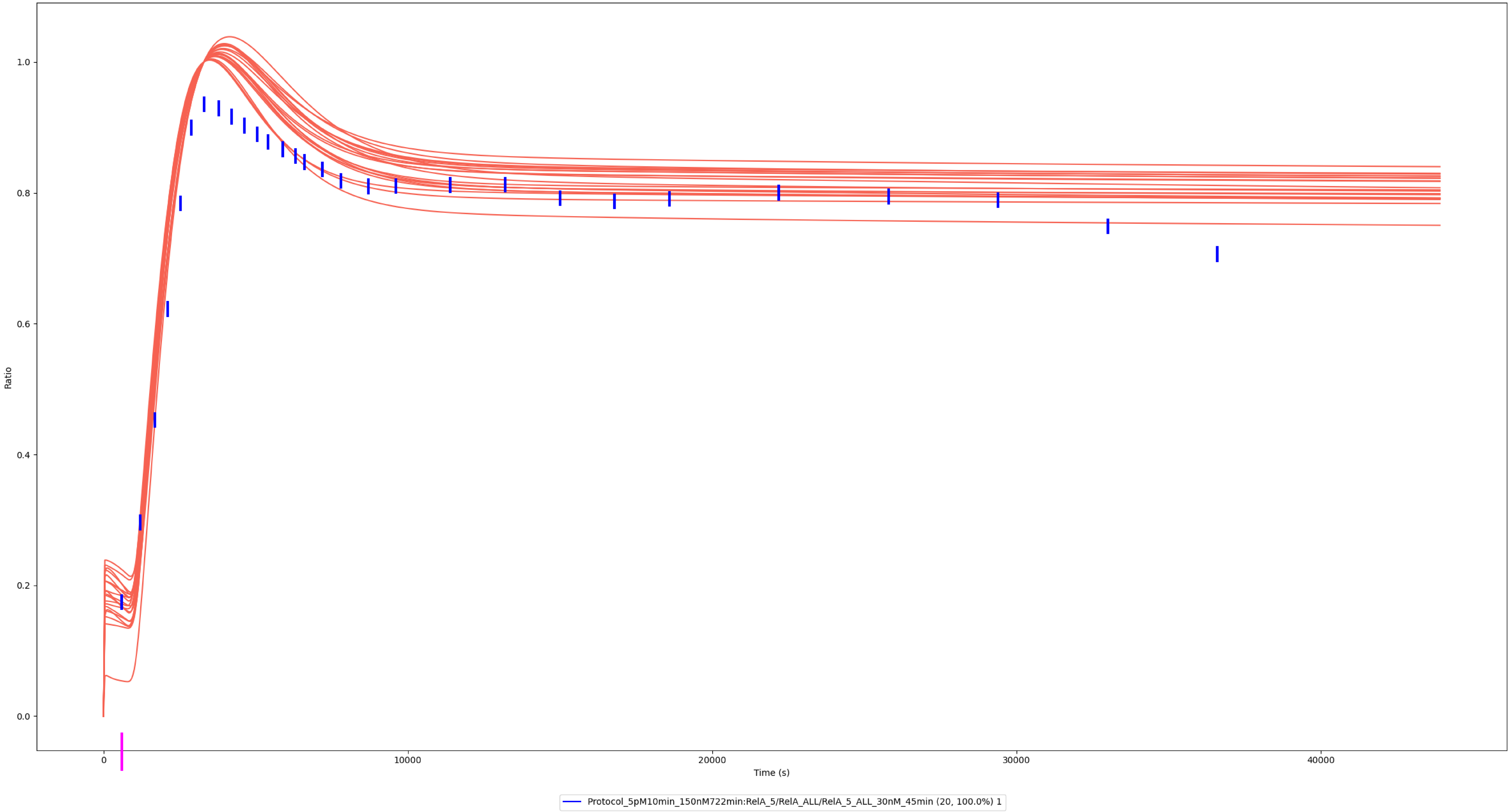

cRel

Experiment vs. Simulation: 0.3 nM LPS, Nuclear / Total cRel

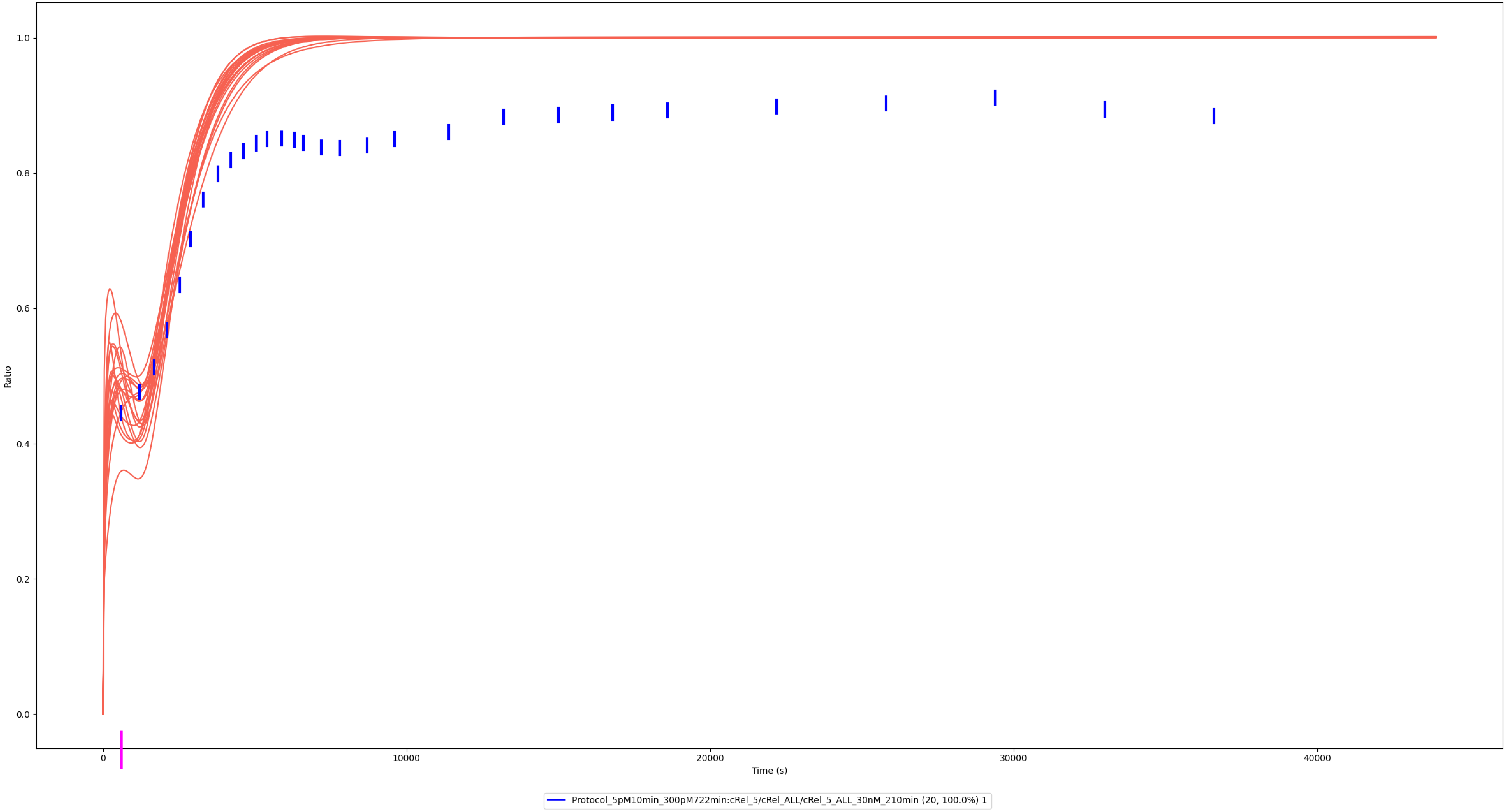

Experiment vs. Simulation: 3 nM LPS, Nuclear / Total cRel

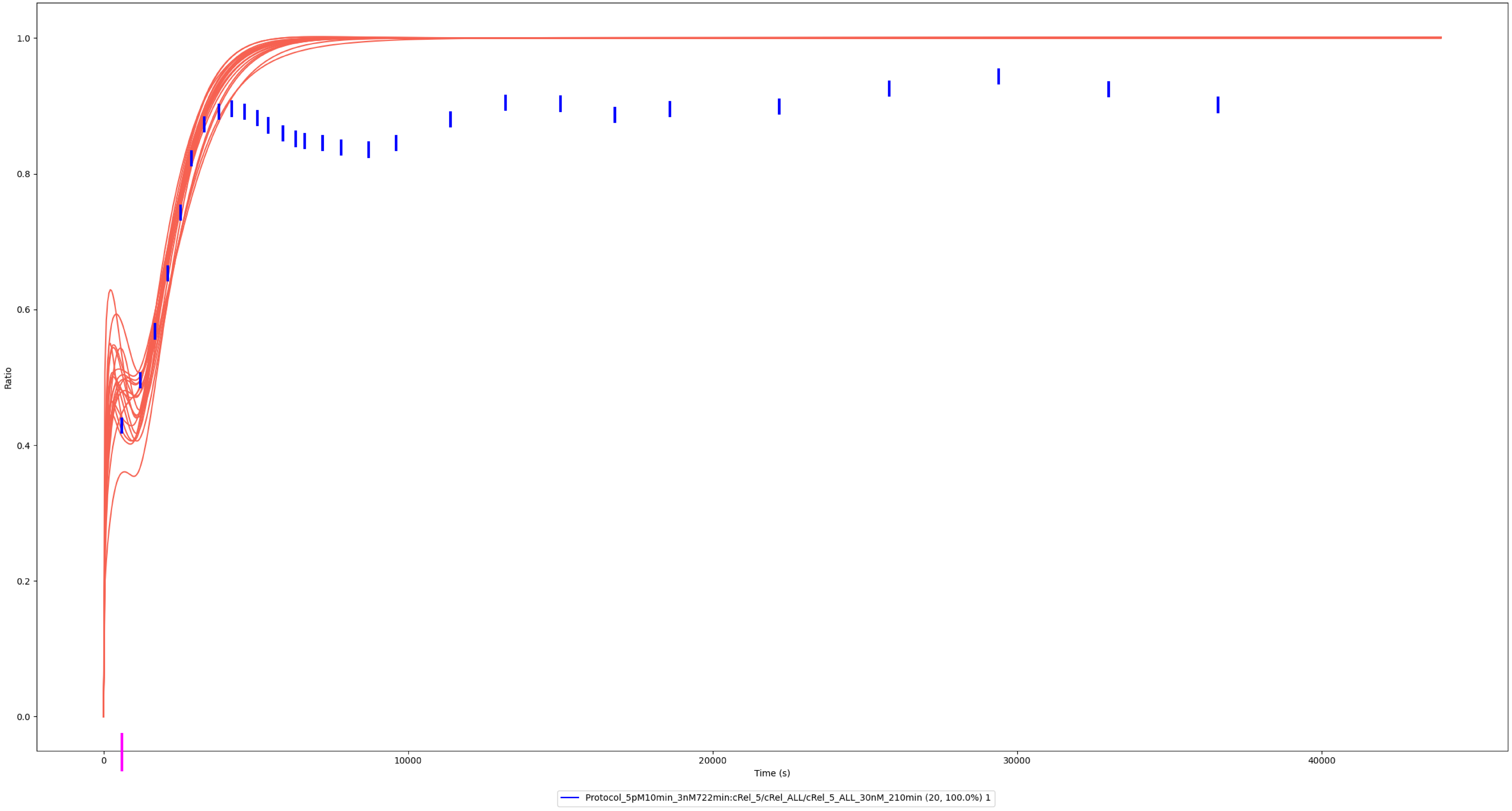

Experiment vs. Simulation: 30 nM LPS, Nuclear / Total cRel

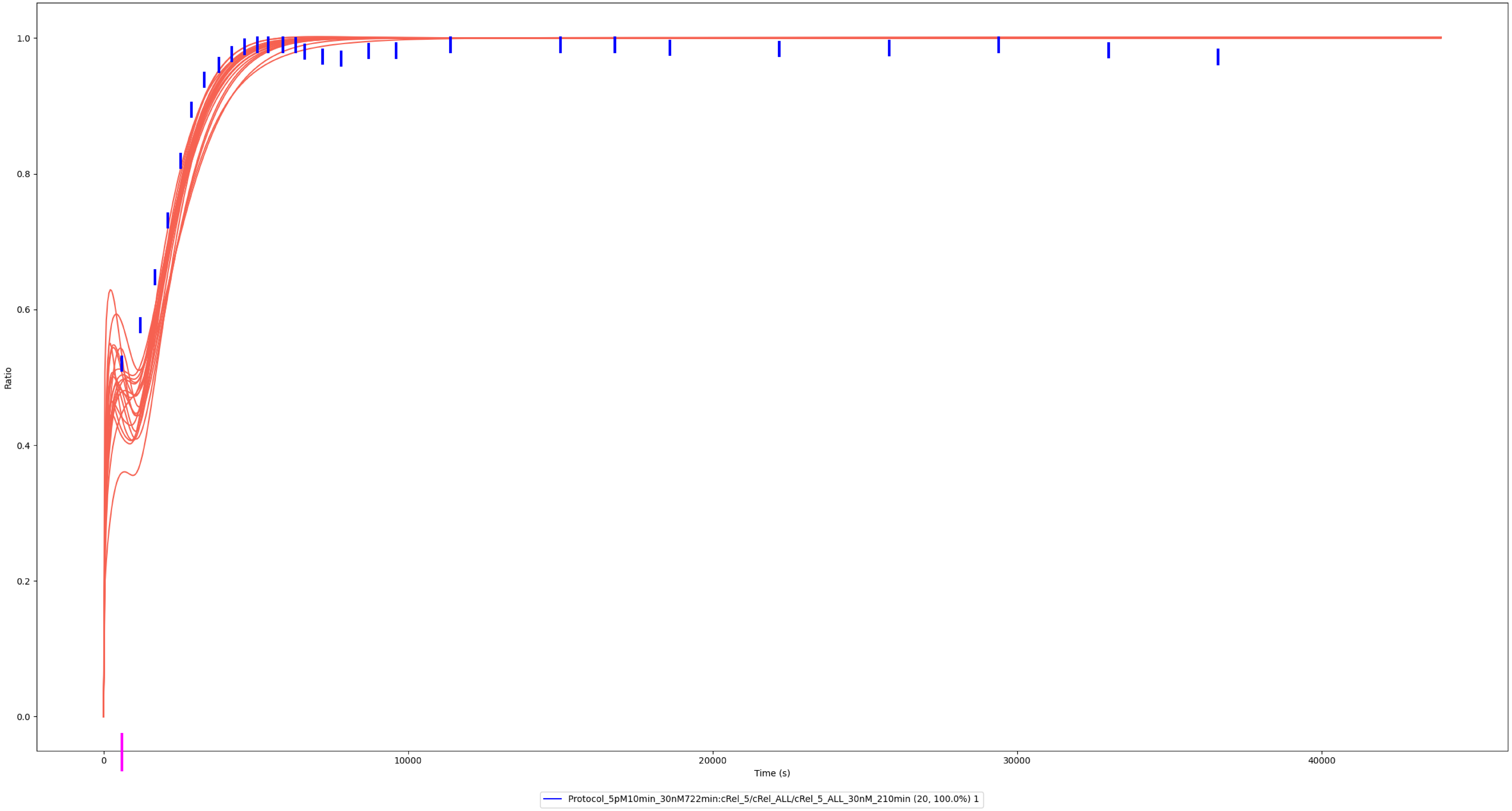

Experiment vs. Simulation: 150 nM LPS, Nuclear / Total cRel

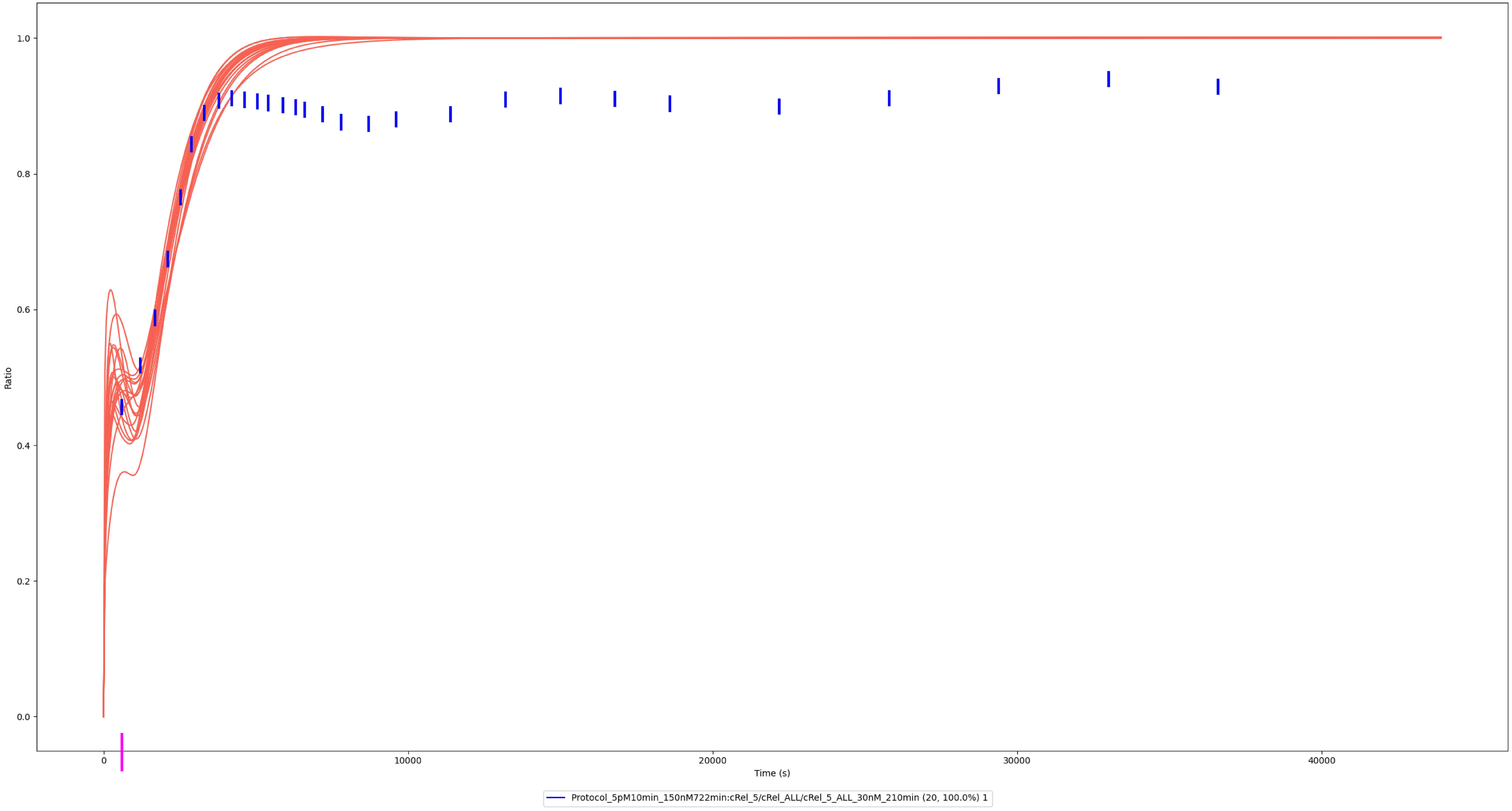

MKK1/2

Experiment vs. Simulation: 1 nM LPS, pp-MKK1/2

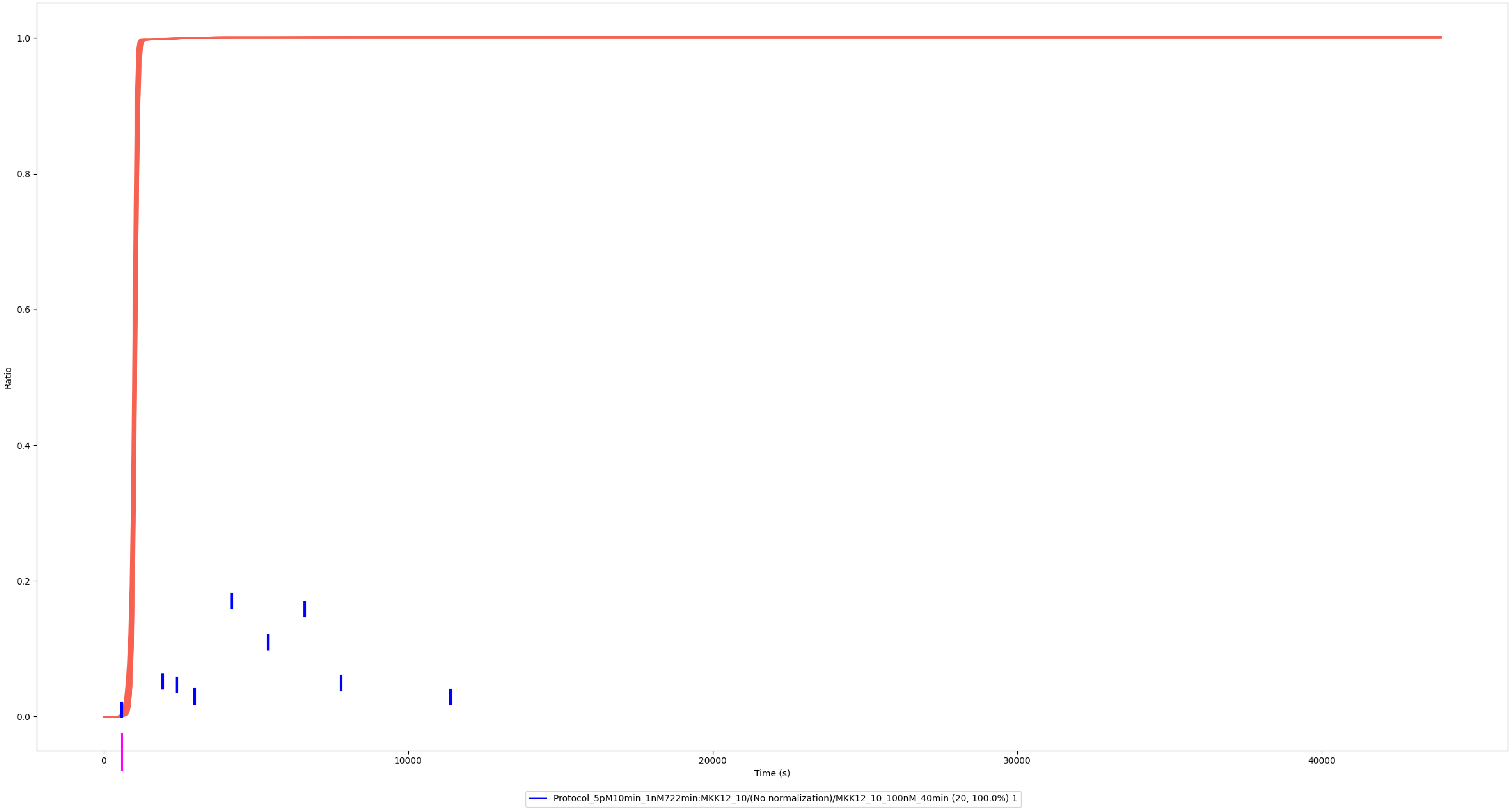

Experiment vs. Simulation: 10 nM LPS, pp-MKK1/2

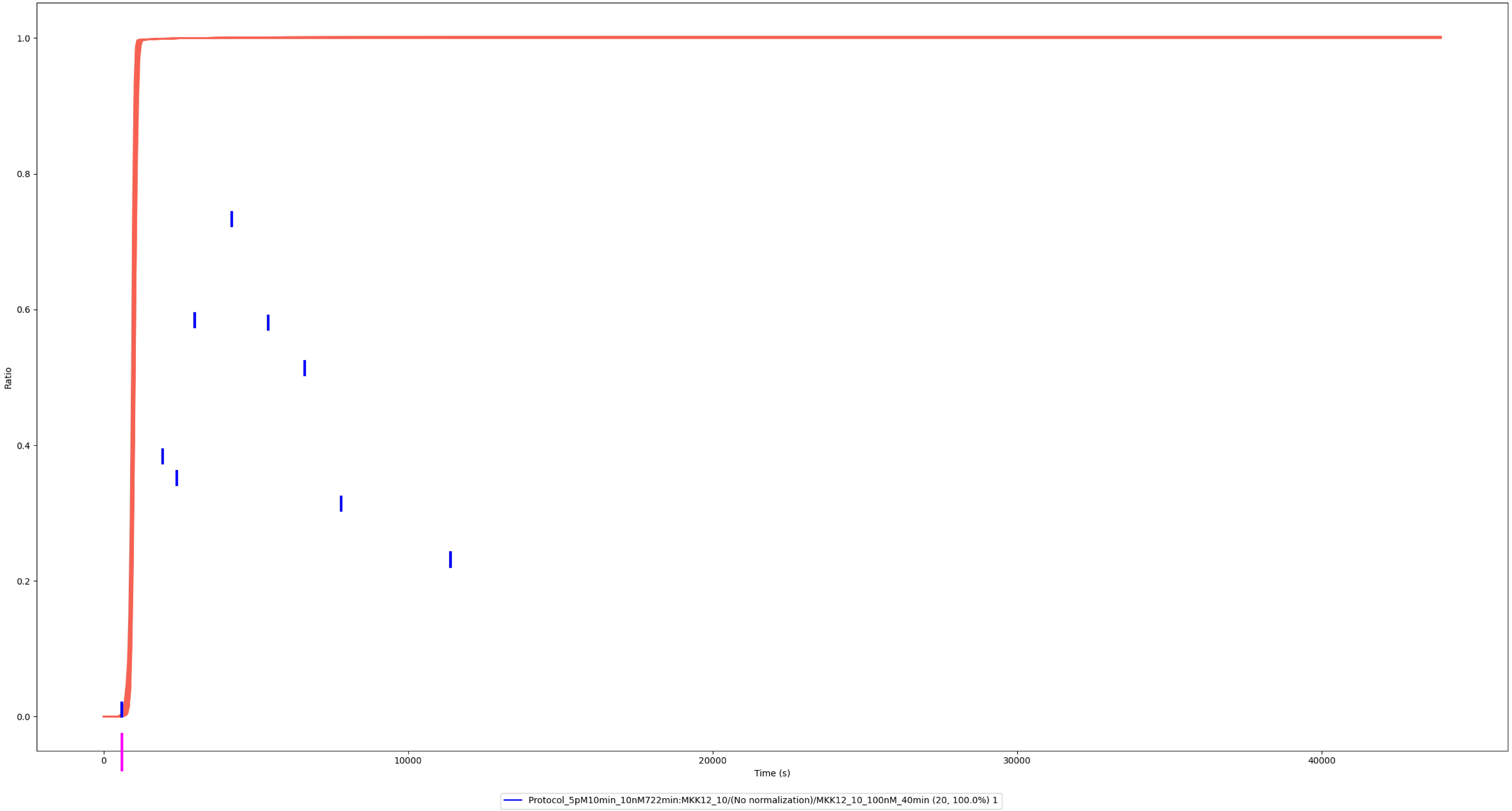

Experiment vs. Simulation: 100 nM LPS, pp-MKK1/2

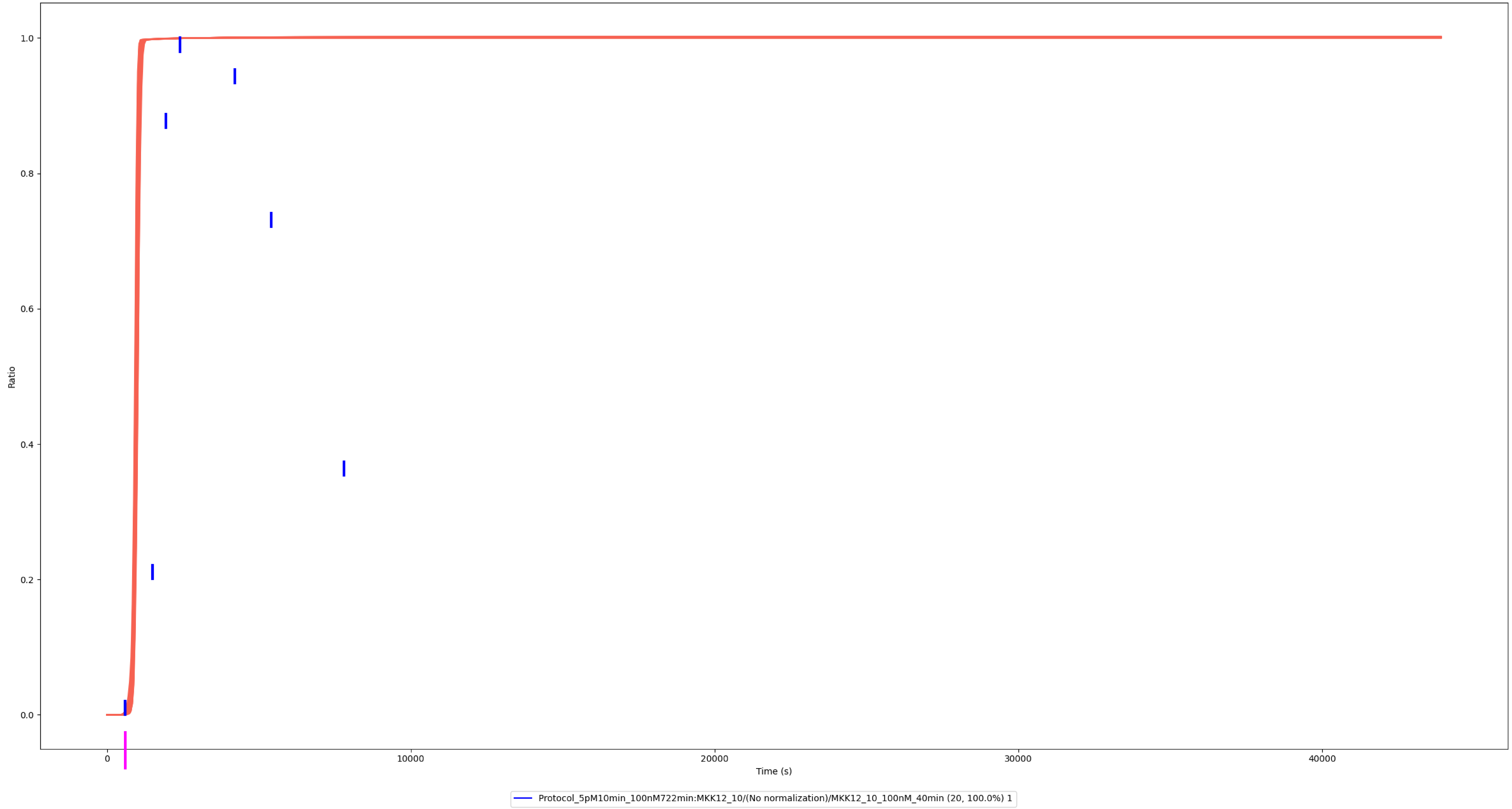

MKK4

Experiment vs. Simulation: 30 nM LPS, pp-MKK4

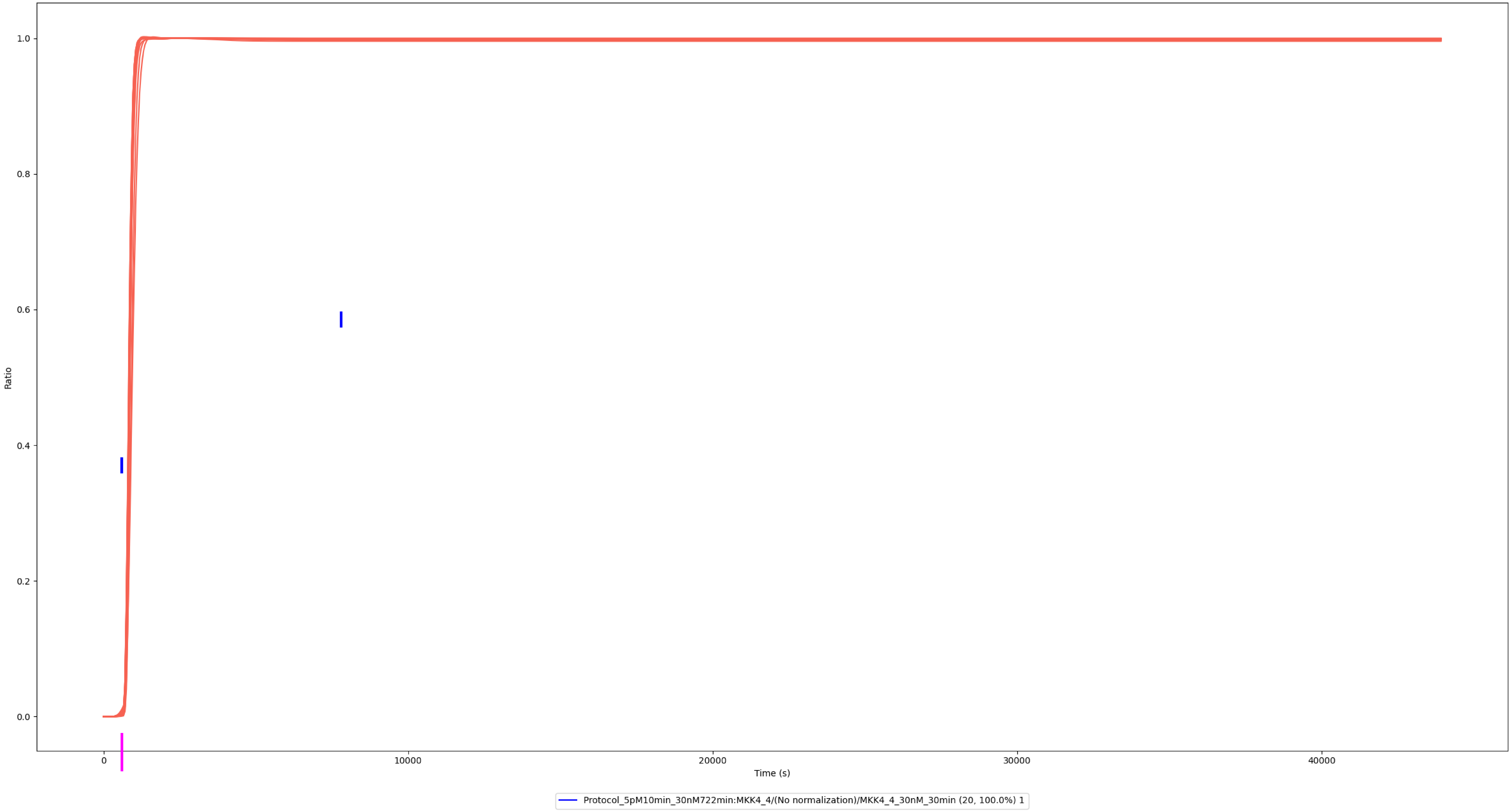

ERK1/2

Experiment vs. Simulation: 0.01 nM LPS, pp-ERK1/2

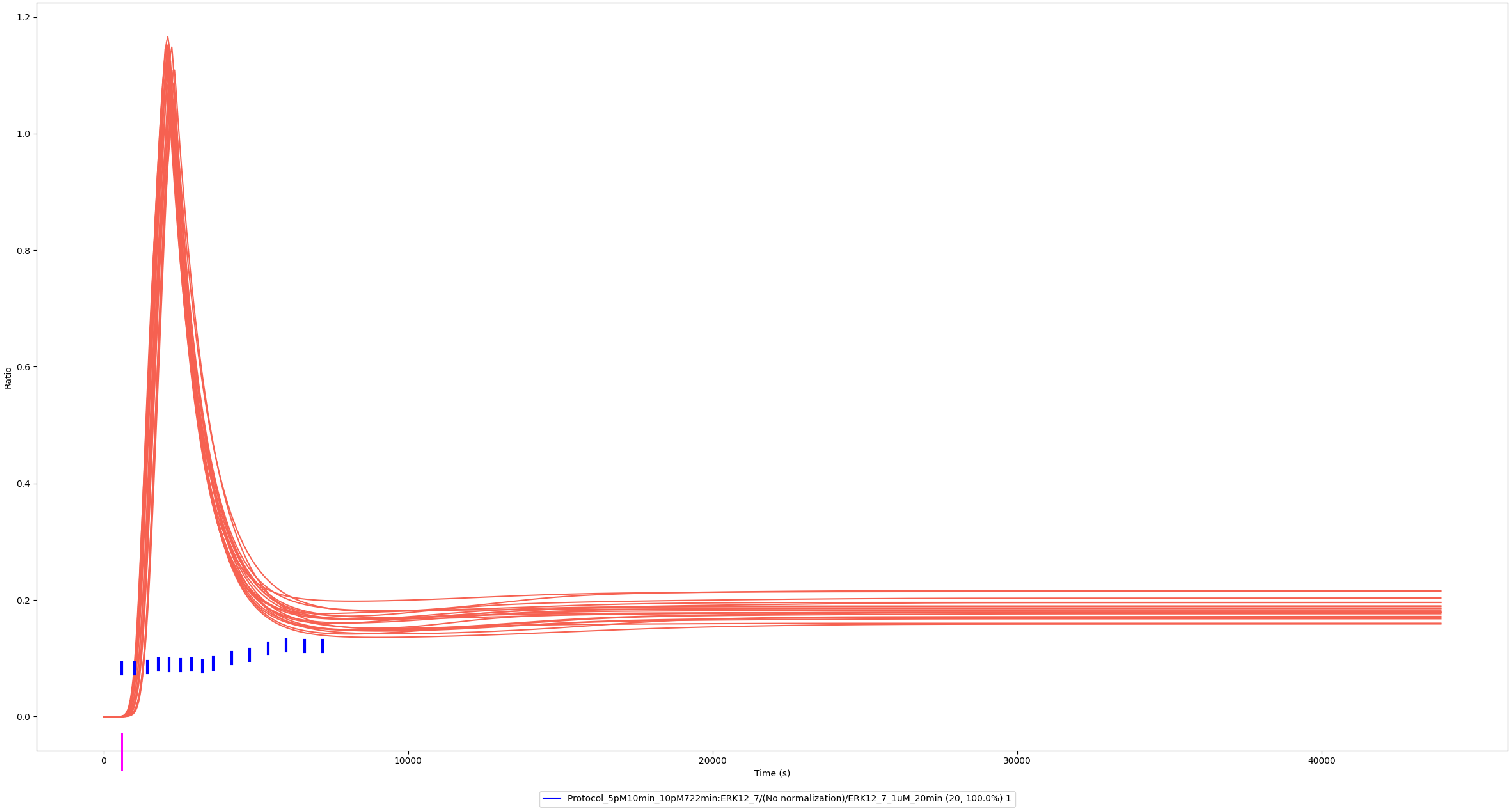

Experiment vs. Simulation: 0.1 nM LPS, pp-ERK1/2

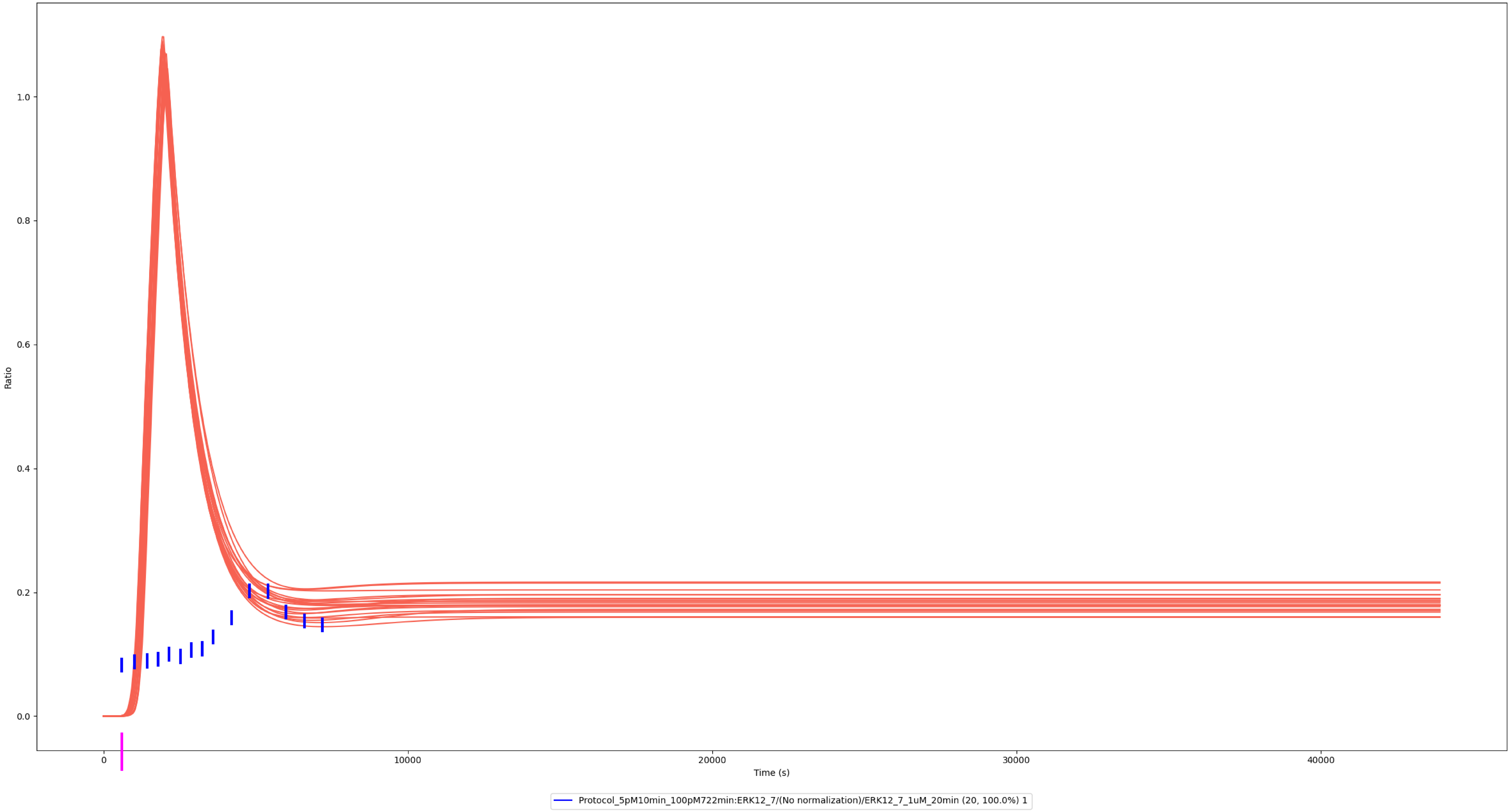

Experiment vs. Simulation: 1 nM LPS, pp-ERK1/2

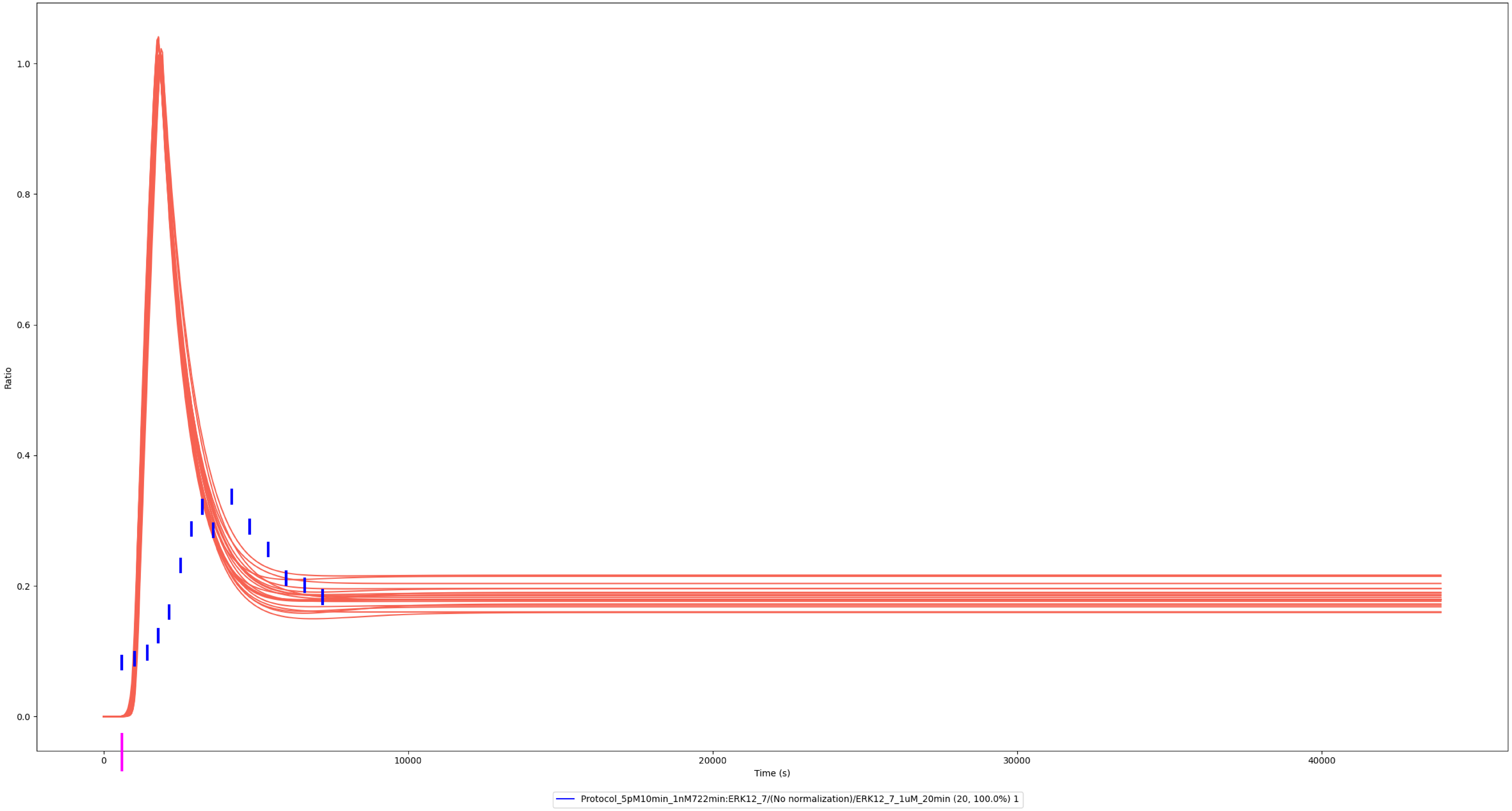

Experiment vs. Simulation: 10 nM LPS, pp-ERK1/2

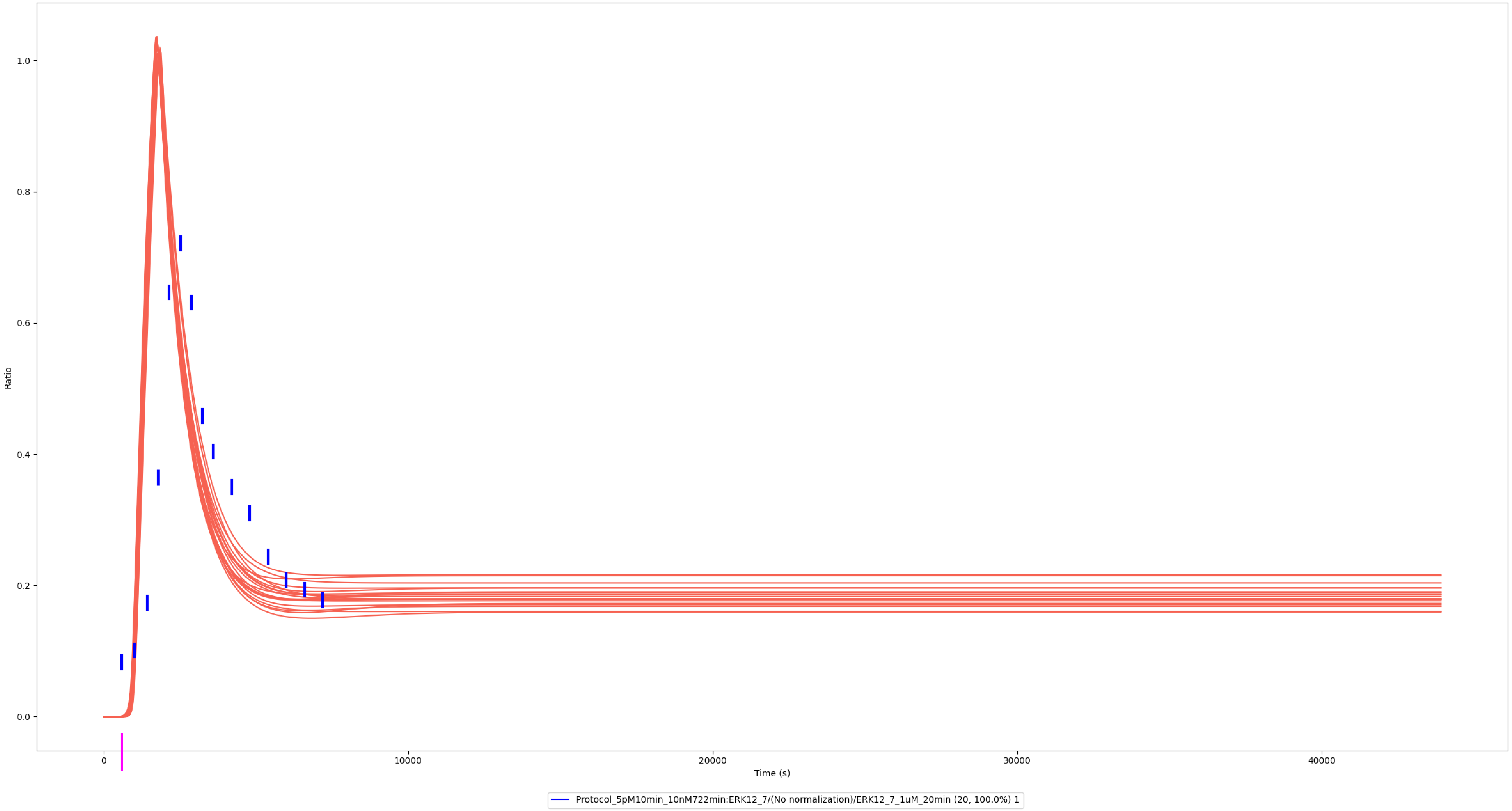

Experiment vs. Simulation: 100 nM LPS, pp-ERK1/2

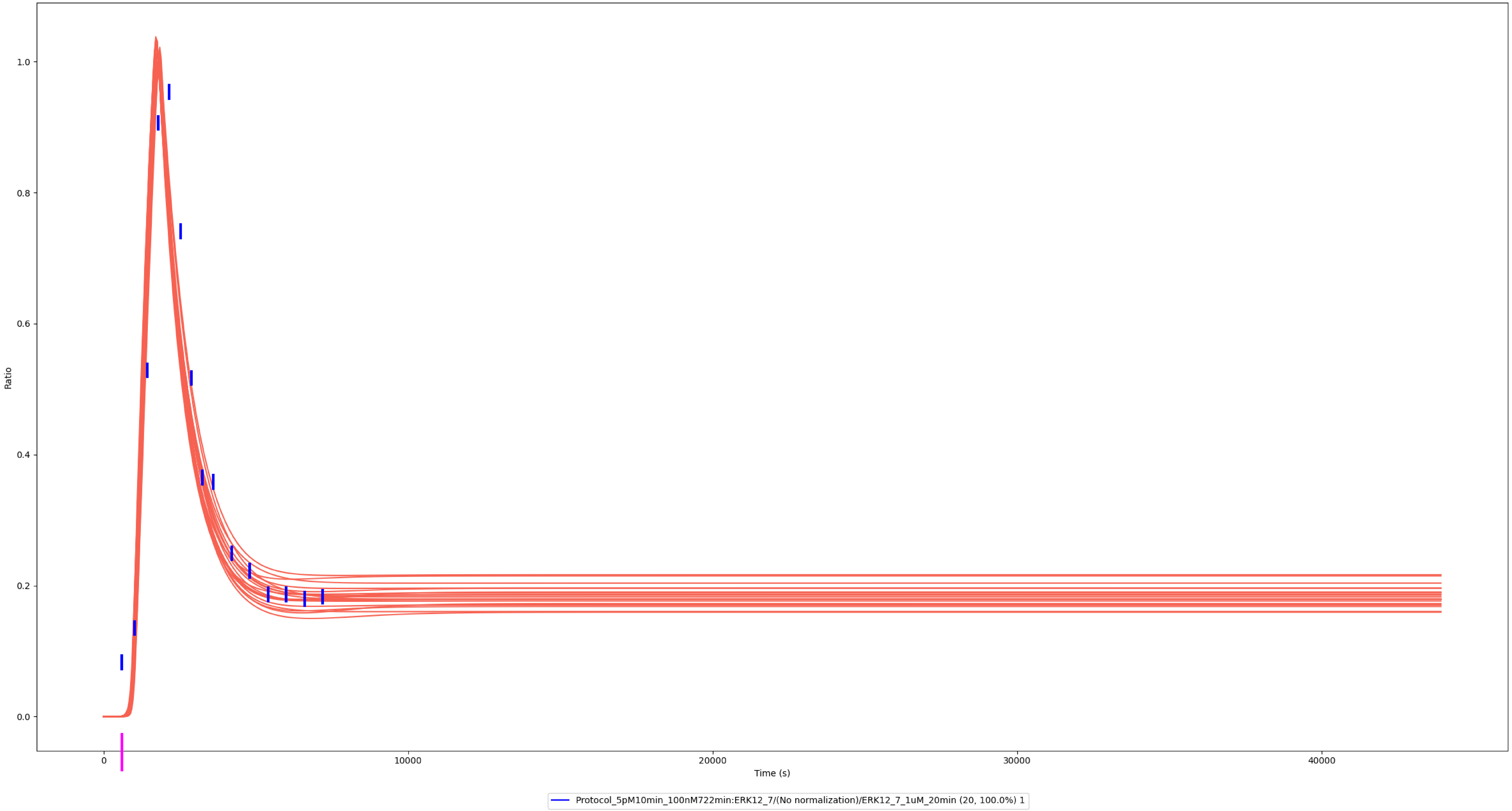

Experiment vs. Simulation: 1,000 nM LPS, pp-ERK1/2

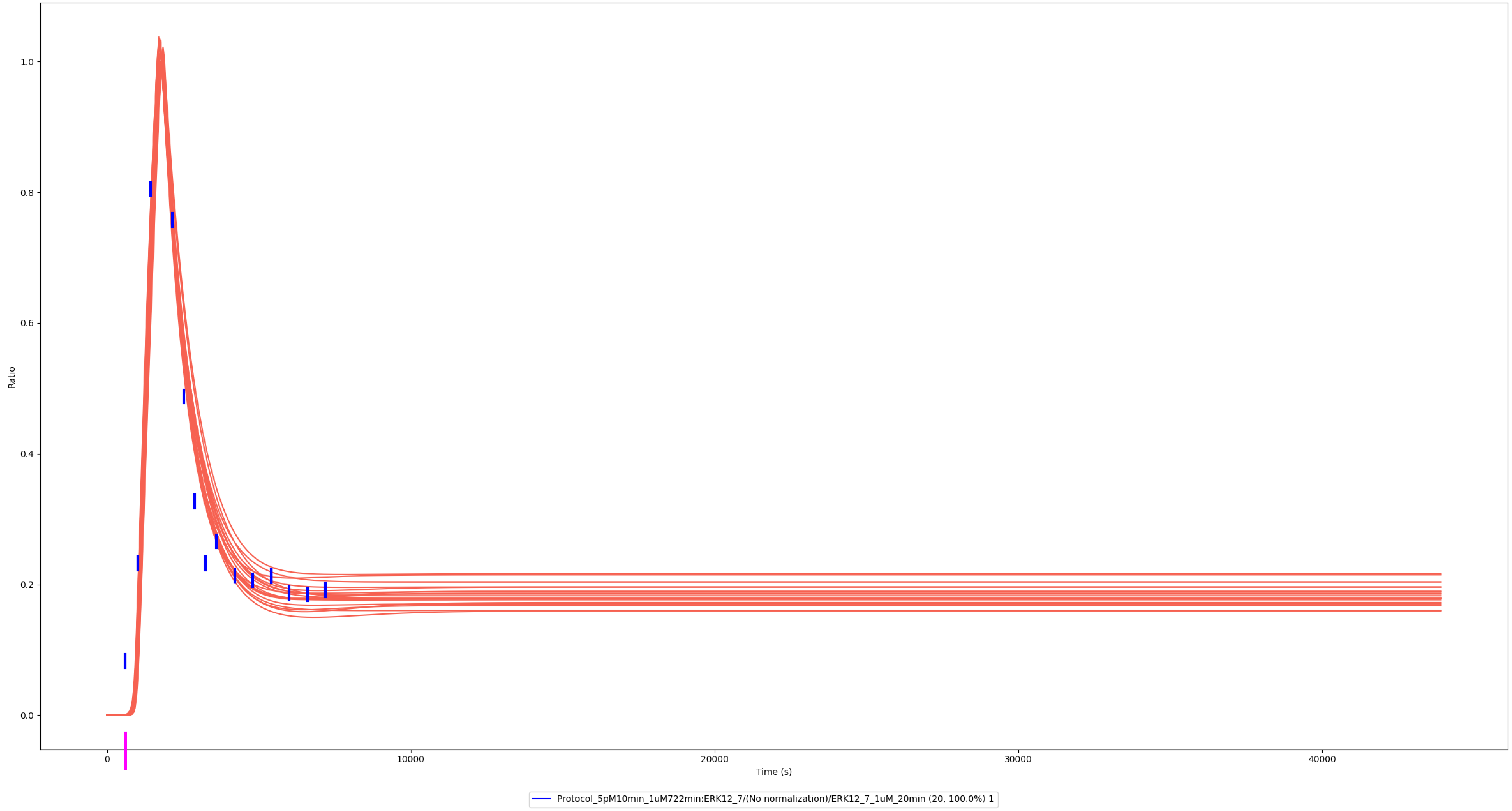

Experiment vs. Simulation: 1 nM LPS, pp-ERK1/2

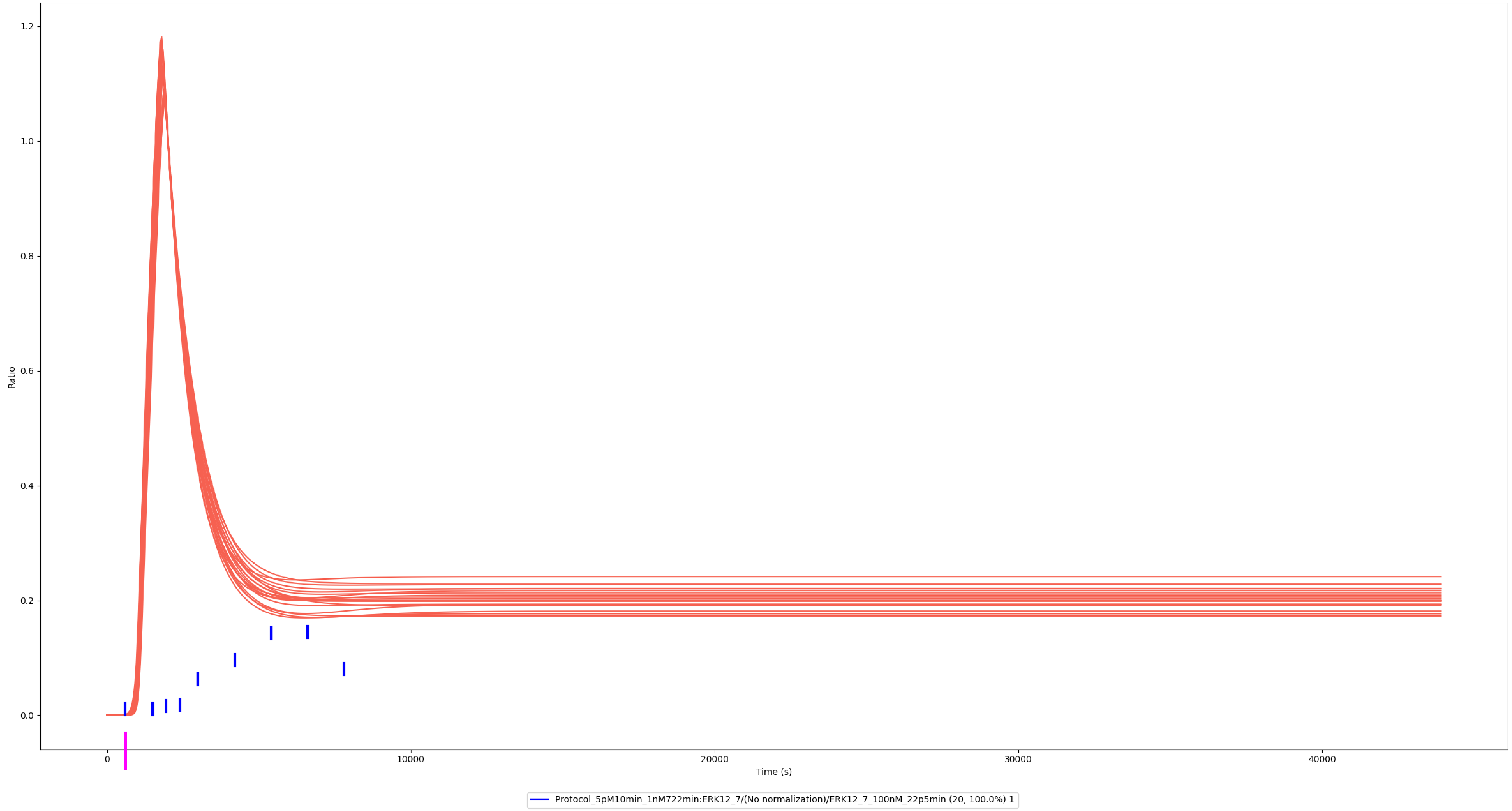

Experiment vs. Simulation: 100 nM LPS, pp-ERK1/2

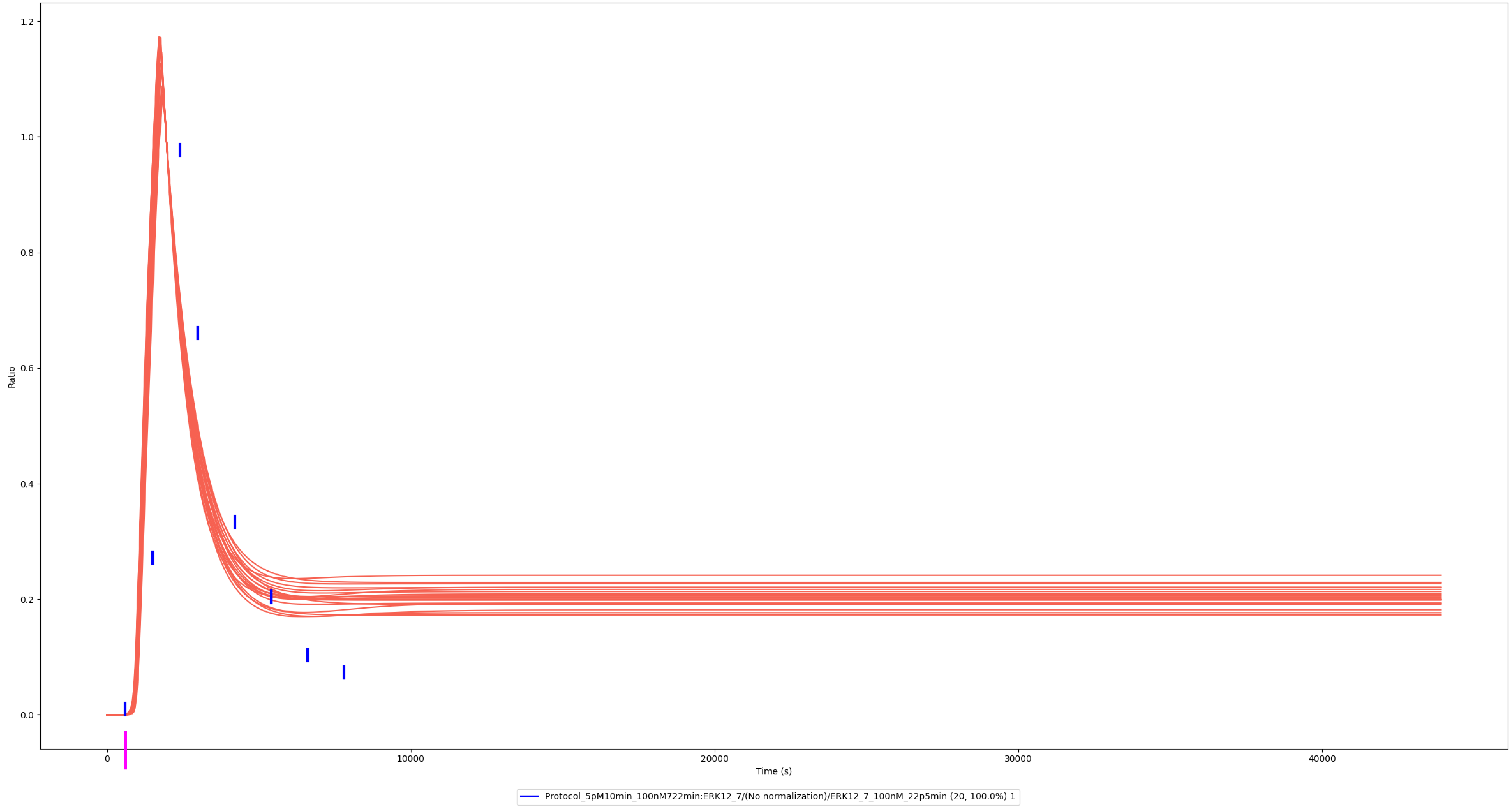

Experiment vs. Simulation: 1 nM LPS, pp-ERK1/2

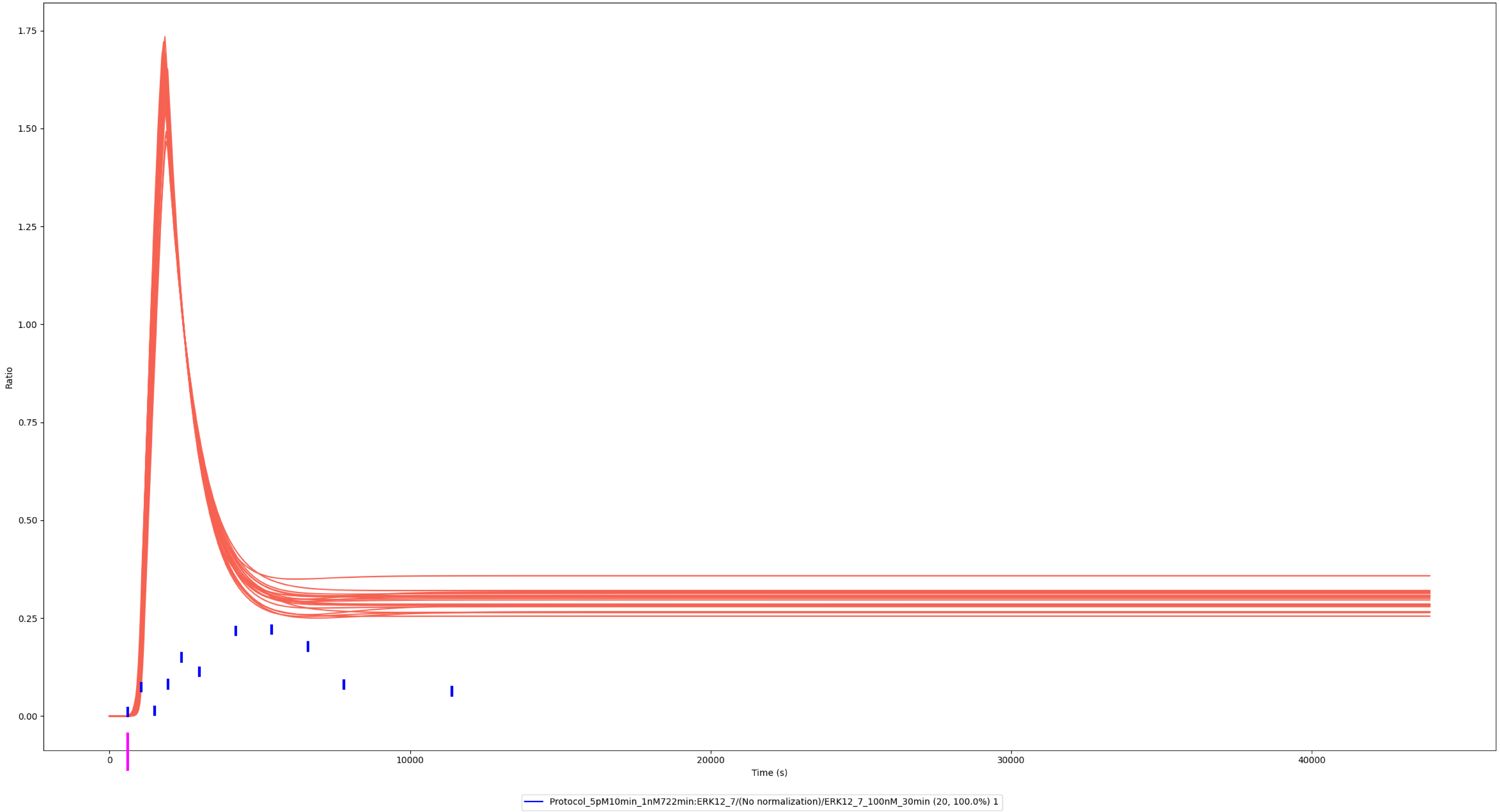

Experiment vs. Simulation: 10 nM LPS, pp-ERK1/2

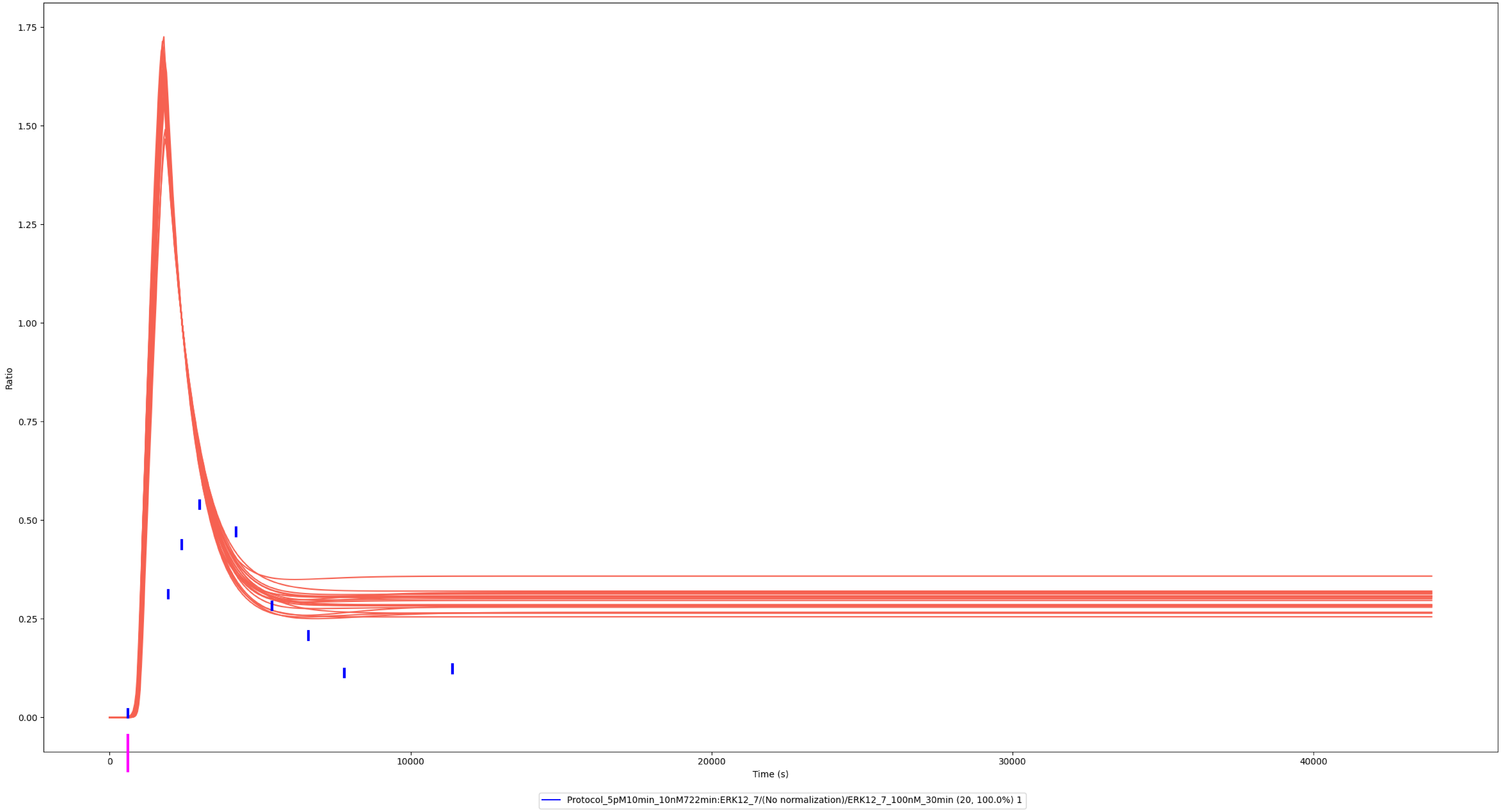

Experiment vs. Simulation: 100 nM LPS, pp-ERK1/2

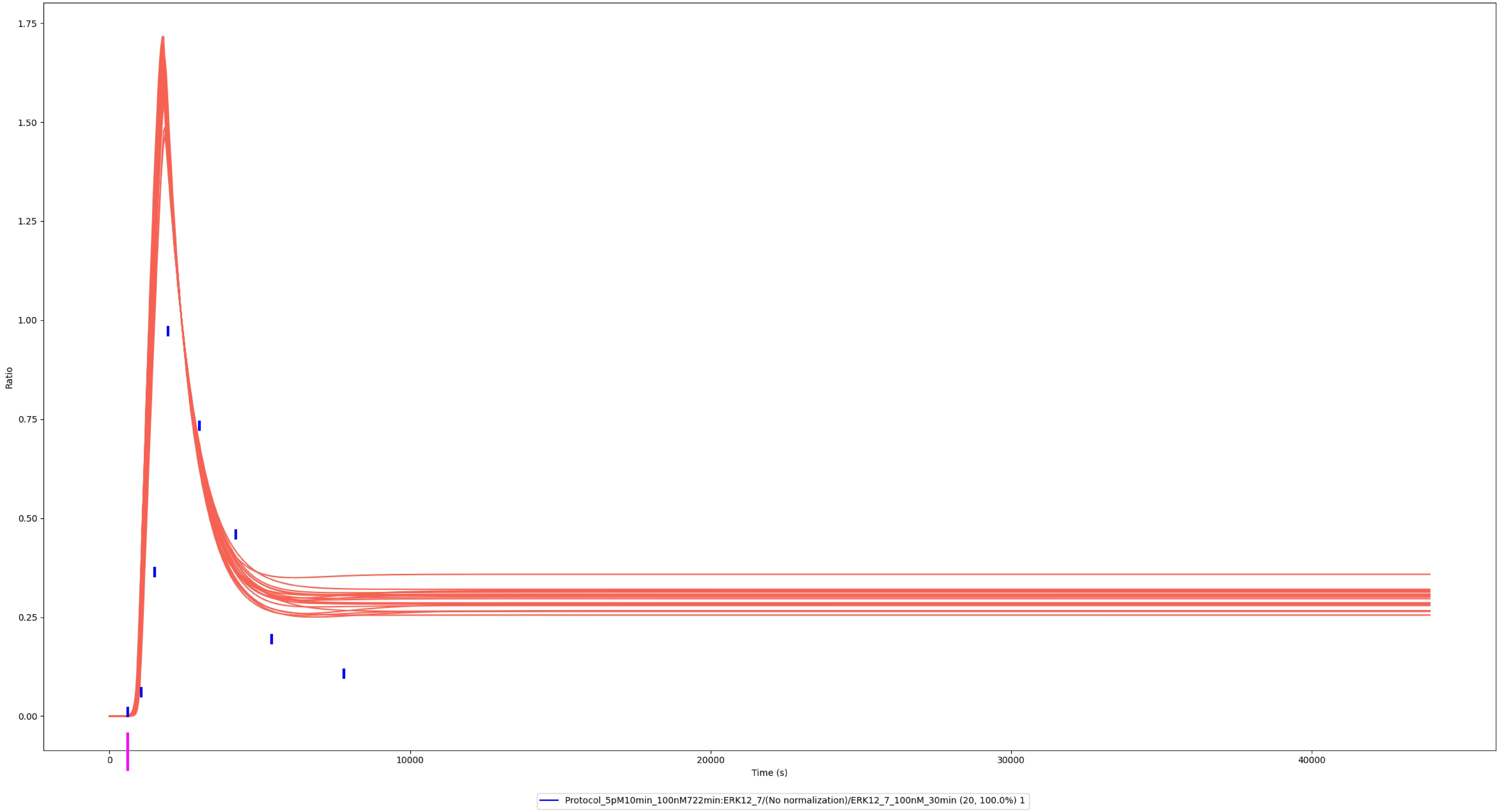

Experiment vs. Simulation: 3 nM LPS, pp-ERK1

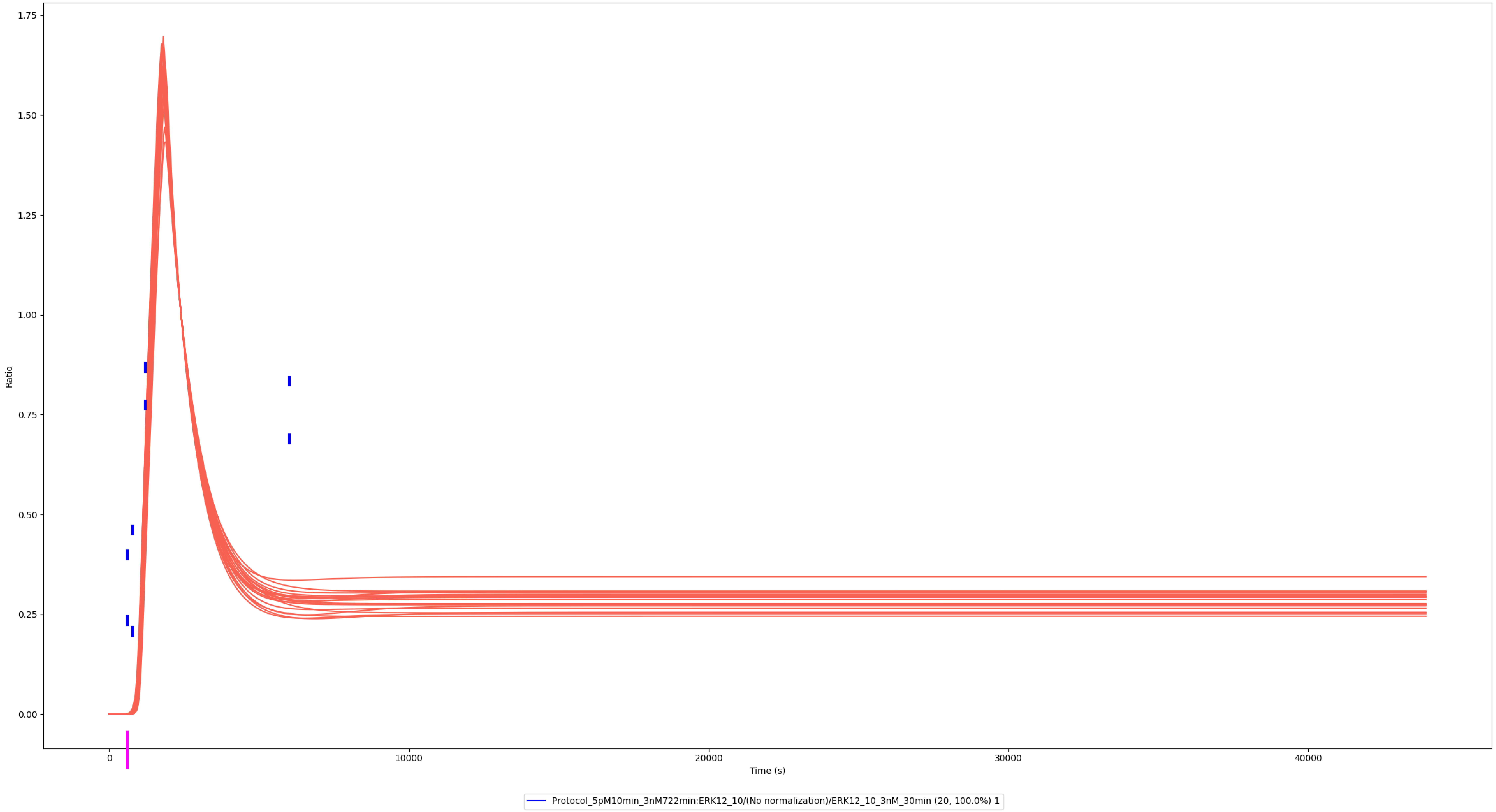

Experiment vs. Simulation: 30 nM LPS, pp-ERK1

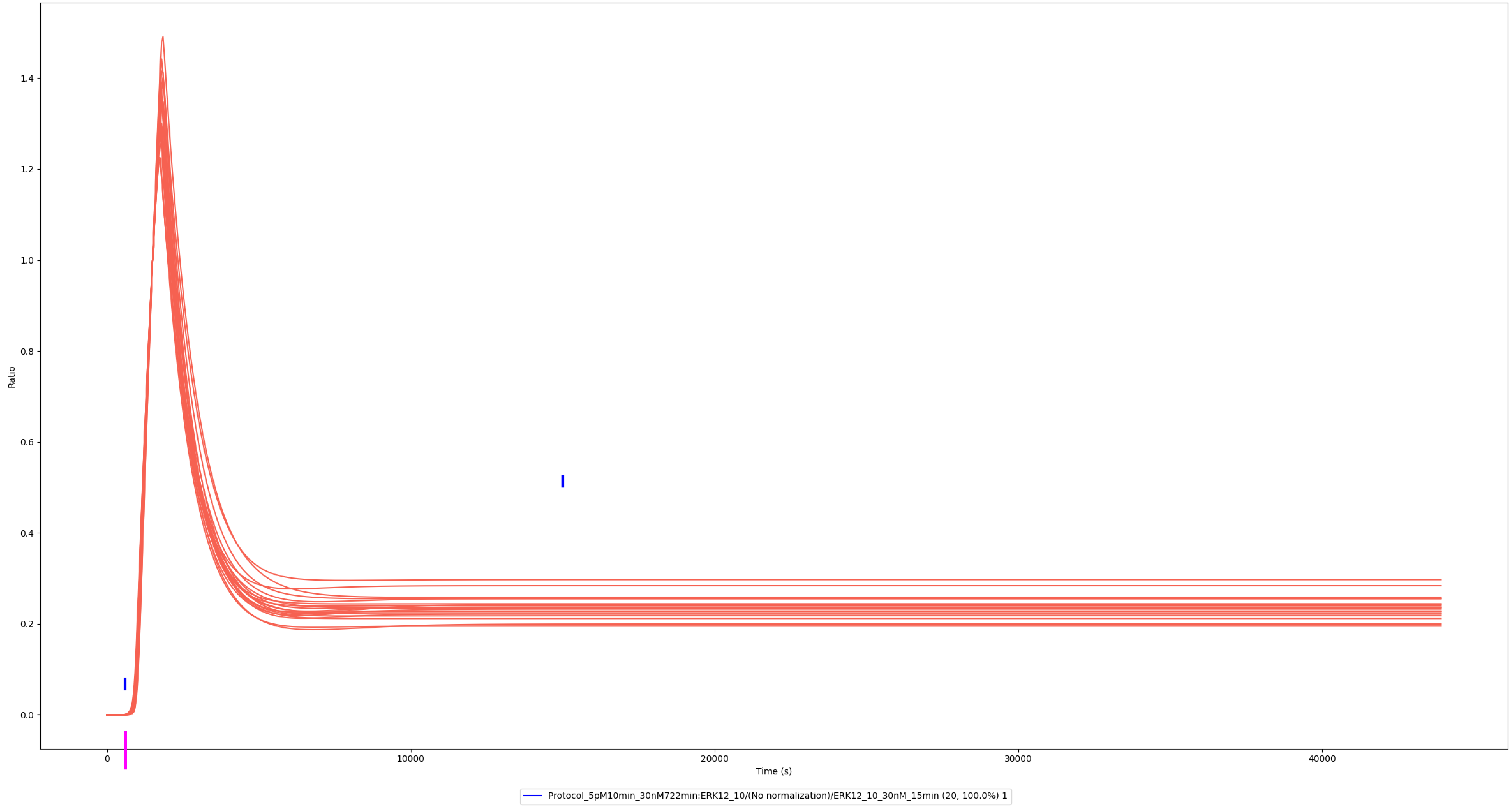

Experiment vs. Simulation: 30 nM LPS, pp-ERK1

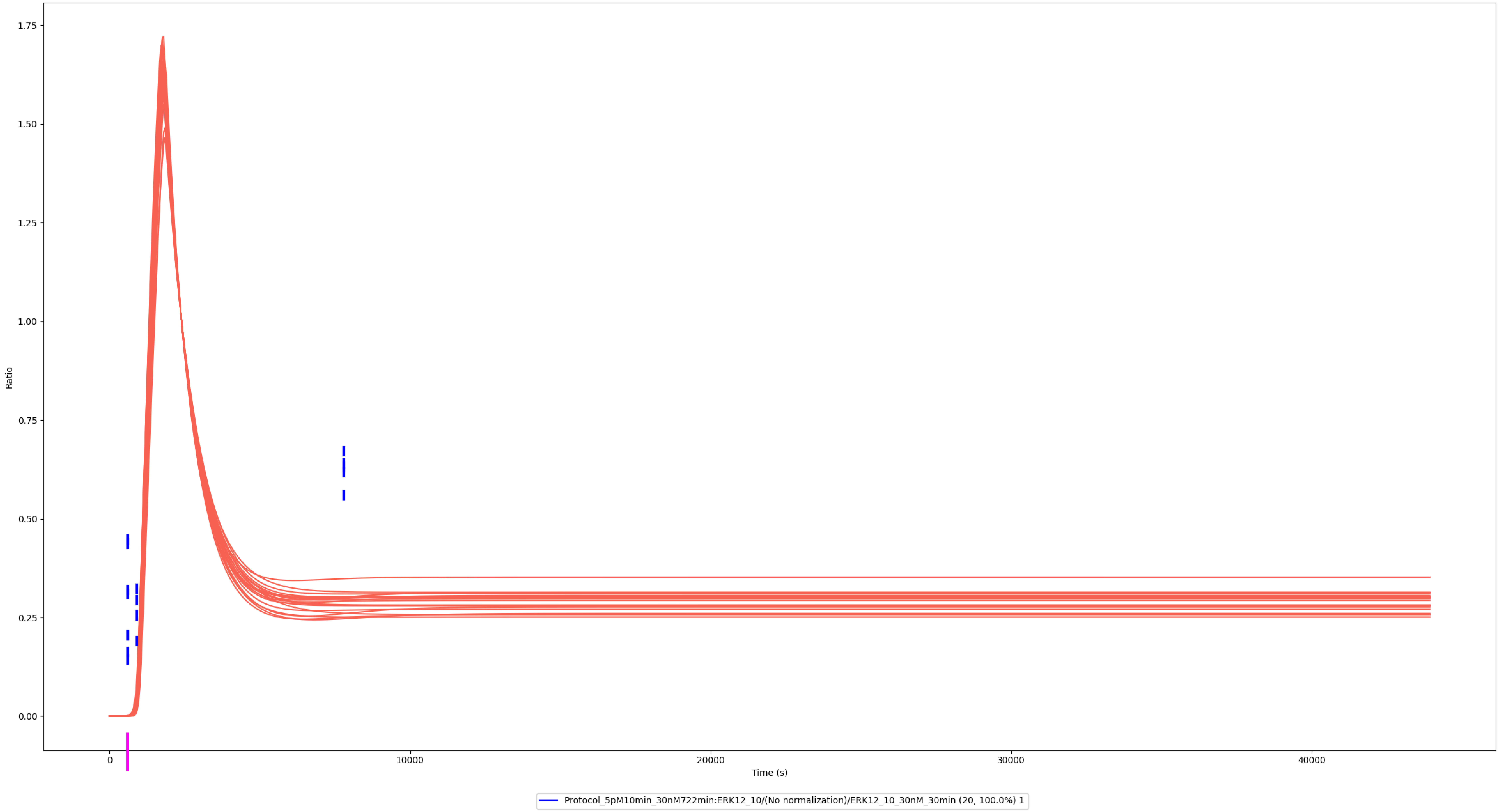

Experiment vs. Simulation: 300 nM LPS, pp-ERK1

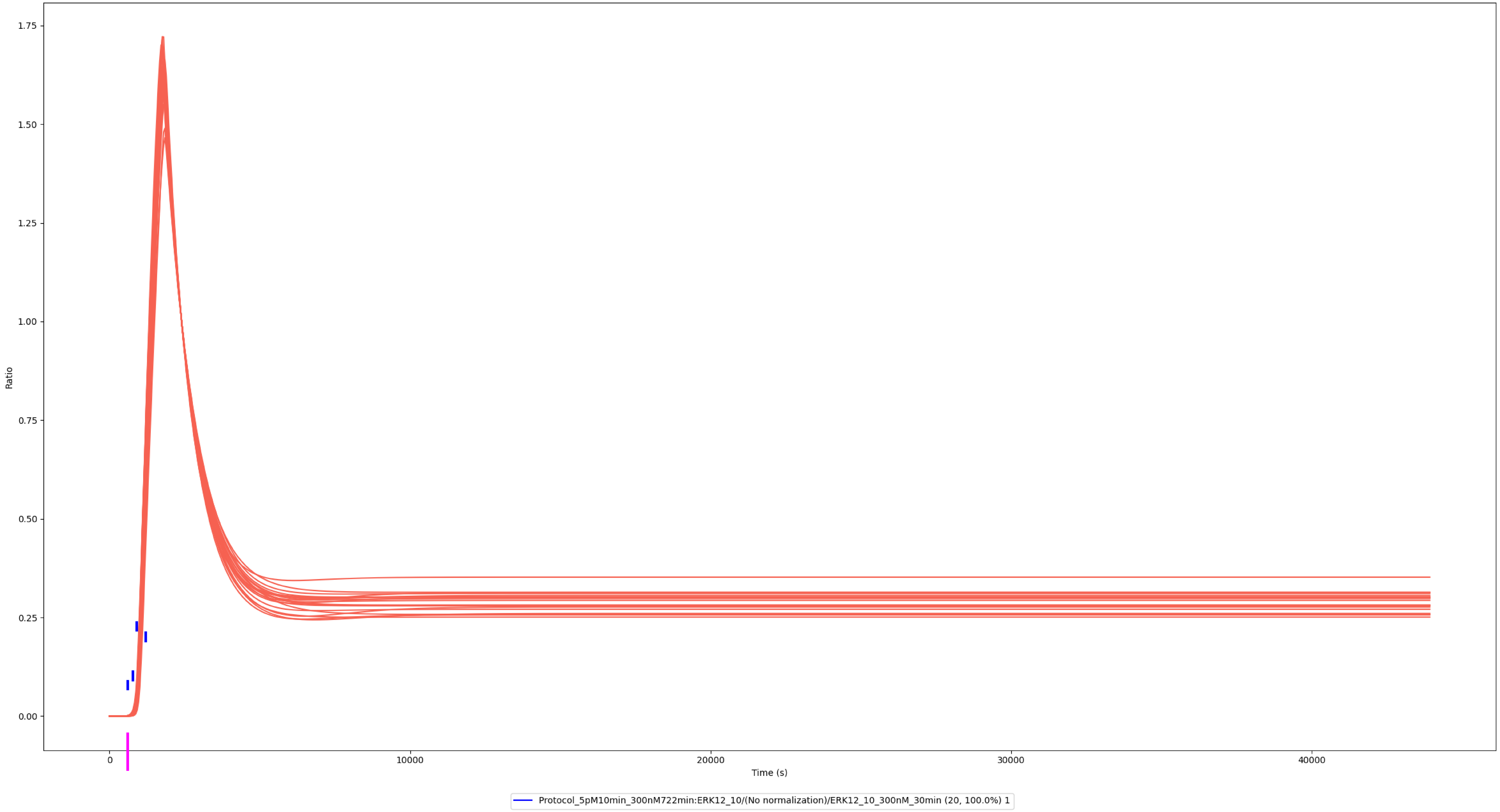

Experiment vs. Simulation: 3 nM LPS, pp-ERK2

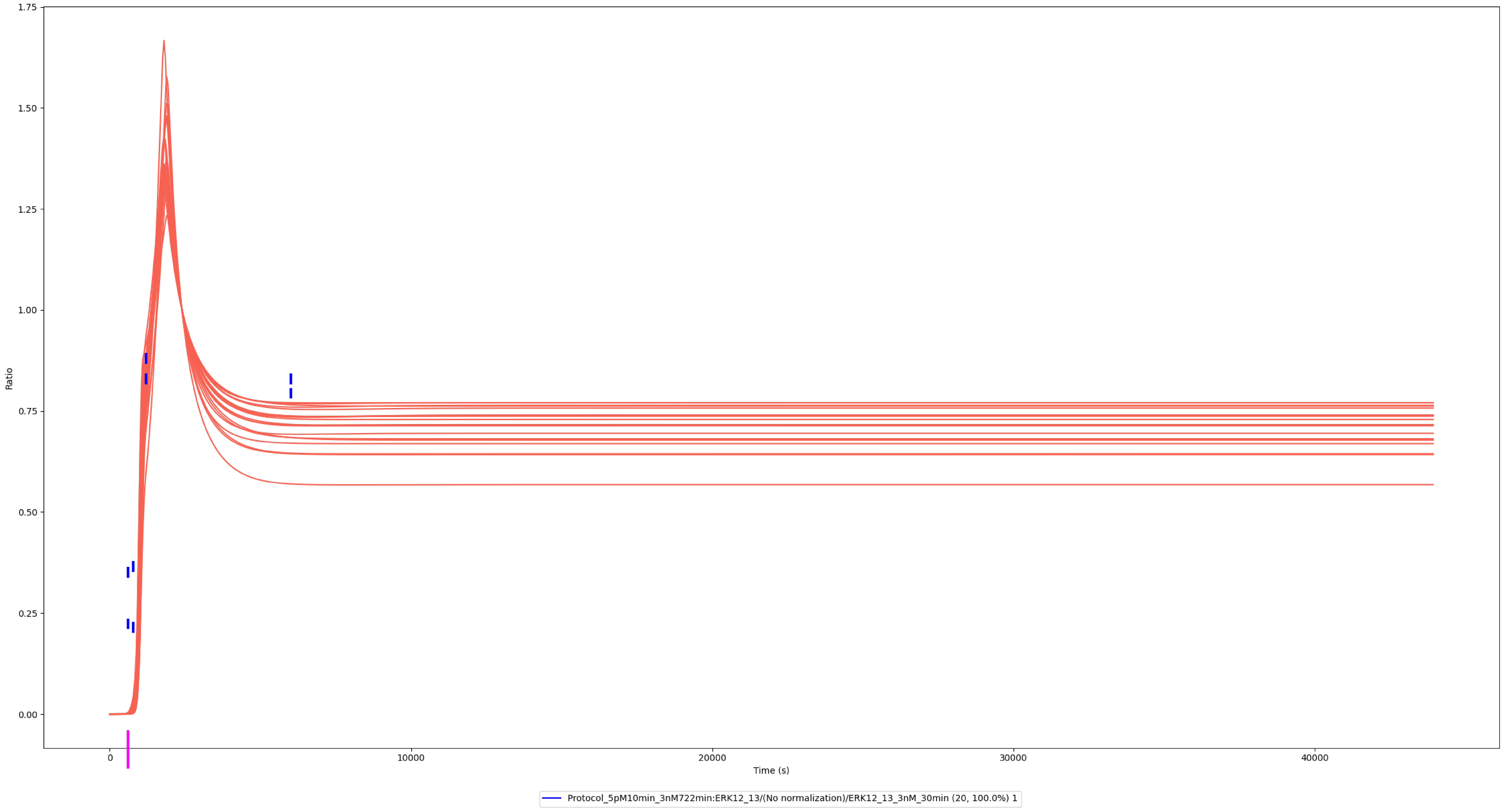

Experiment vs. Simulation: 30 nM LPS, pp-ERK2

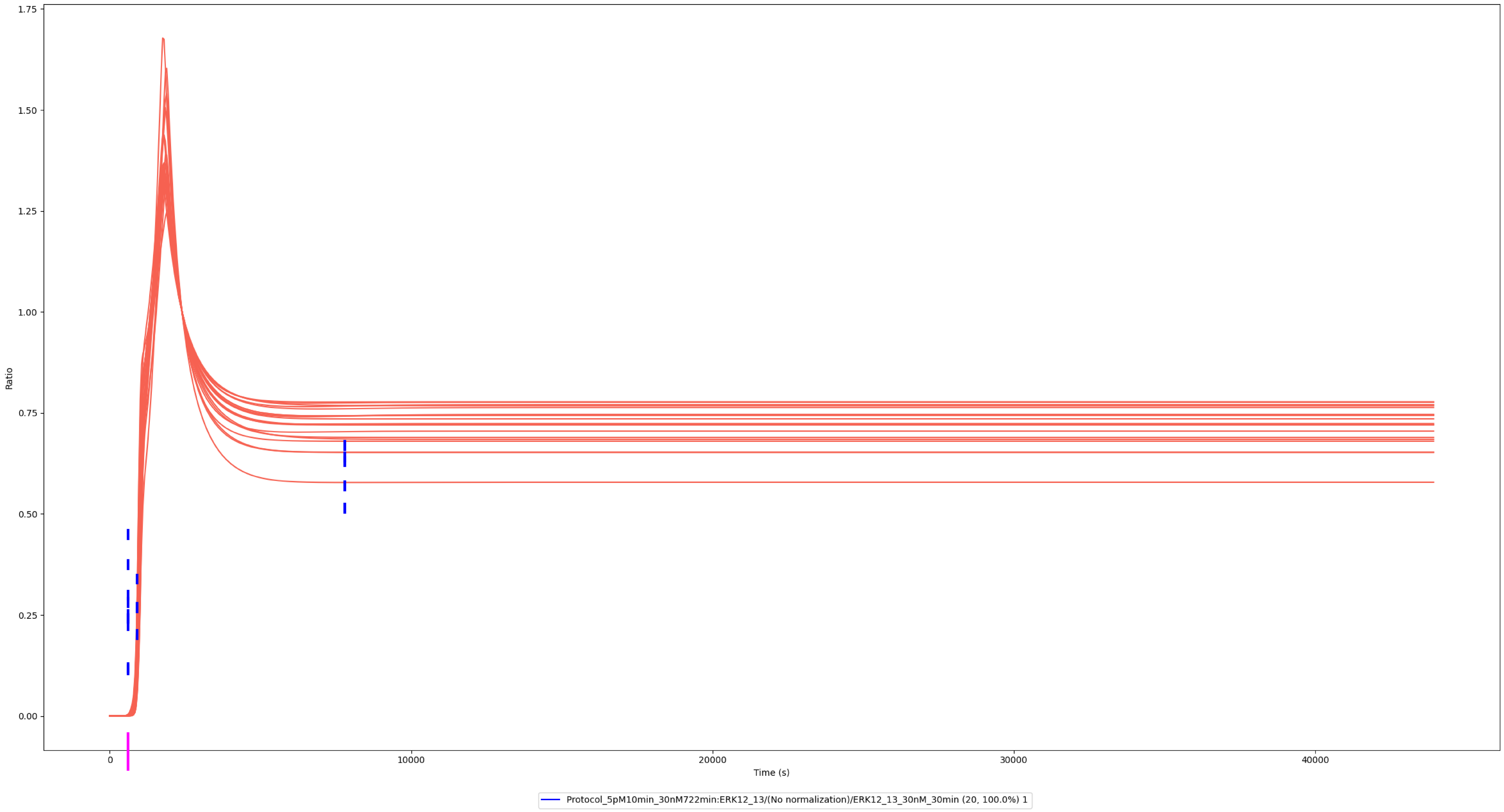

JNK1/2/3

Experiment vs. Simulation: 3 nM LPS, pp-JNK1/3

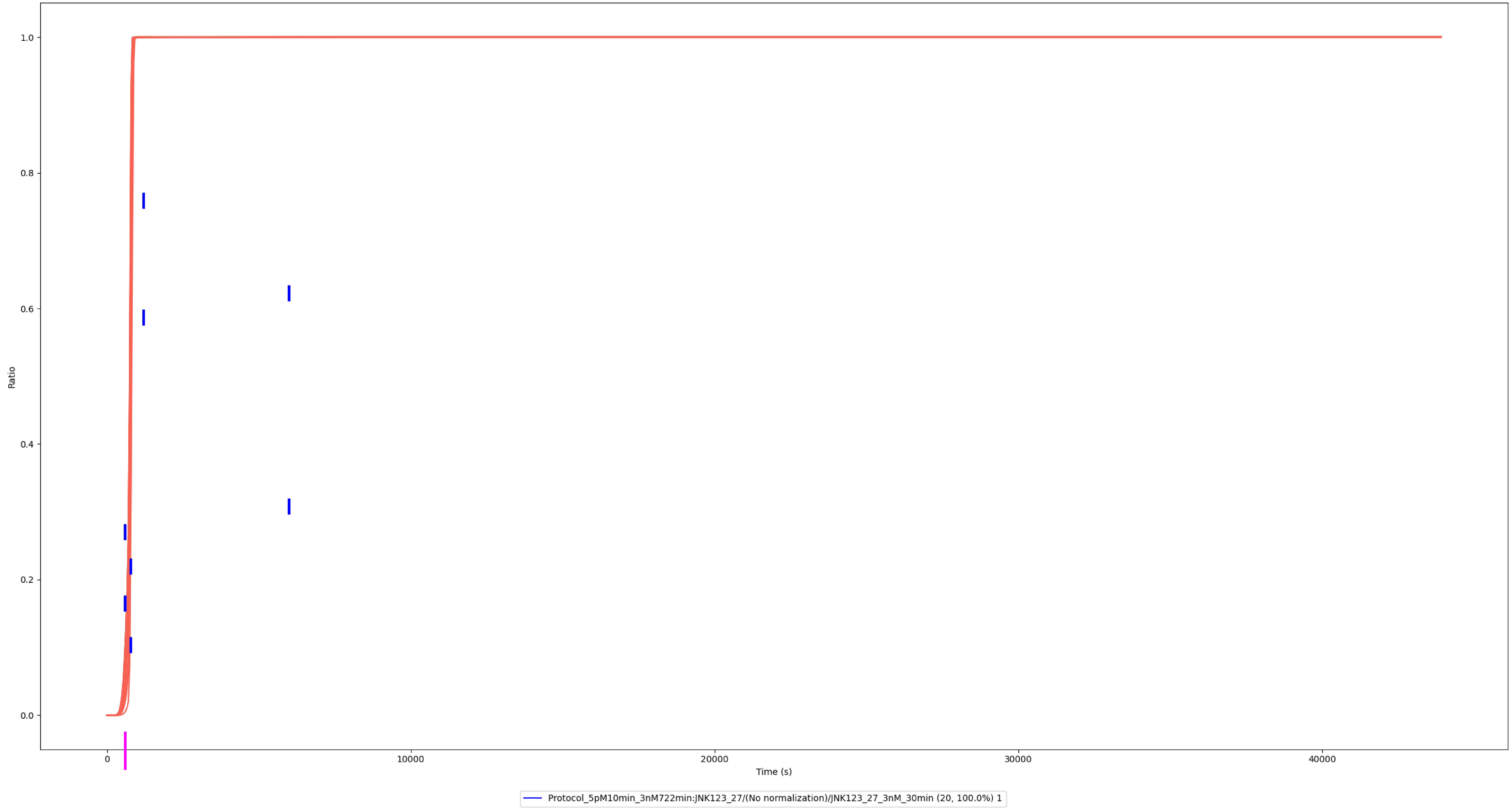

Experiment vs. Simulation: 30 nM LPS, pp-JNK1/3

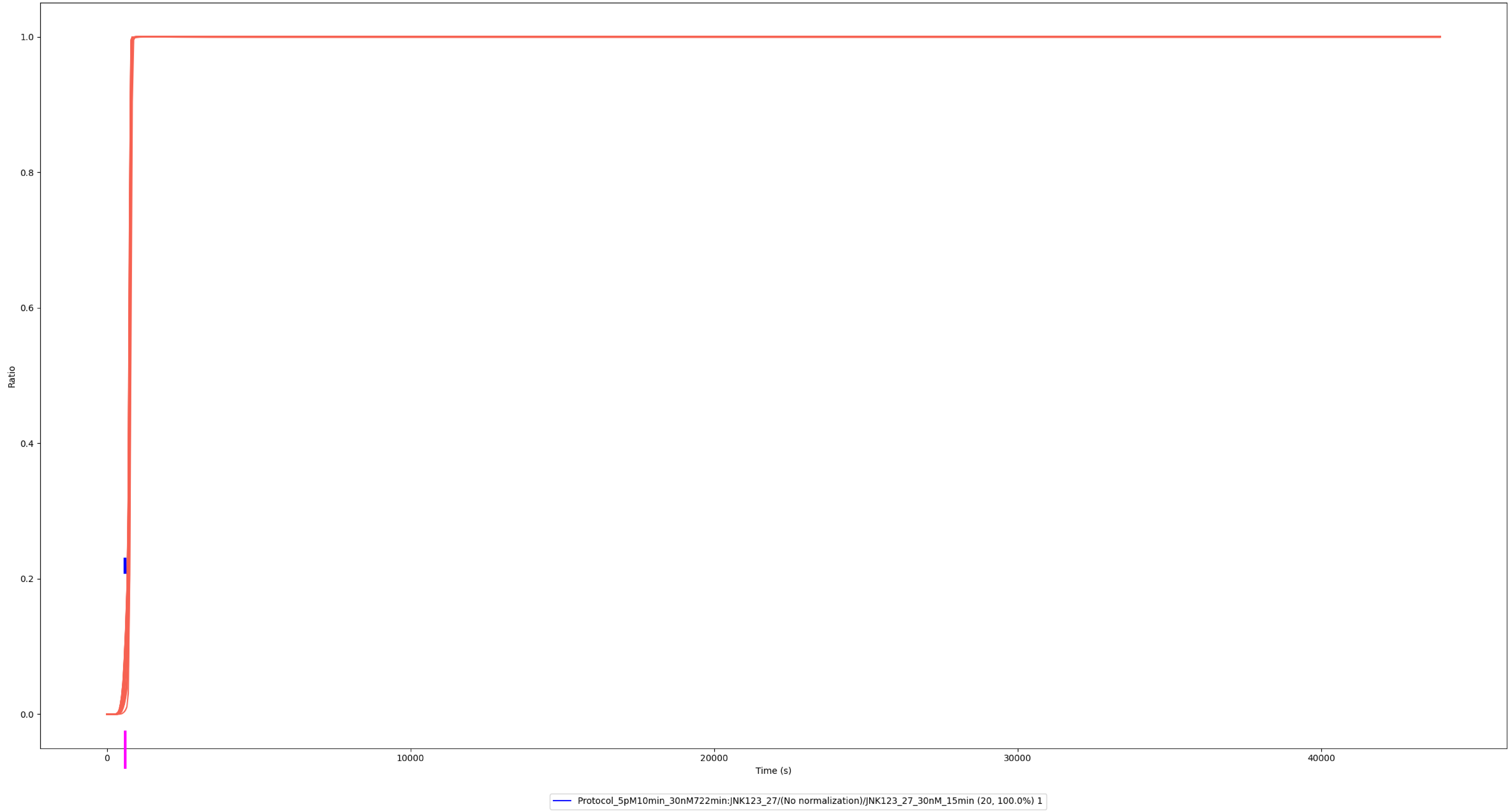

Experiment vs. Simulation: 30 nM LPS, pp-JNK1/3

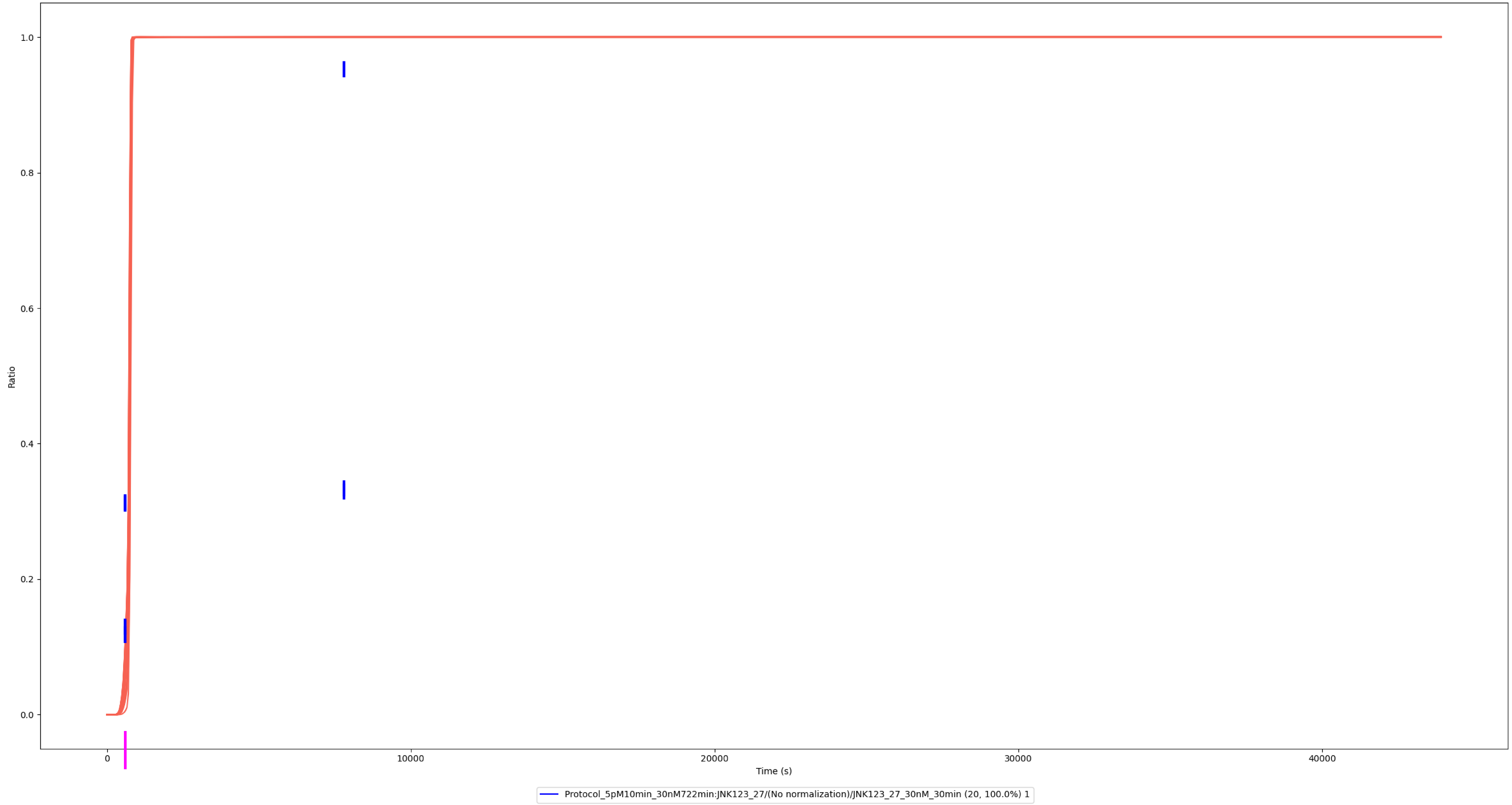

Experiment vs. Simulation: 30 nM LPS, pp-JNK2

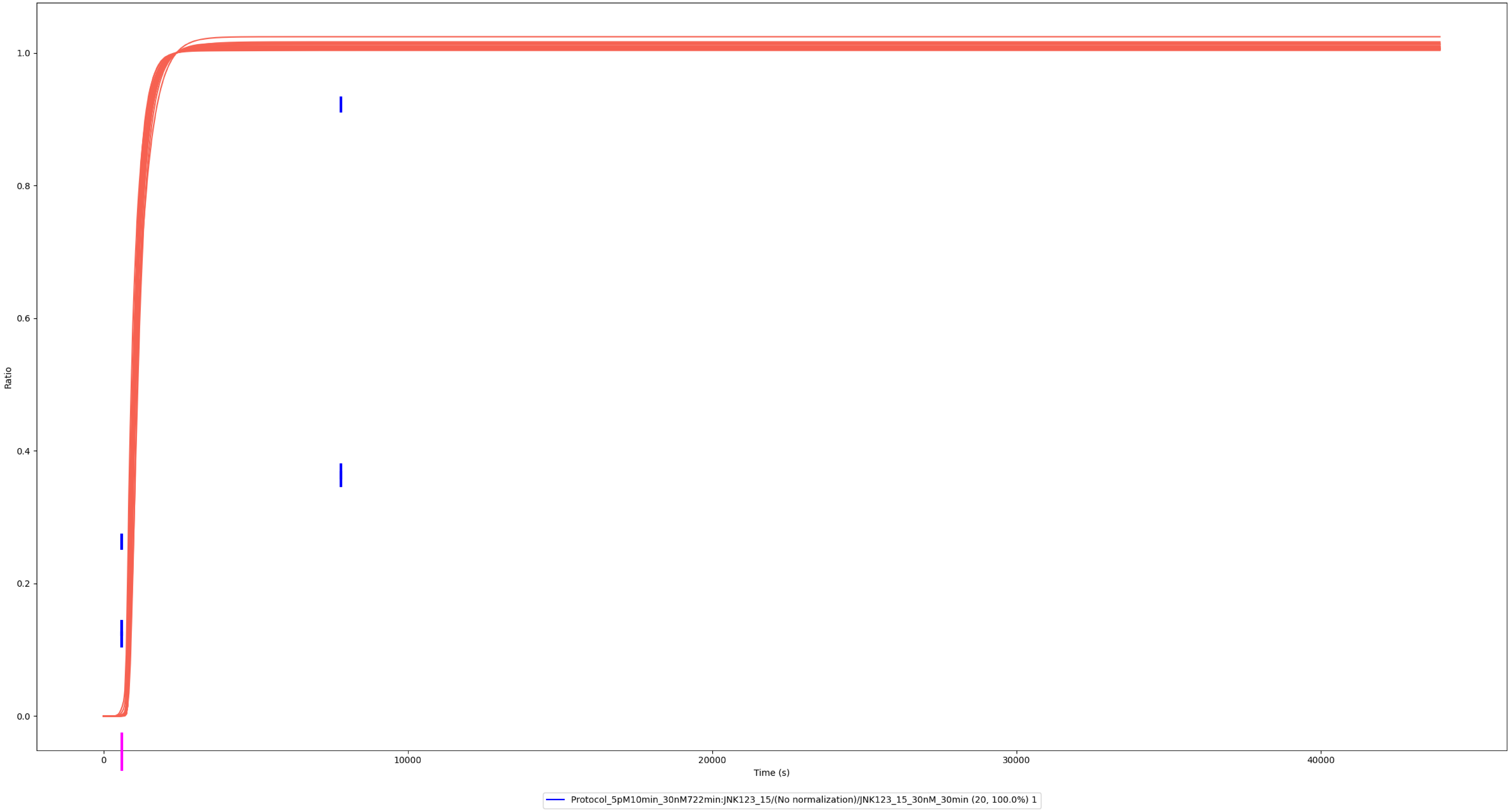

Experiment vs. Simulation: 30 nM LPS, pp-JNK2

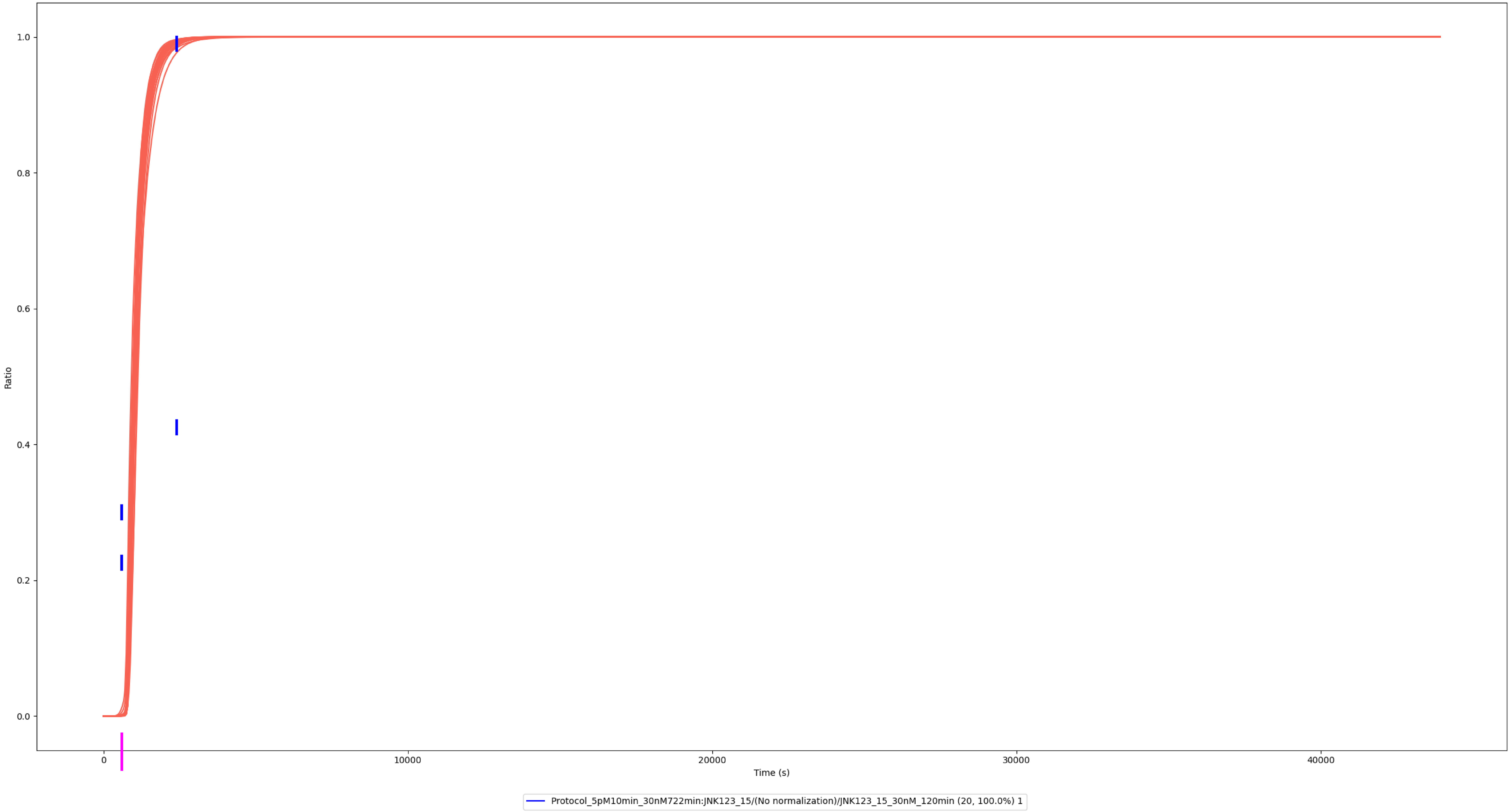

p38α/β/γ/δ

Experiment vs. Simulation: 0.01 nM LPS, pp-p38α/β/γ/δ

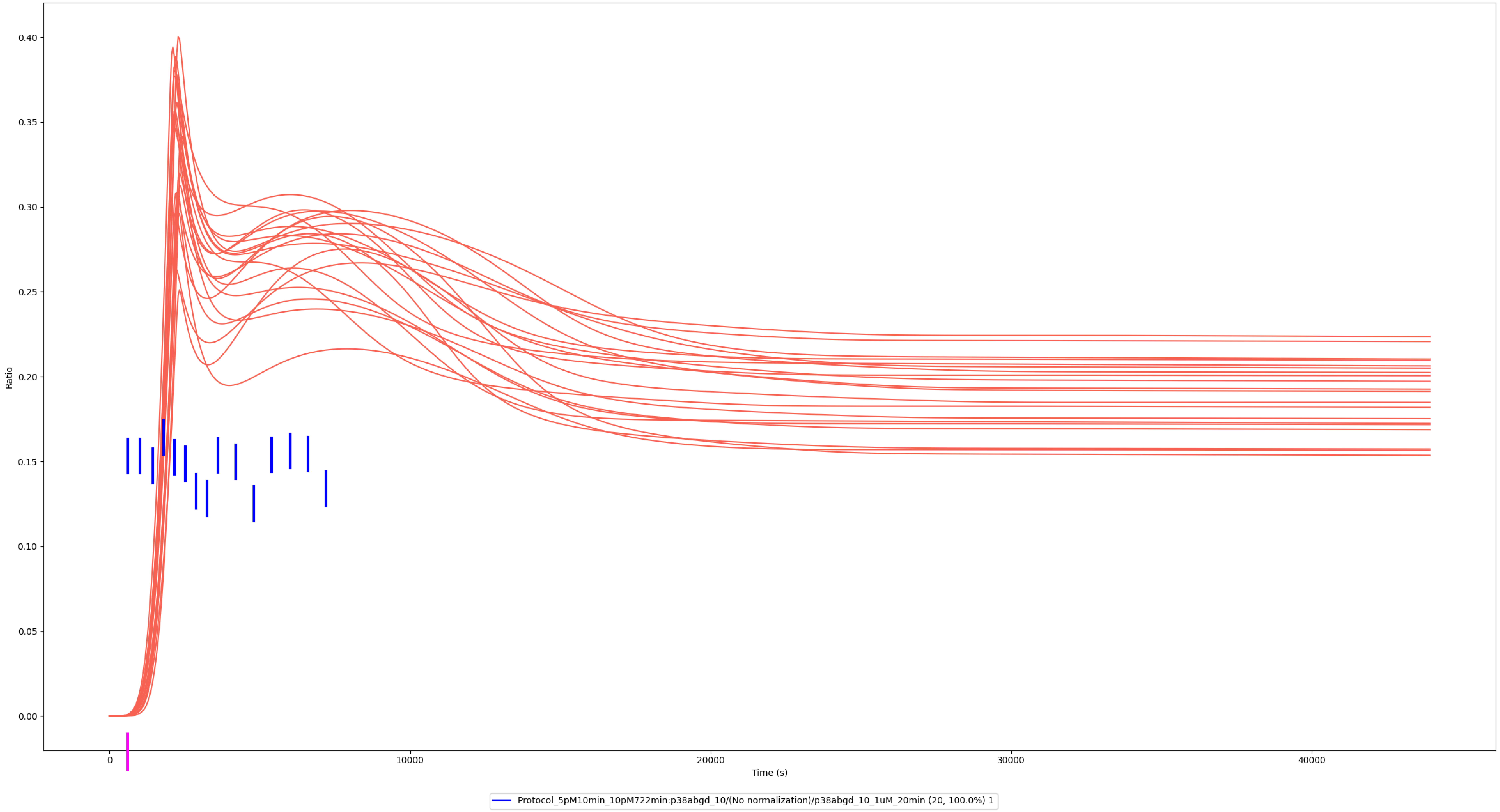

Experiment vs. Simulation: 0.1 nM LPS, pp-p38α/β/γ/δ

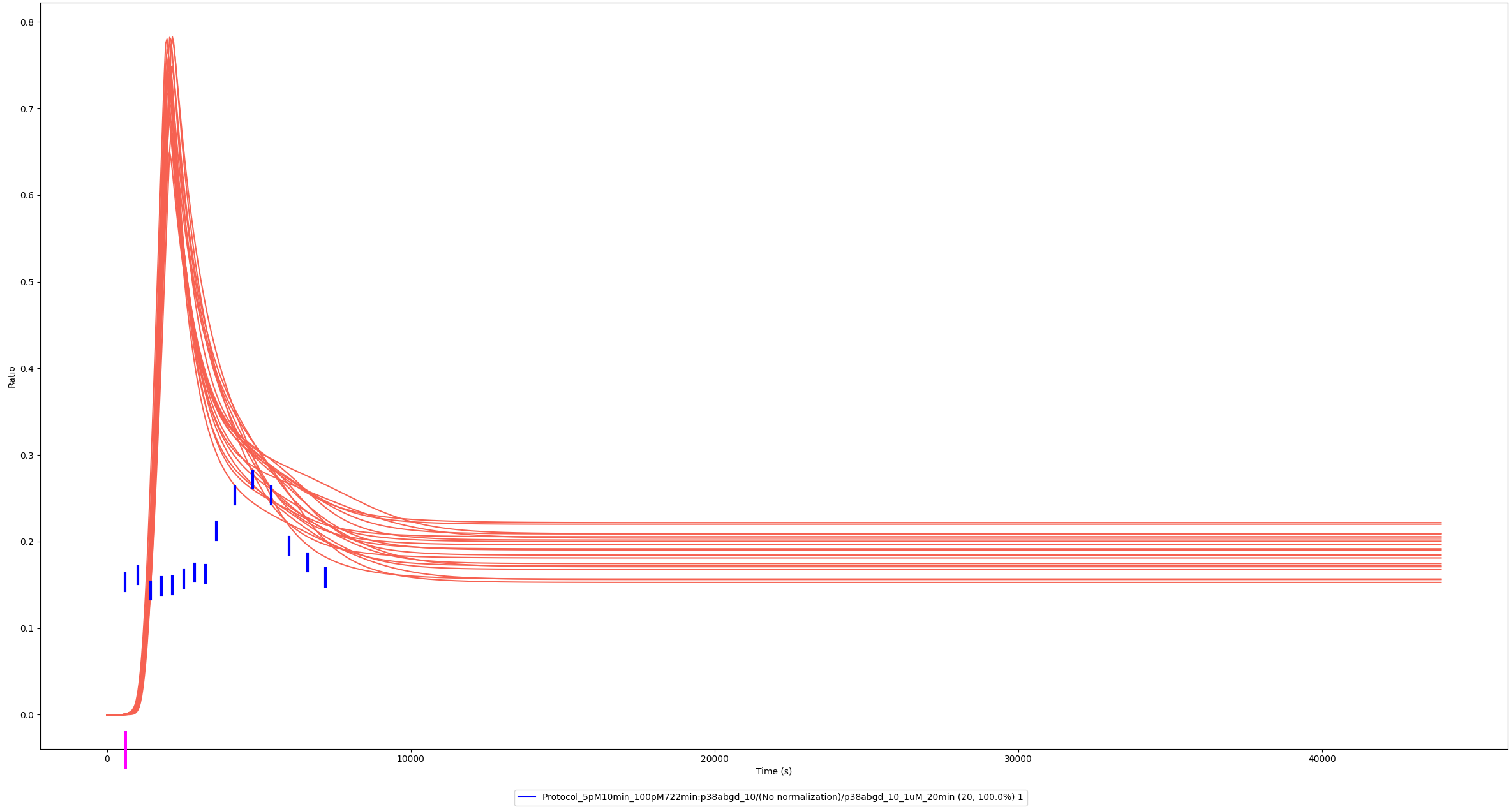

Experiment vs. Simulation: 1 nM LPS, pp-p38α/β/γ/δ

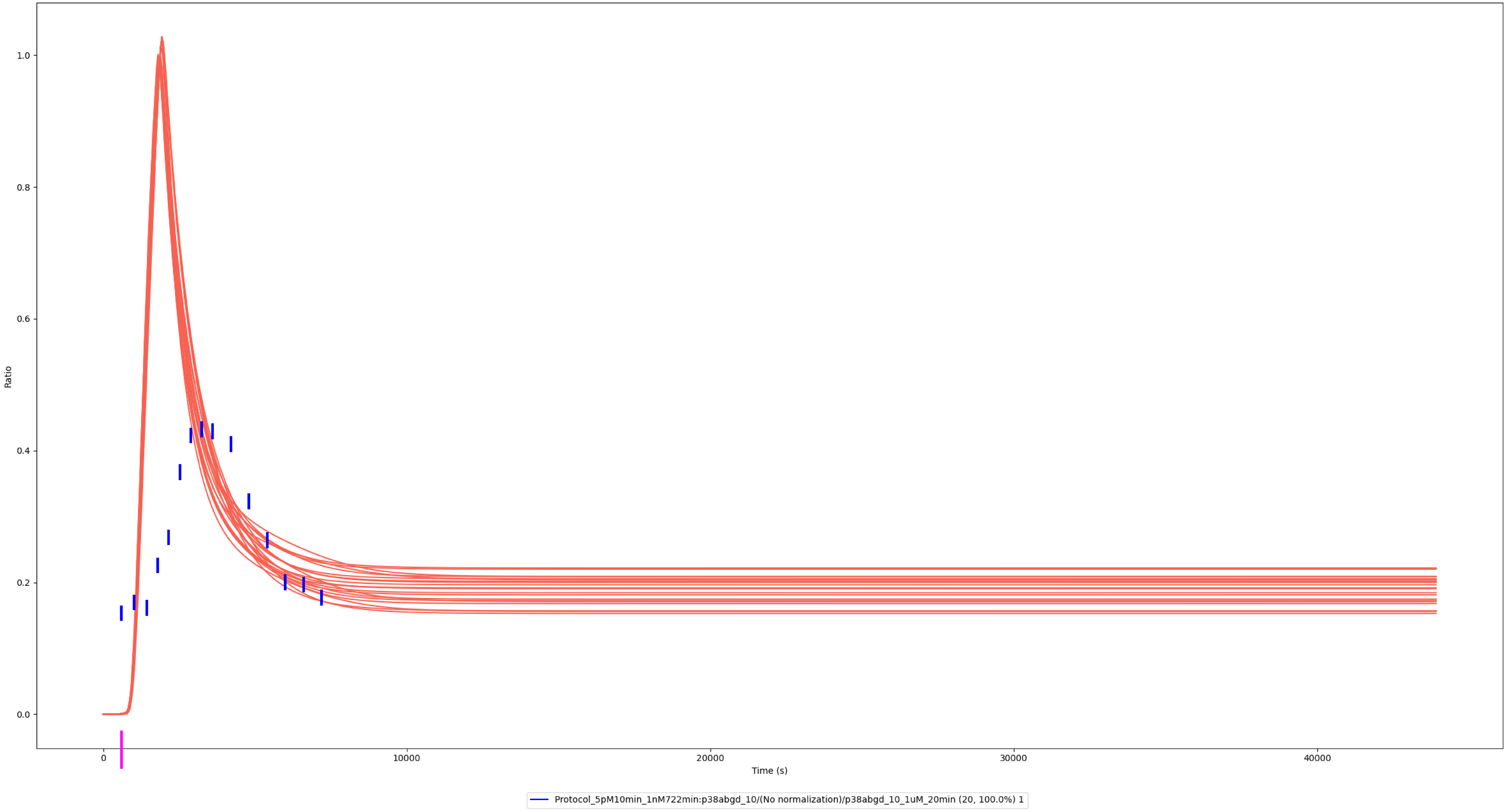

Experiment vs. Simulation: 10 nM LPS, pp-p38α/β/γ/δ

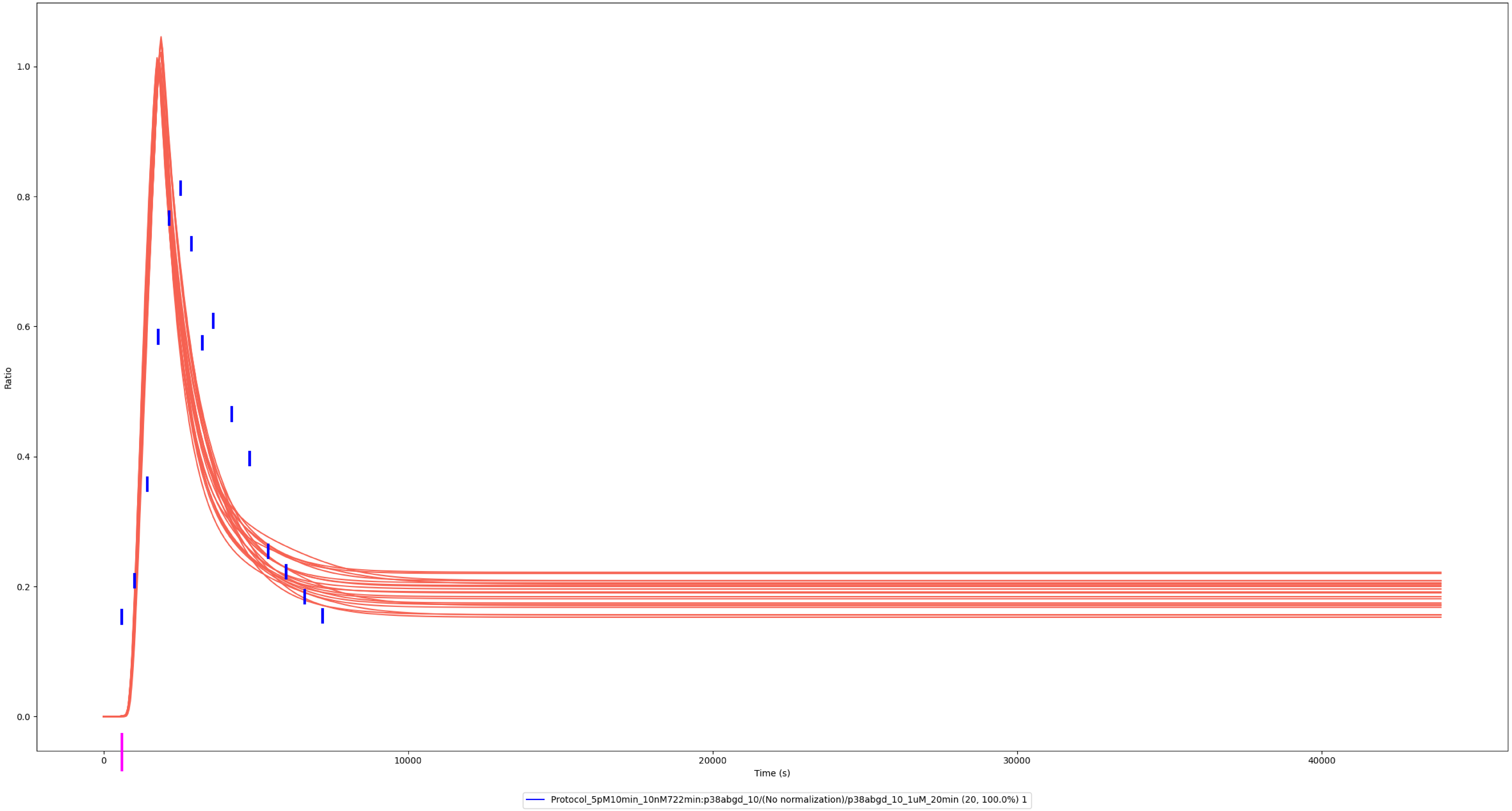

Experiment vs. Simulation: 100 nM LPS, pp-p38α/β/γ/δ

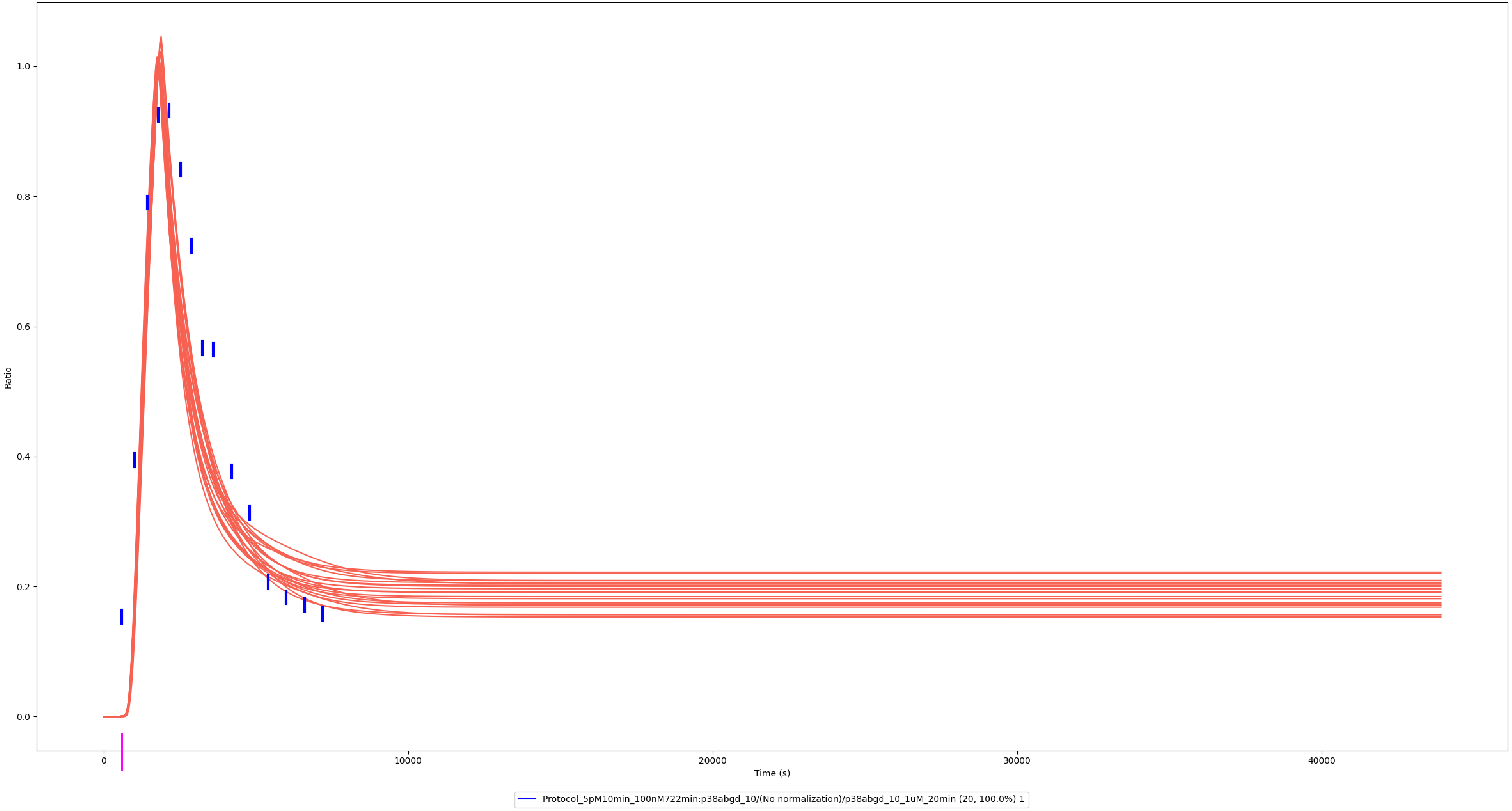

Experiment vs. Simulation: 1,000 nM LPS, pp-p38α/β/γ/δ

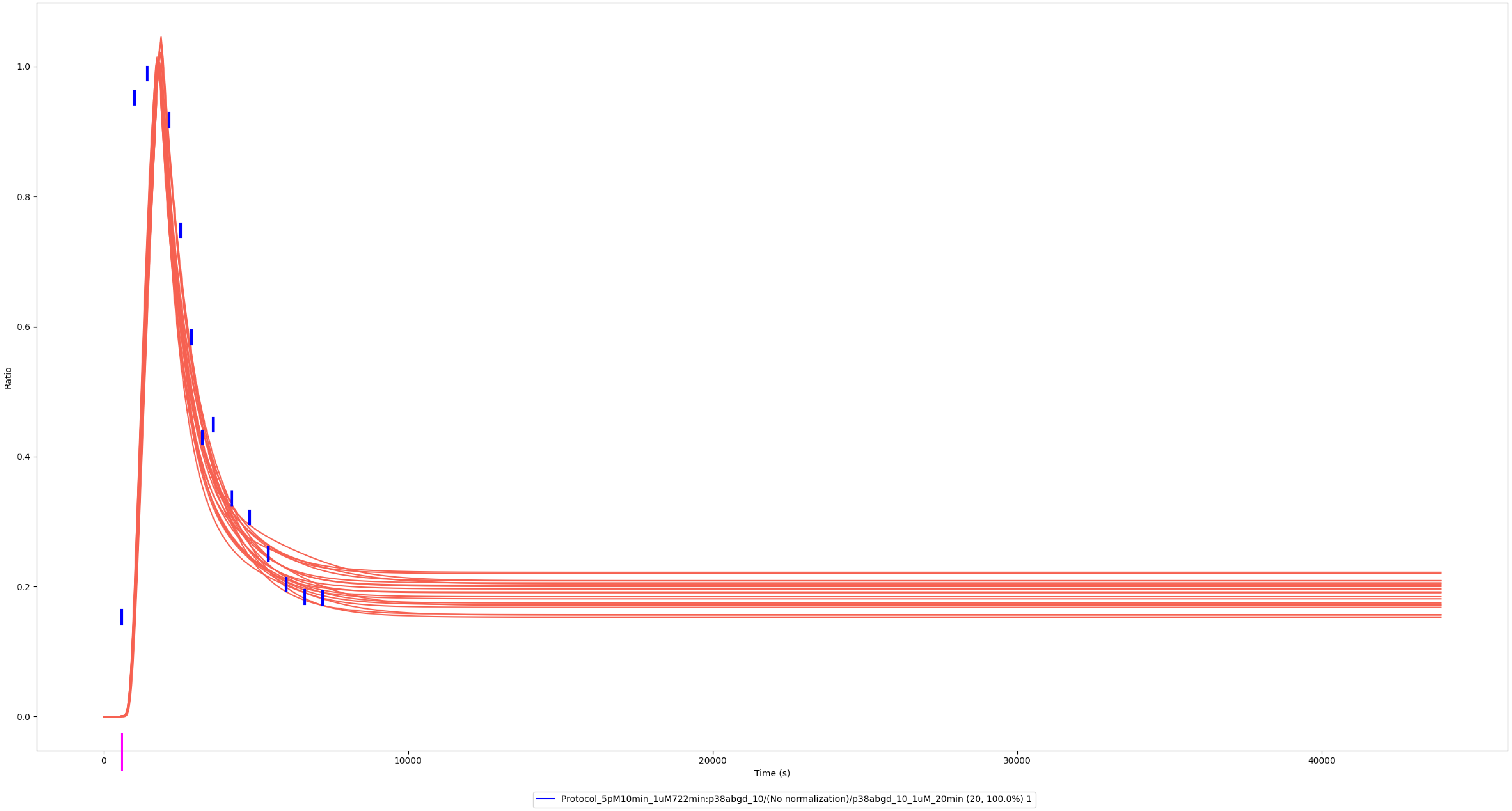

Experiment vs. Simulation: 1 nM LPS, pp-p38α/β/γ/δ

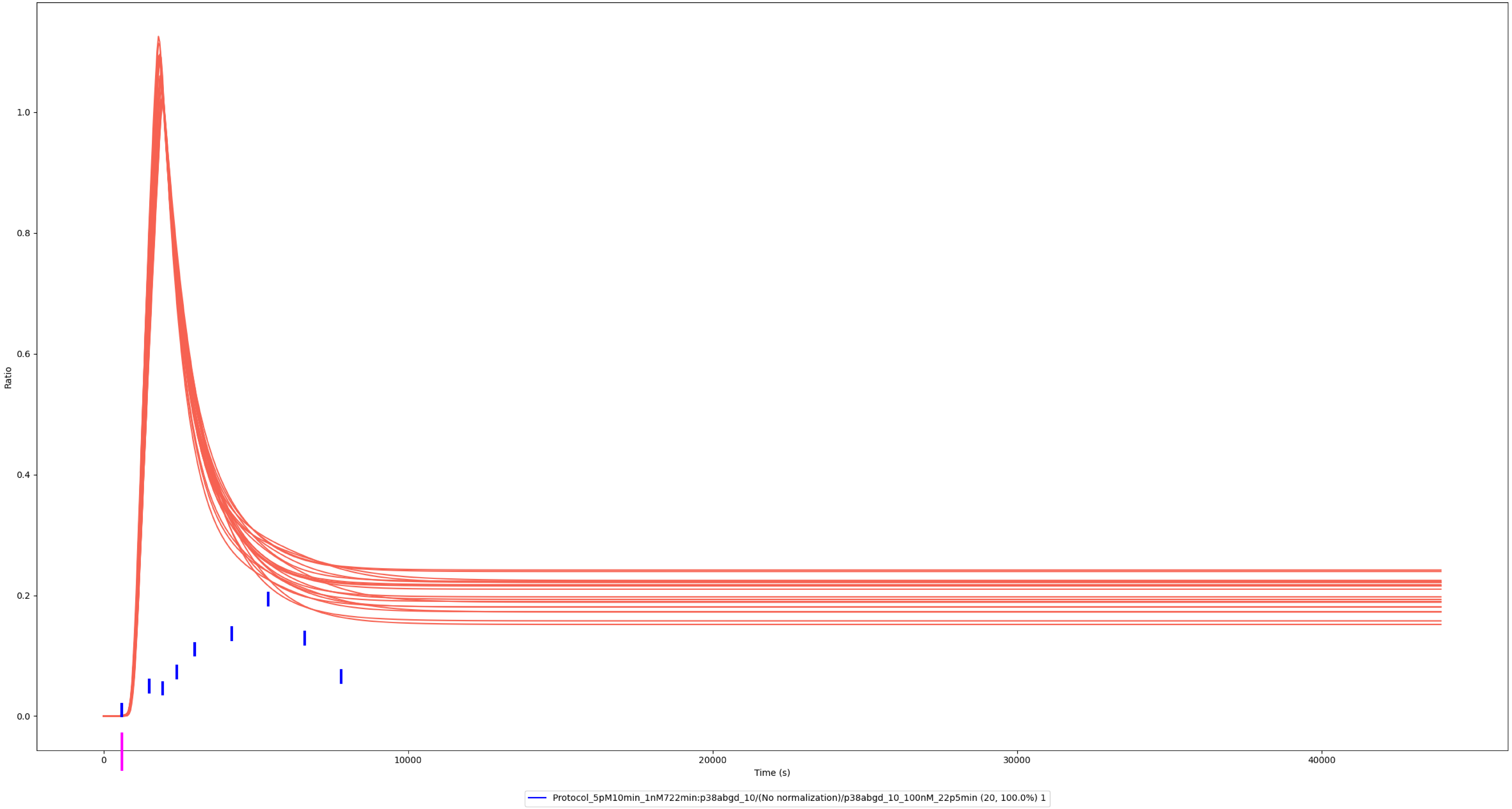

Experiment vs. Simulation: 100 nM LPS, pp-p38α/β/γ/δ

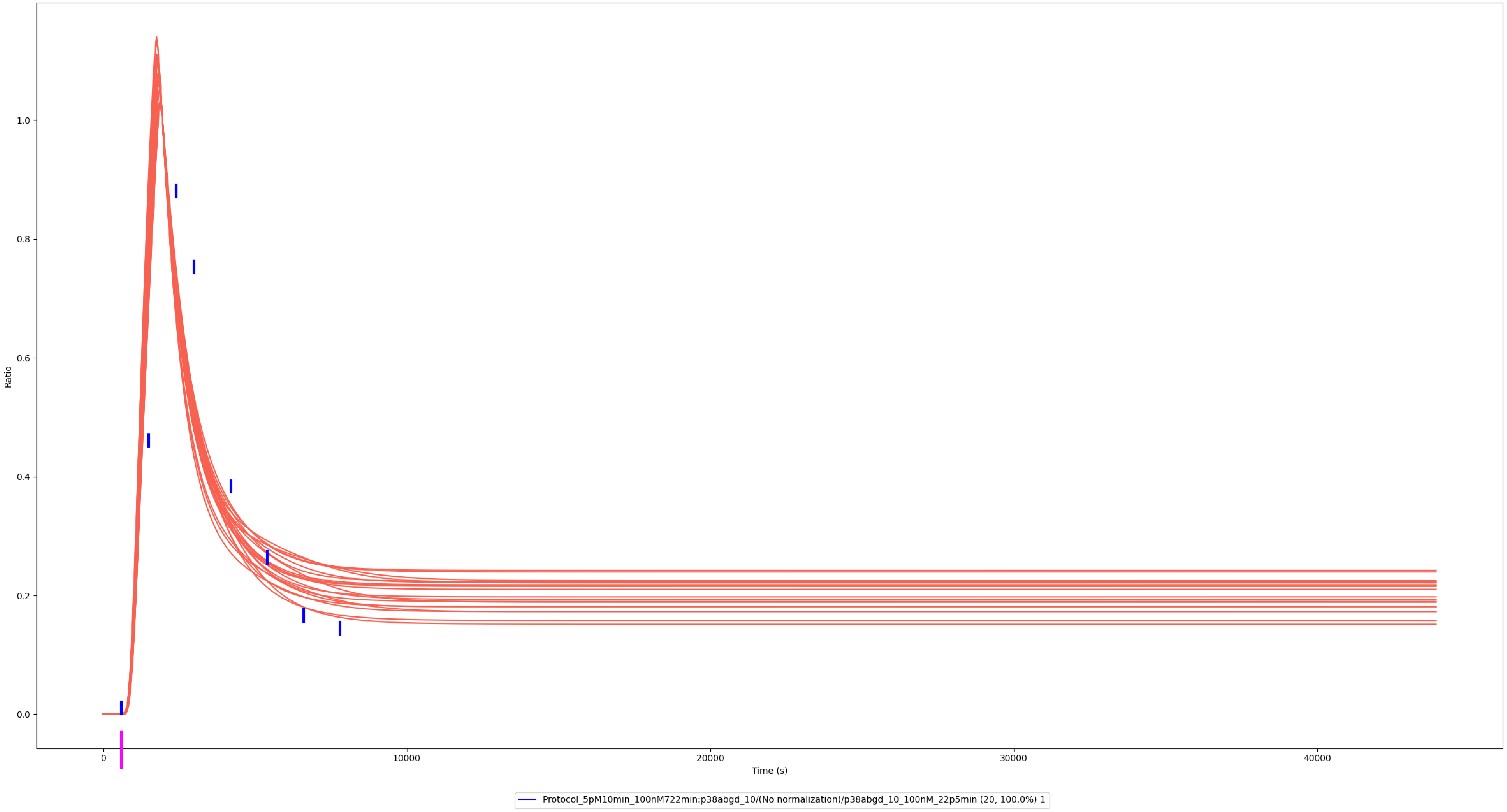

Experiment vs. Simulation: 3 nM LPS, pp-p38α

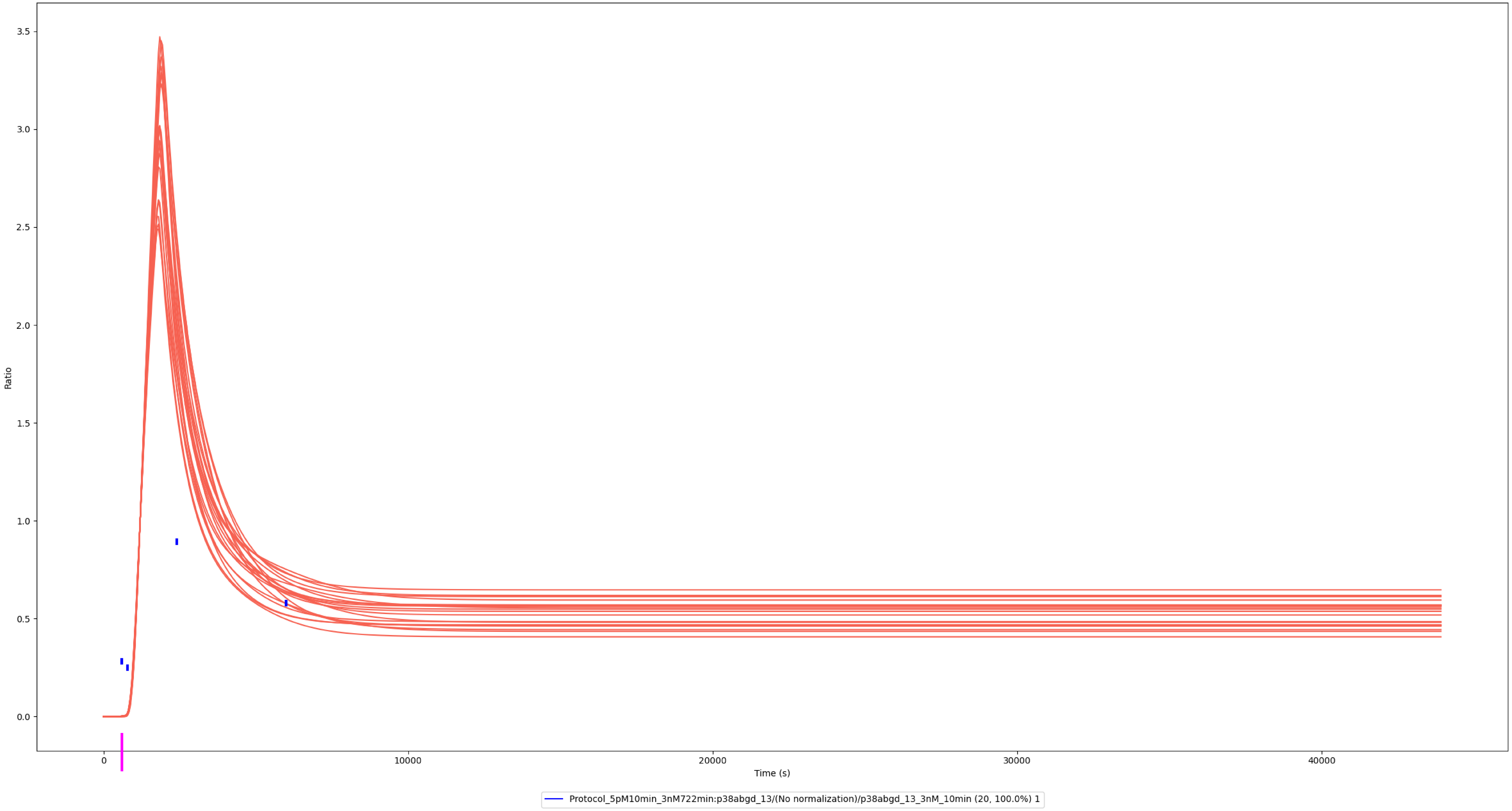

Experiment vs. Simulation: 30 nM LPS, pp-p38α

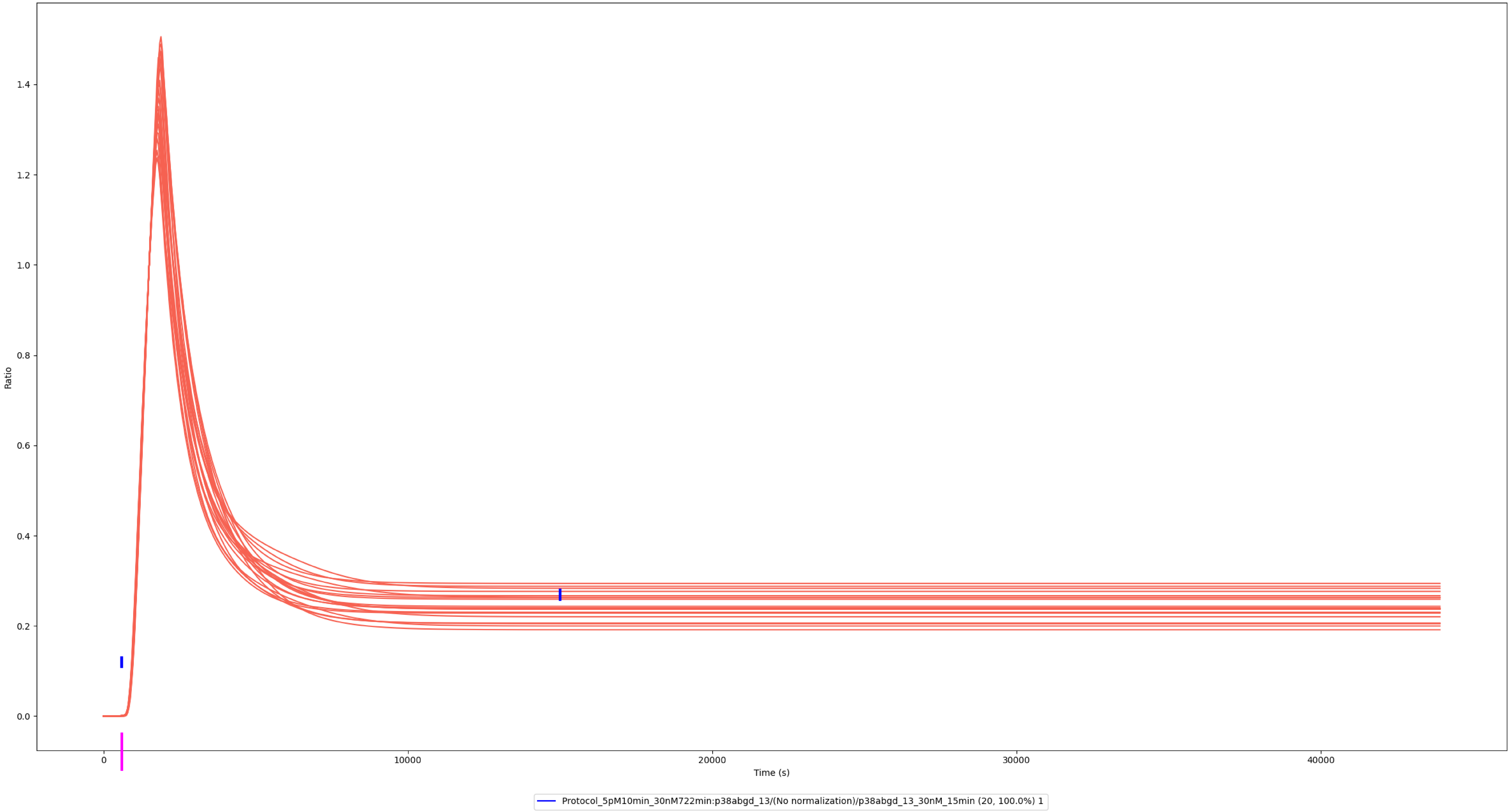

Experiment vs. Simulation: 30 nM LPS, pp-p38α

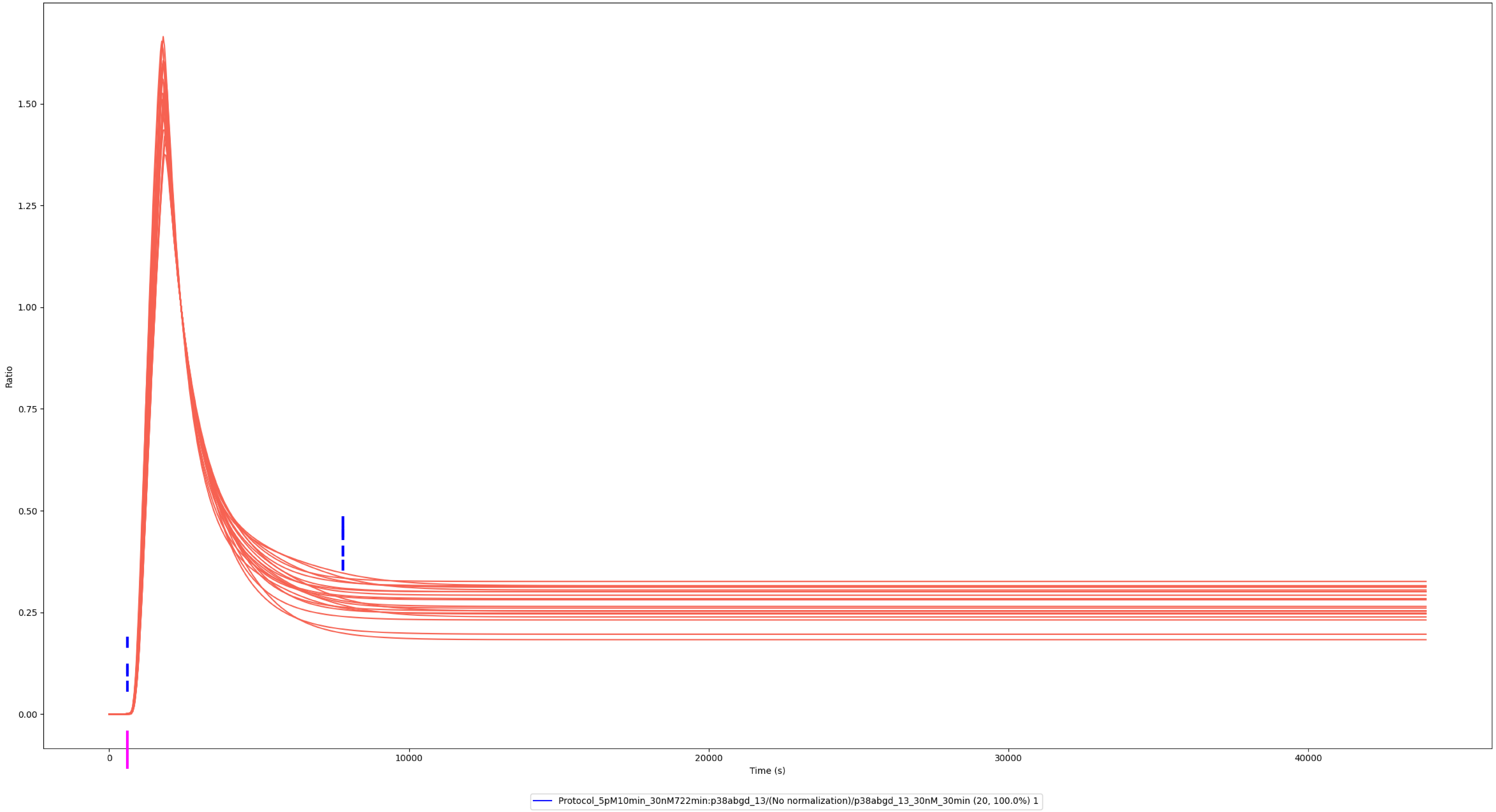

Experiment vs. Simulation: 30 nM LPS, pp-p38β

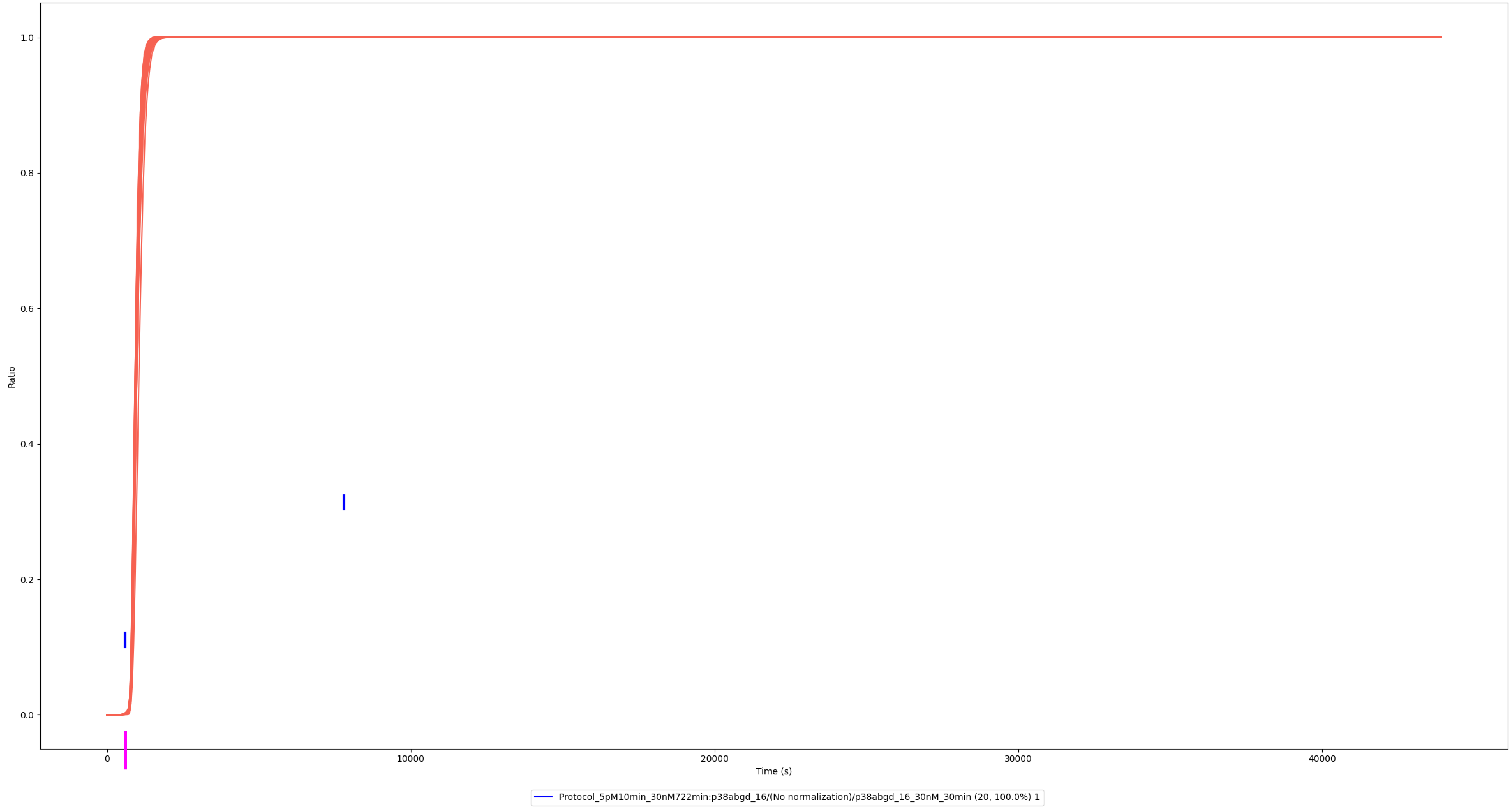

Experiment vs. Simulation: 30 nM LPS, pp-p38β

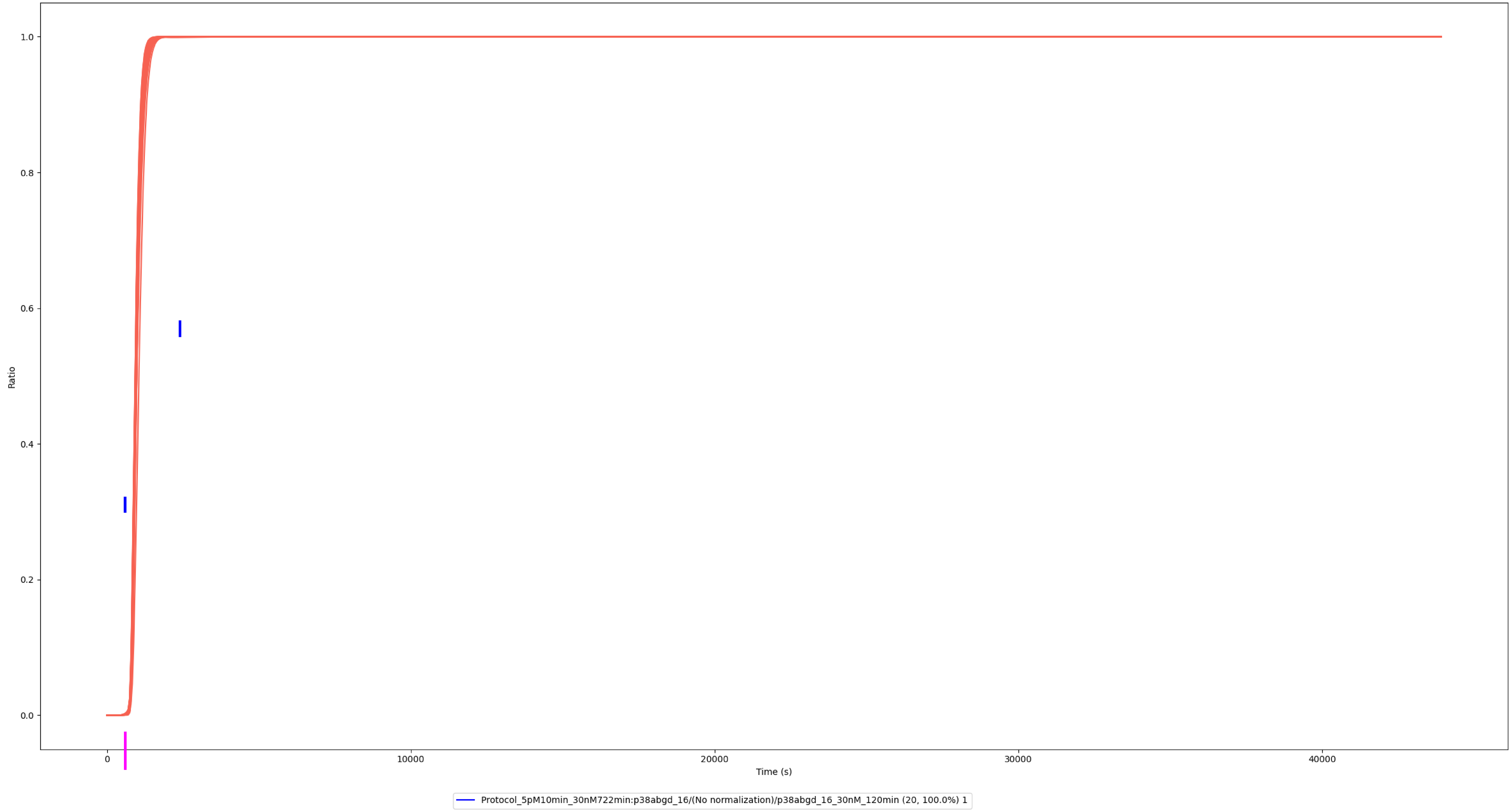

Experiment vs. Simulation: 30 nM LPS, pp-p38γ

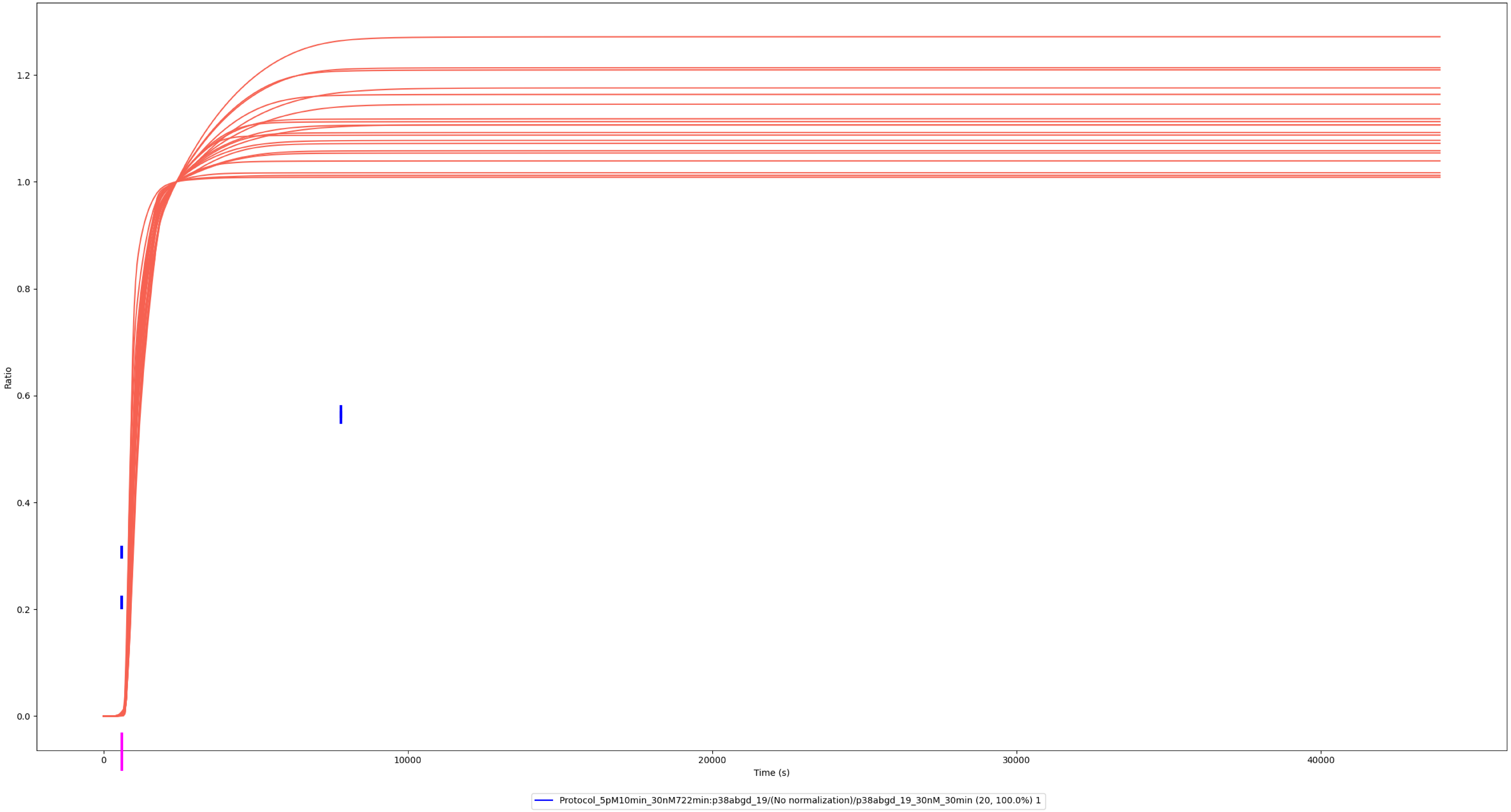

Experiment vs. Simulation: 30 nM LPS, pp-p38γ

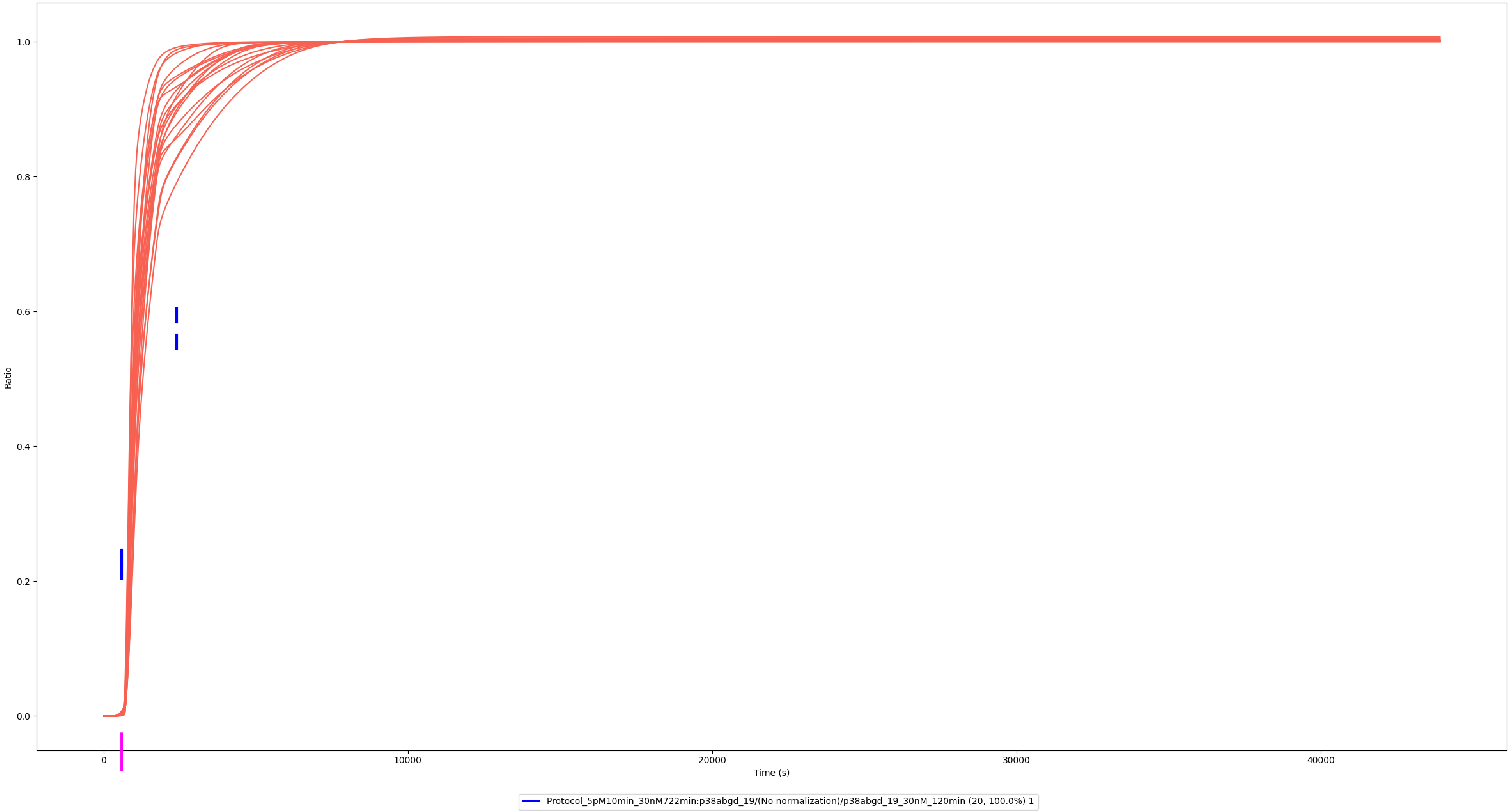

MSK1

Experiment vs. Simulation: 3 nM LPS, pppp-MSK1

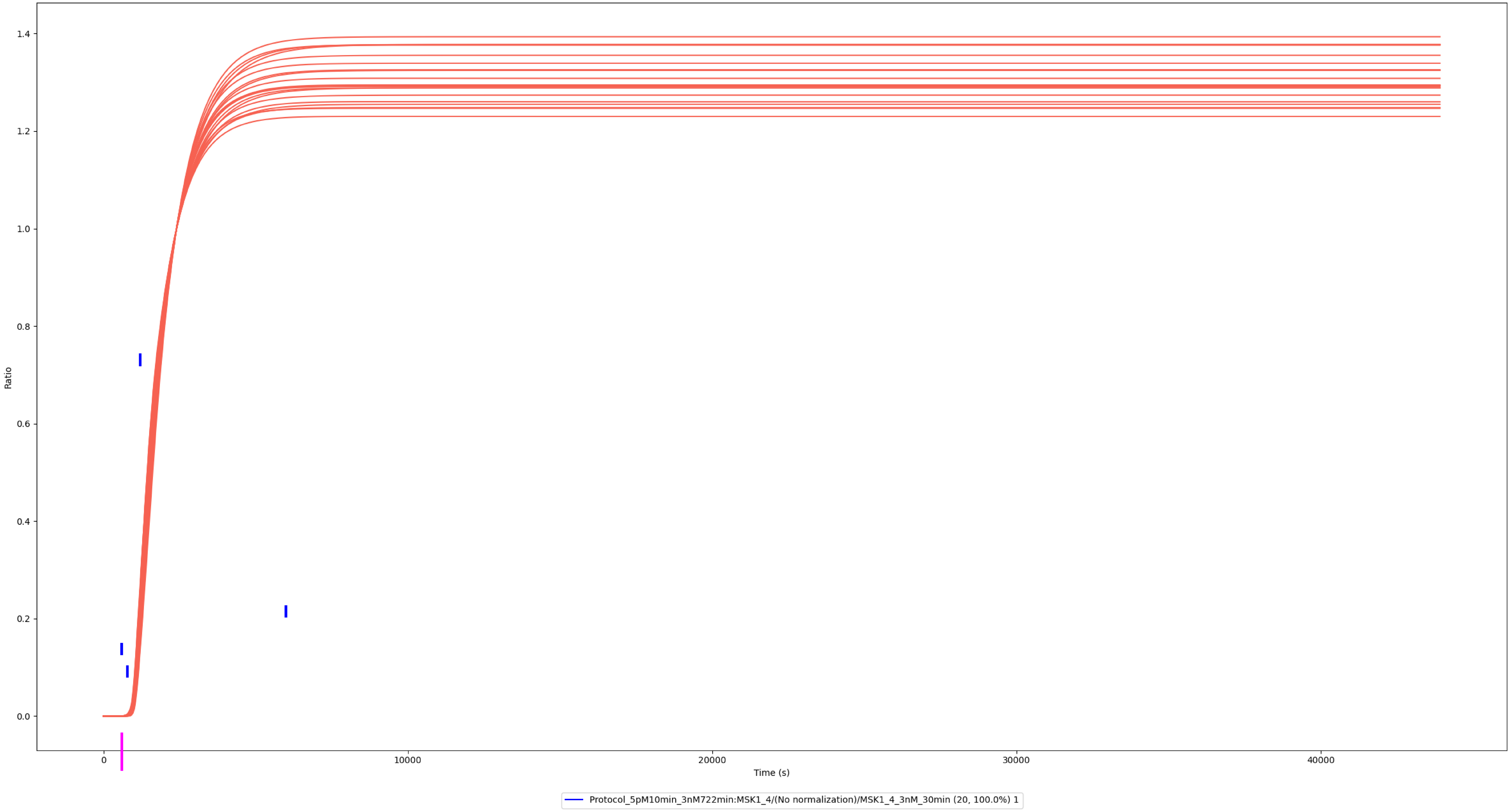

Experiment vs. Simulation: 30 nM LPS, pppp-MSK1

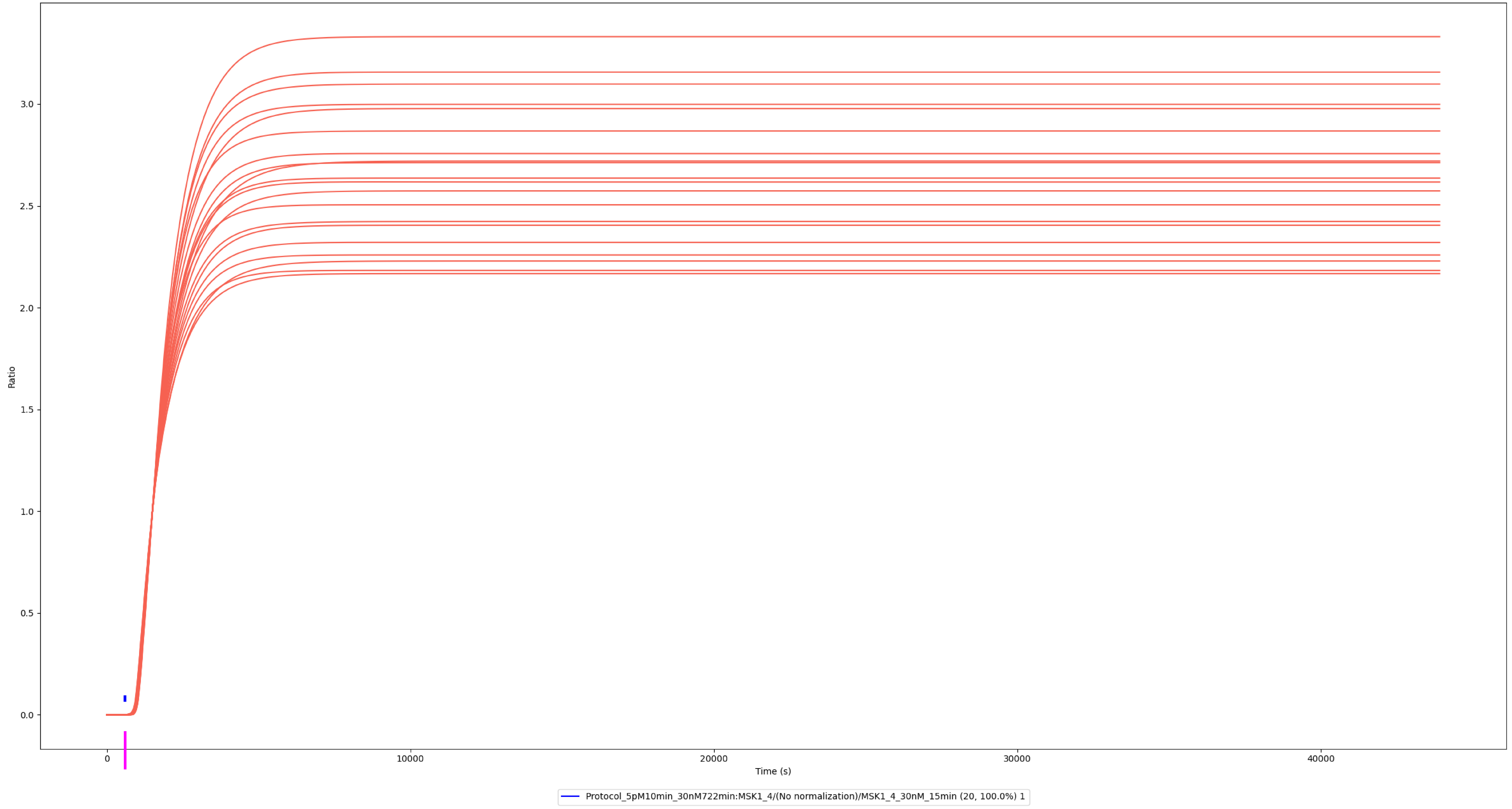

Experiment vs. Simulation: 30 nM LPS, pppp-MSK1

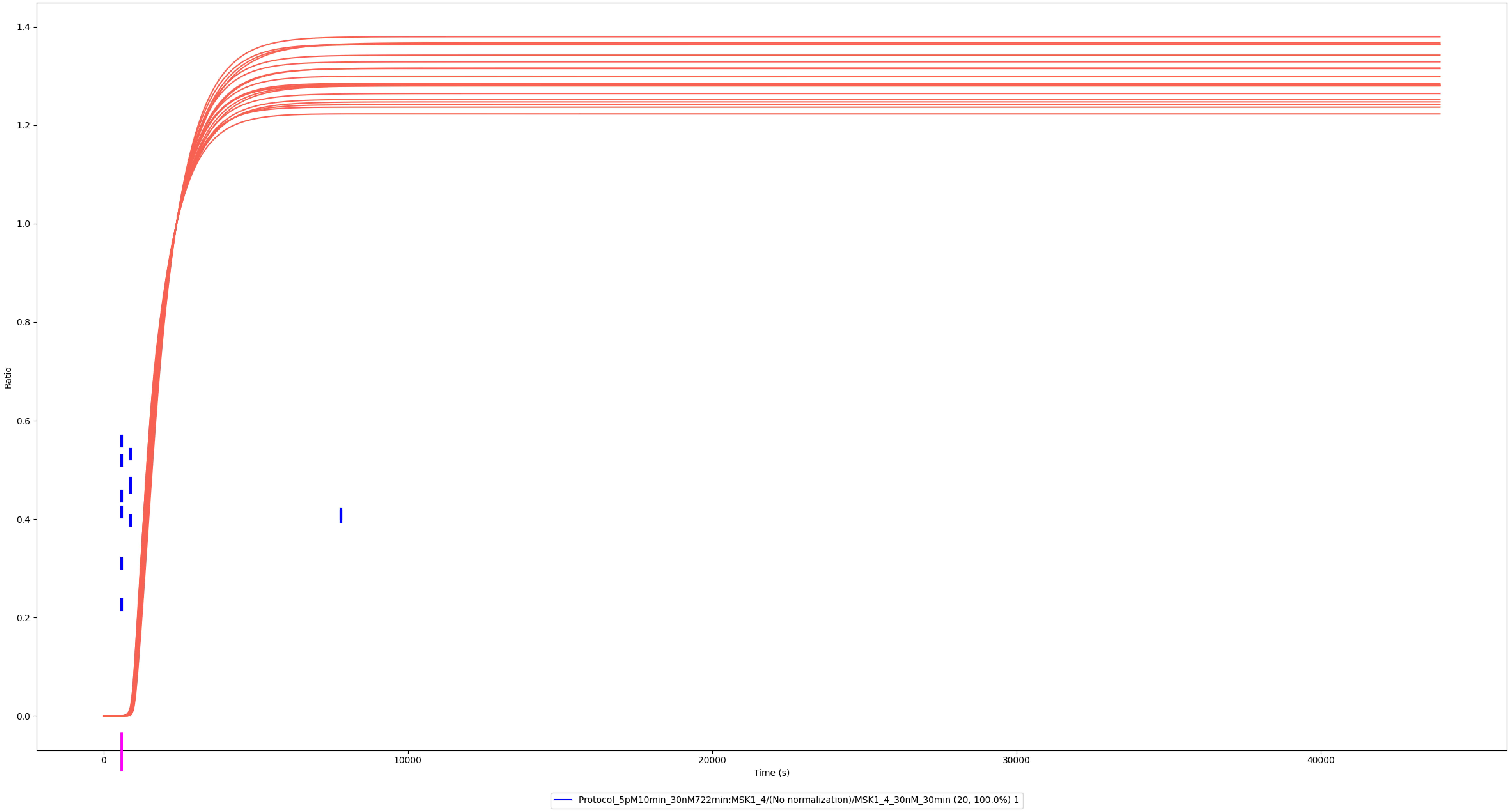

Jun, JunB, JunD

Experiment vs. Simulation: 30 nM LPS, p-Jun

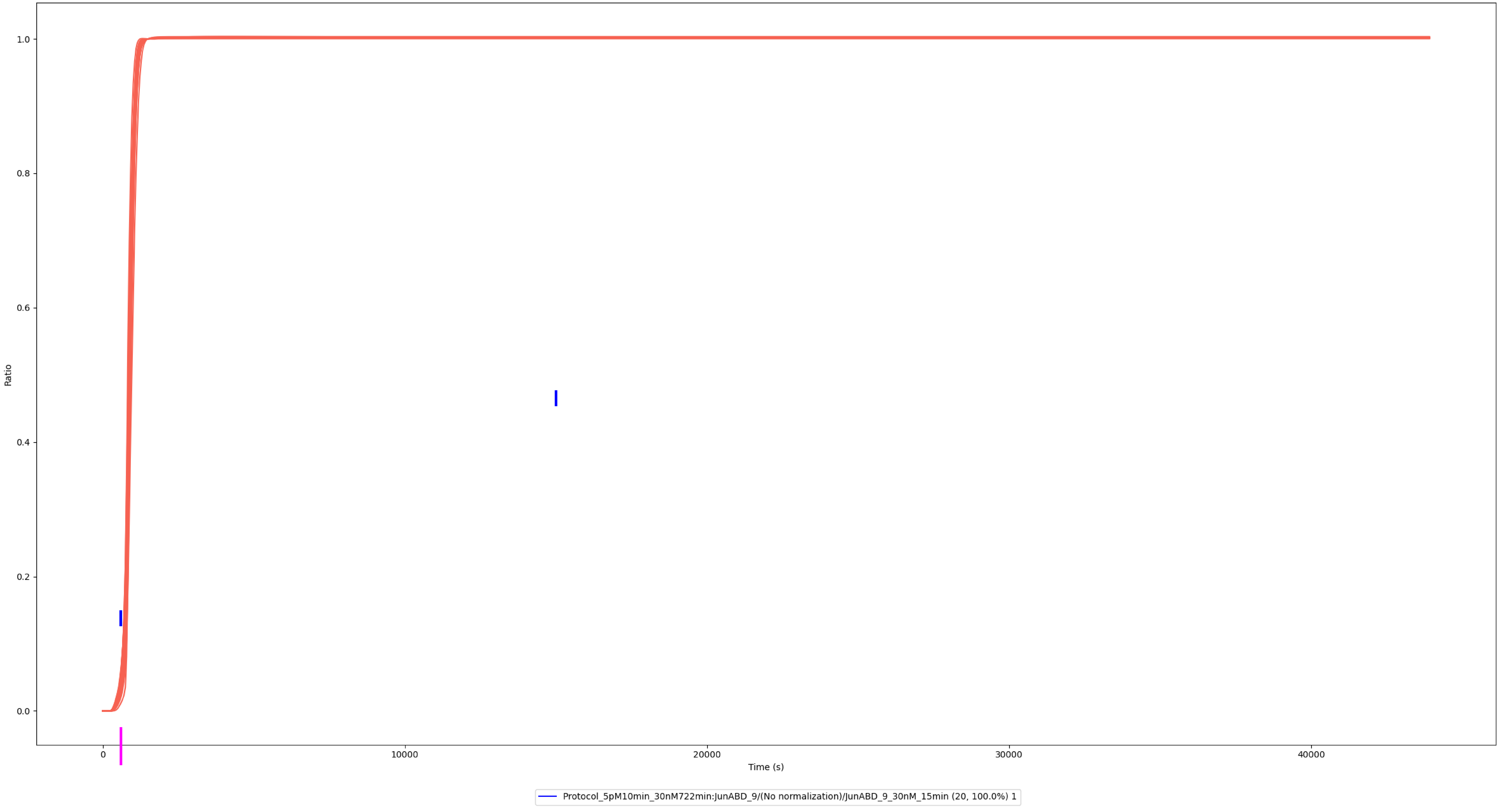

Experiment vs. Simulation: 30 nM LPS, p-Jun

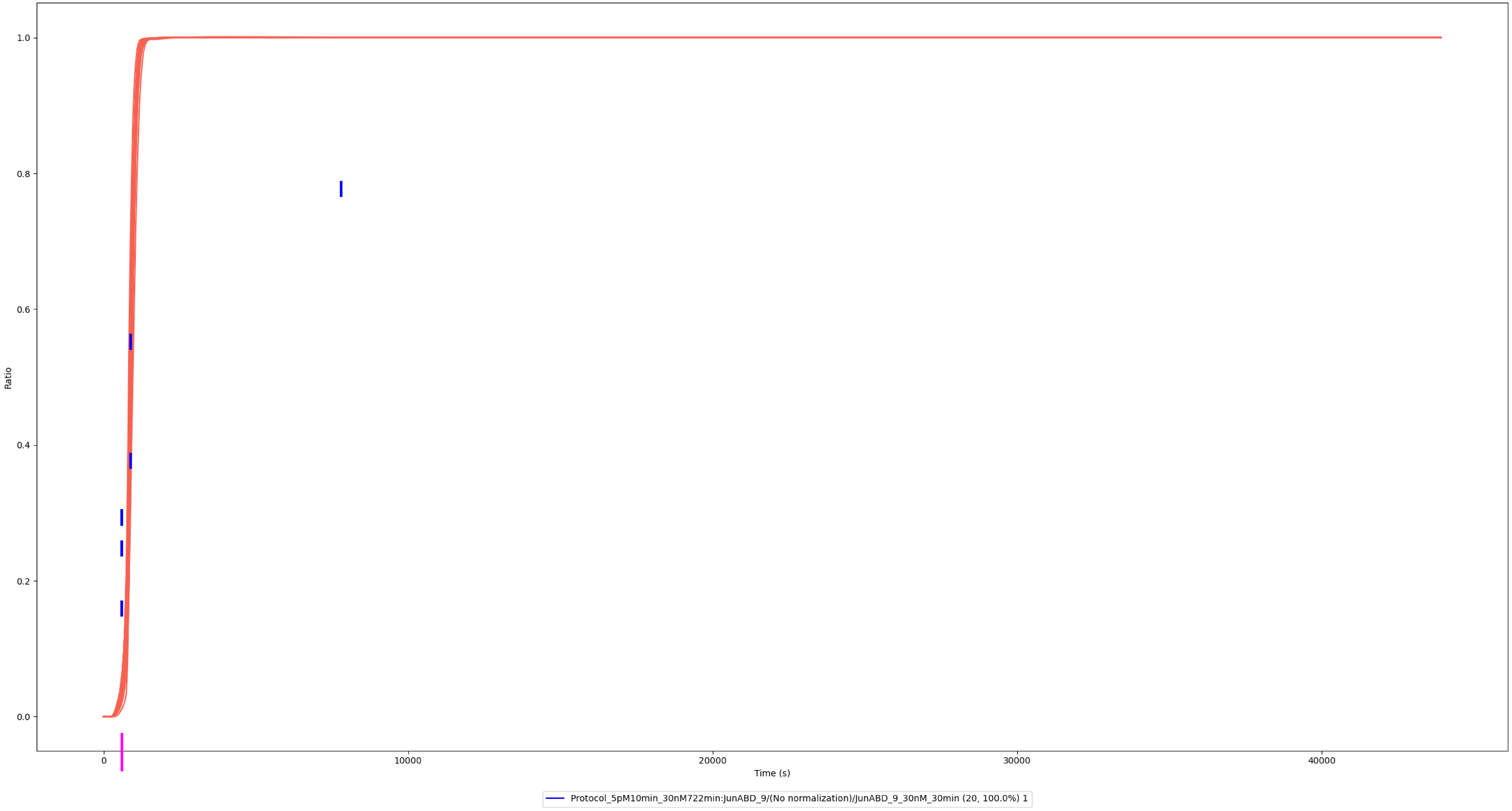

Experiment vs. Simulation: 30 nM LPS, p-Jun

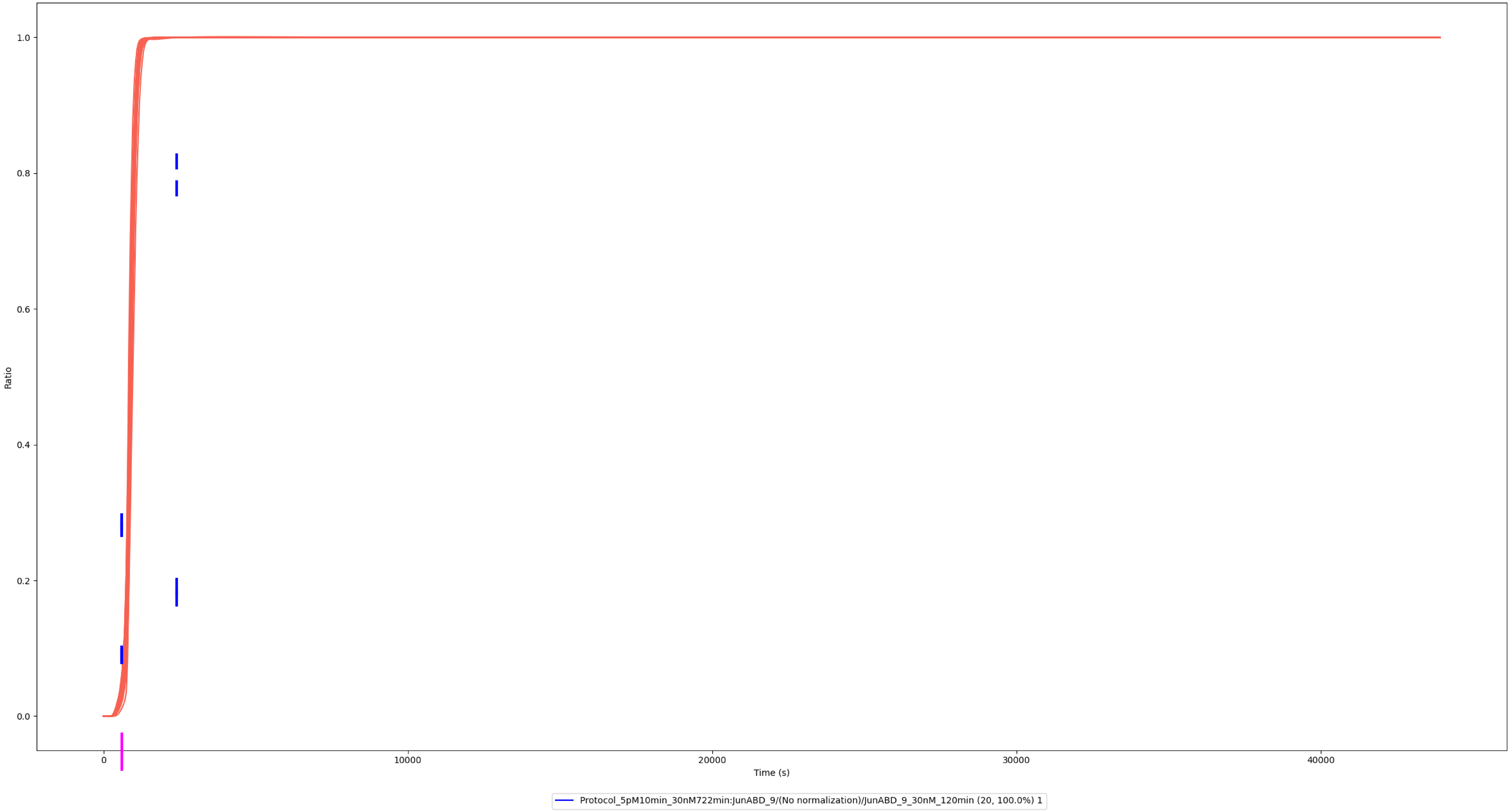

Experiment vs. Simulation: 300 nM LPS, p-Jun

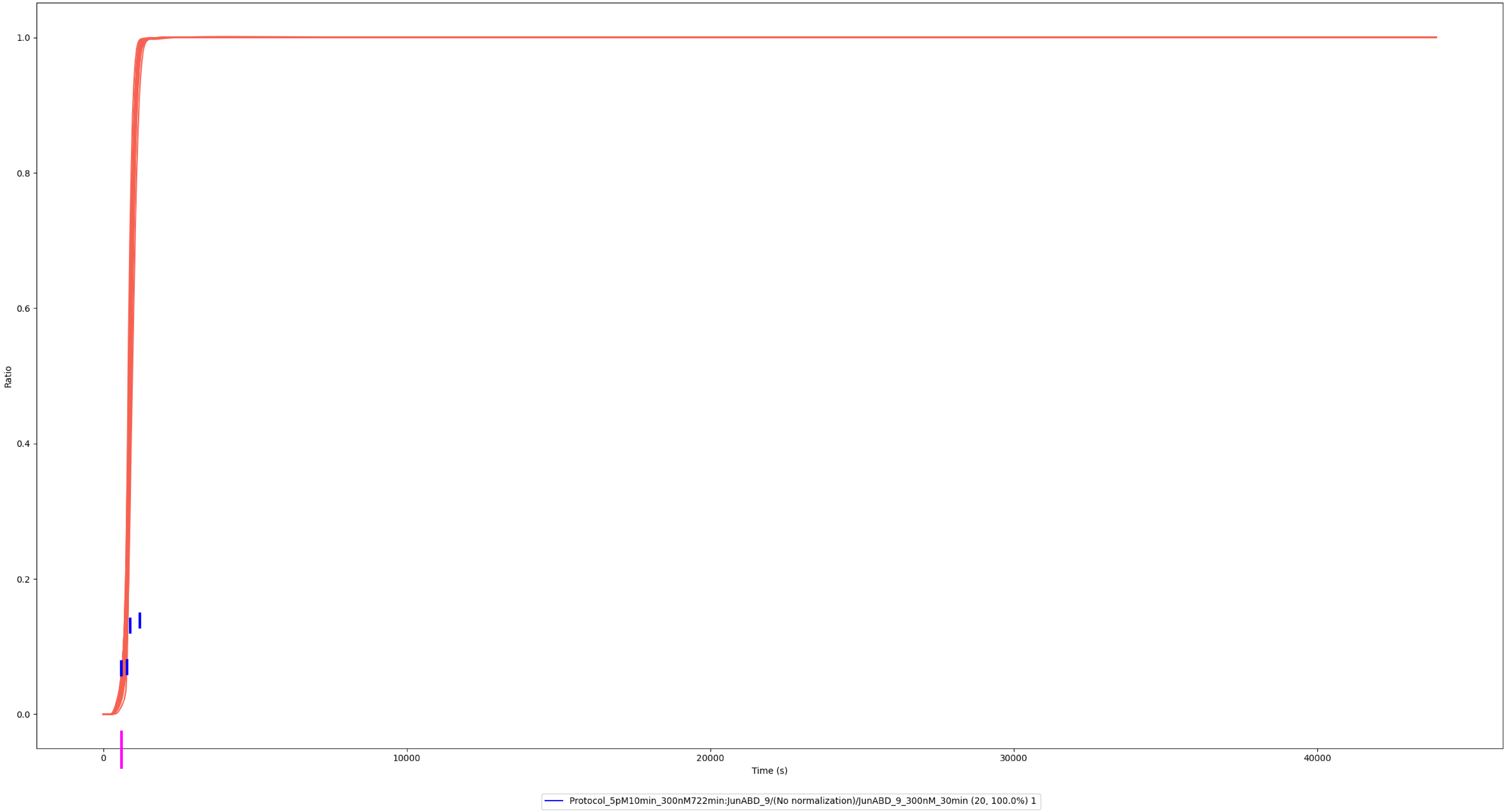

Experiment vs. Simulation: 30 nM LPS, p-JunB

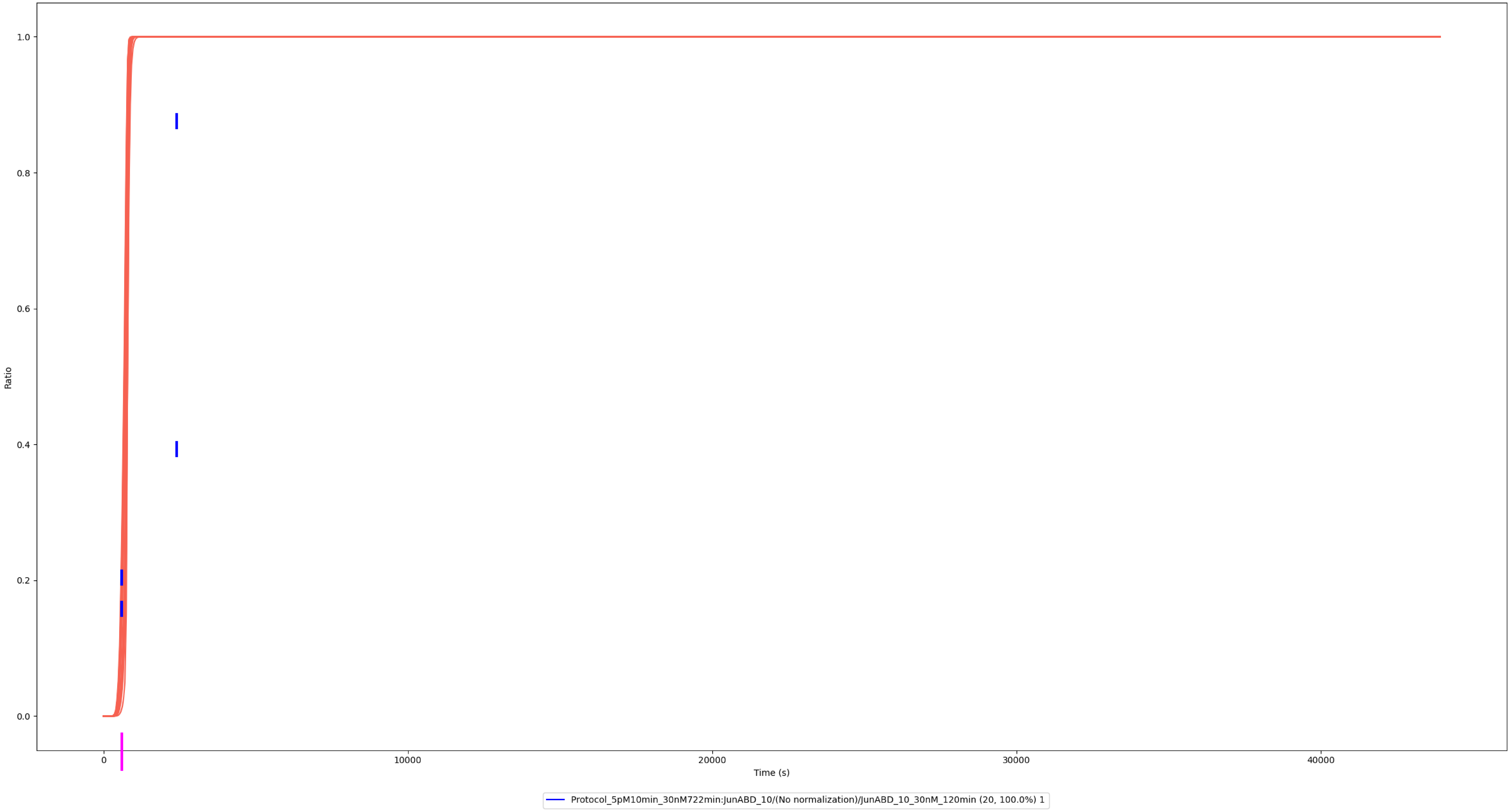

Experiment vs. Simulation: 30 nM LPS, p-JunD

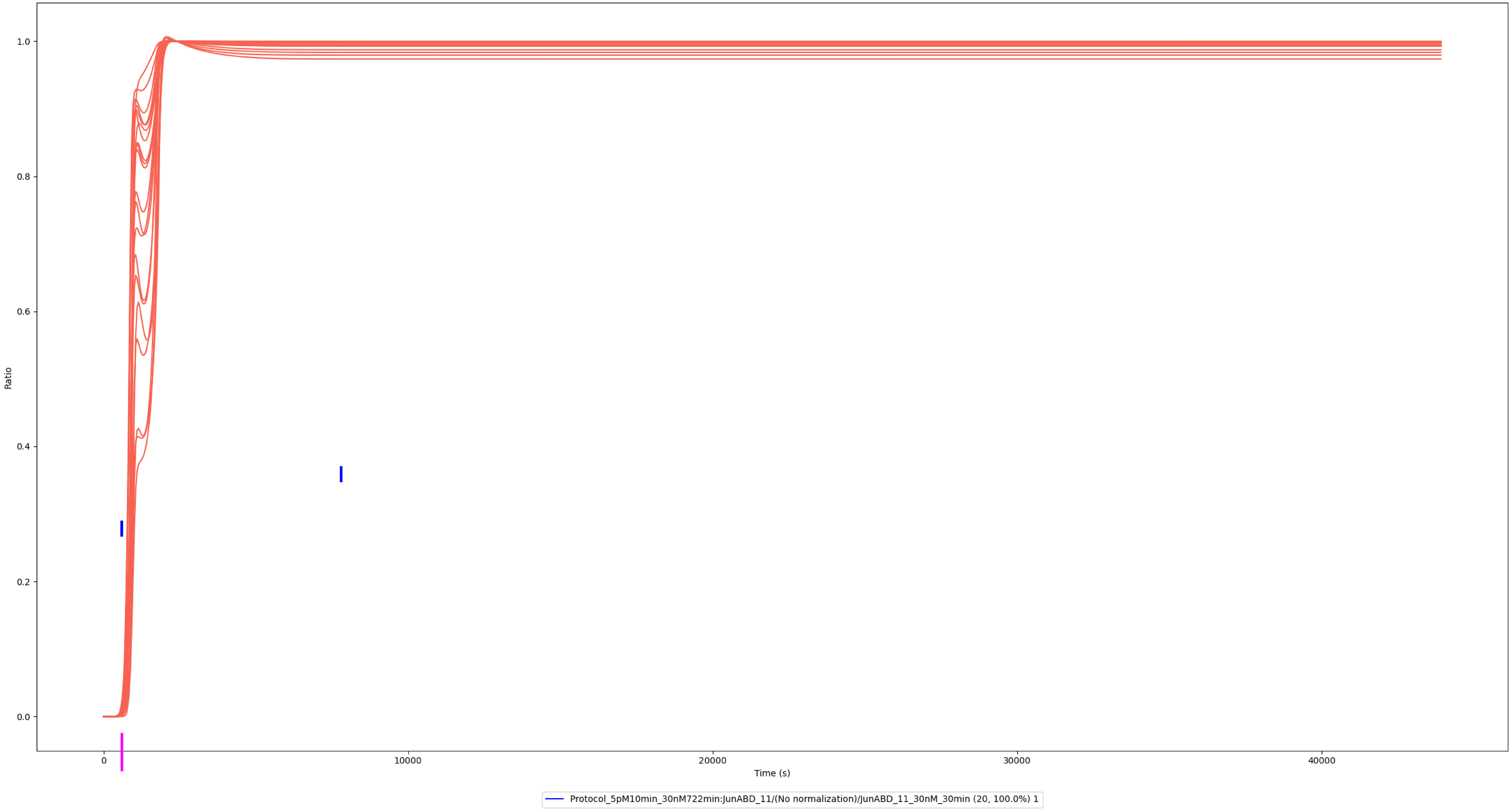

Experiment vs. Simulation: 30 nM LPS, p-Jun/p-JunD

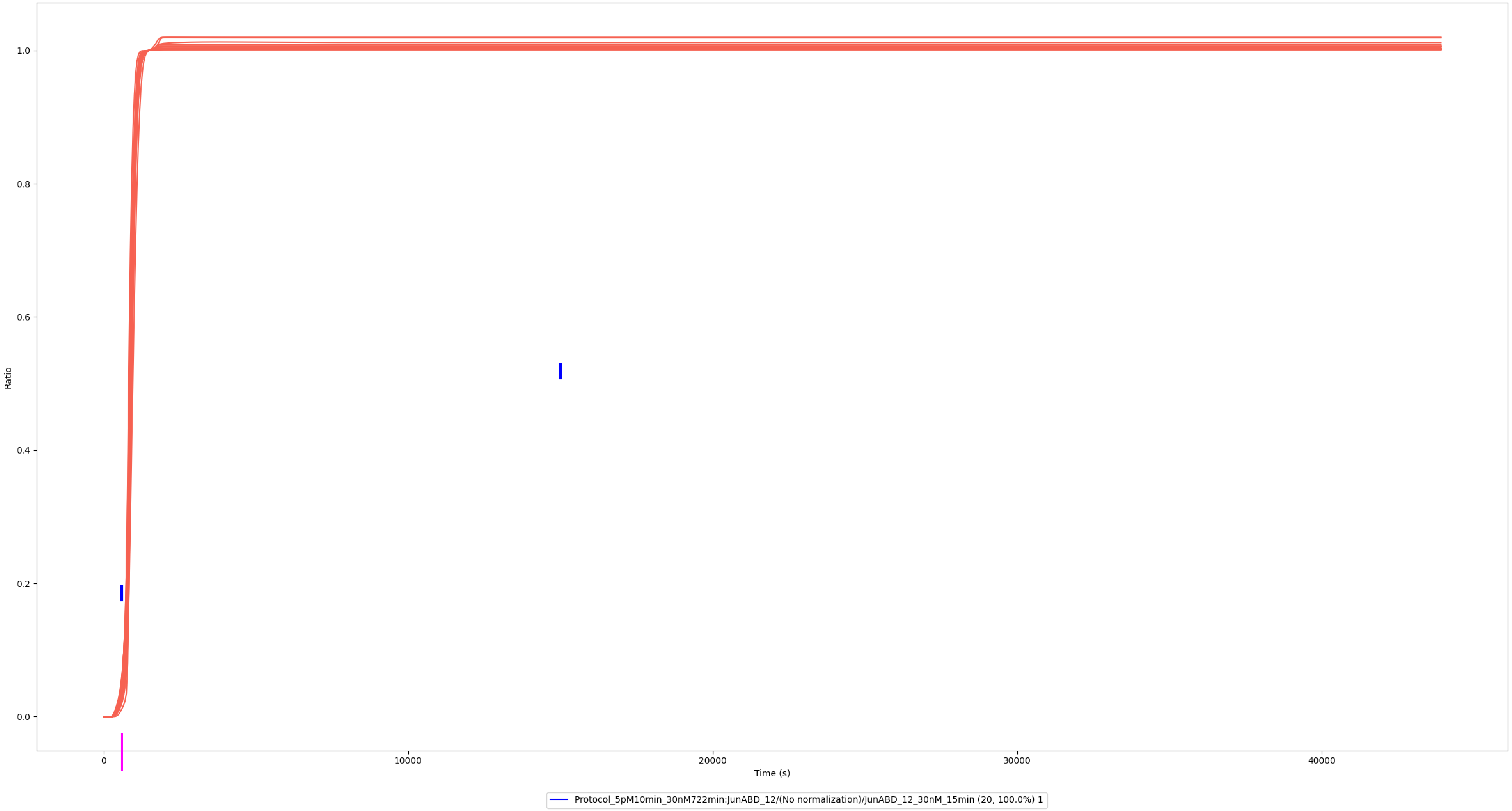

Experiment vs. Simulation: 30 nM LPS, p-Jun/p-JunD

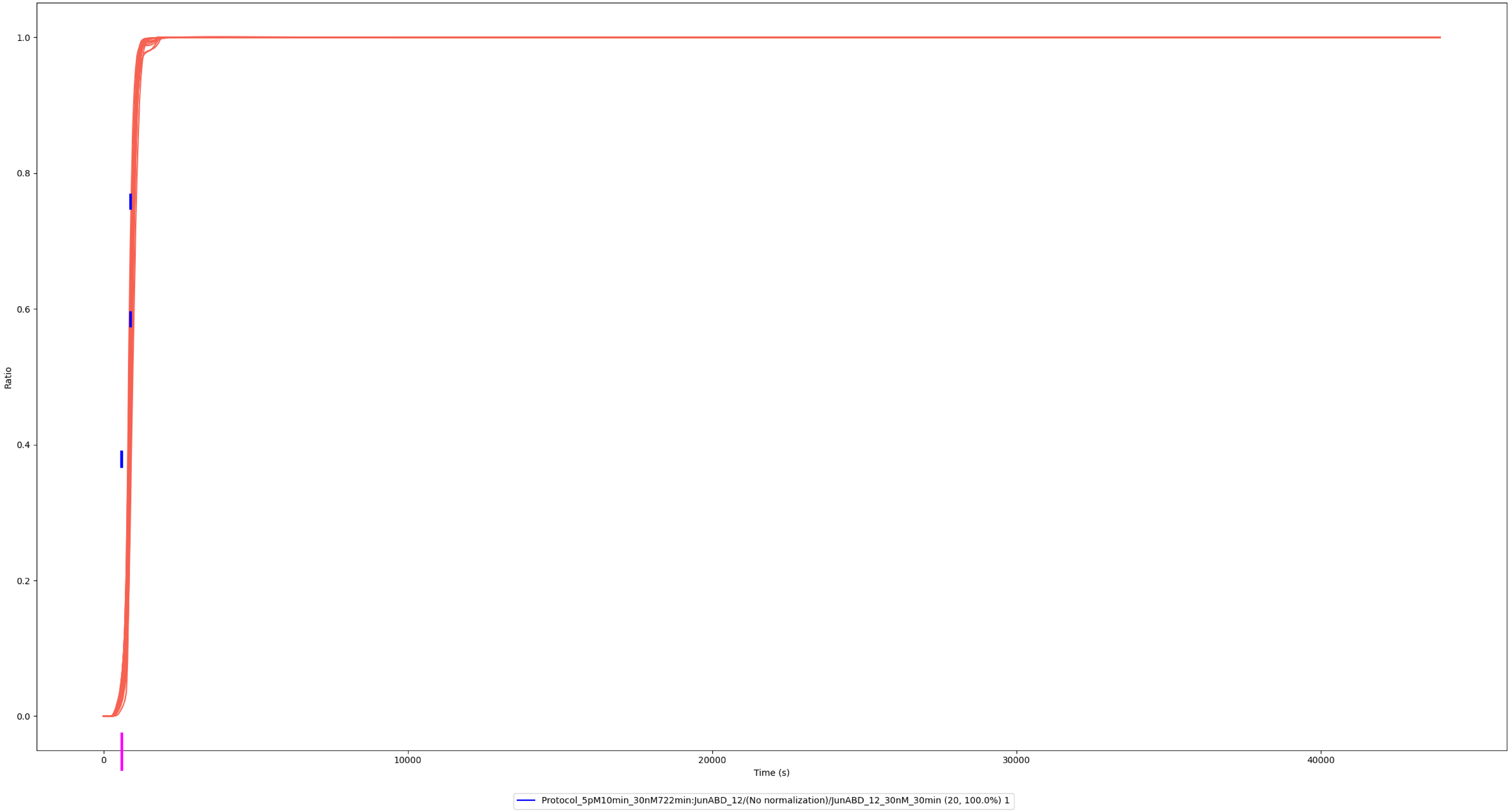

Experiment vs. Simulation: 30 nM LPS, p-Jun/p-JunD

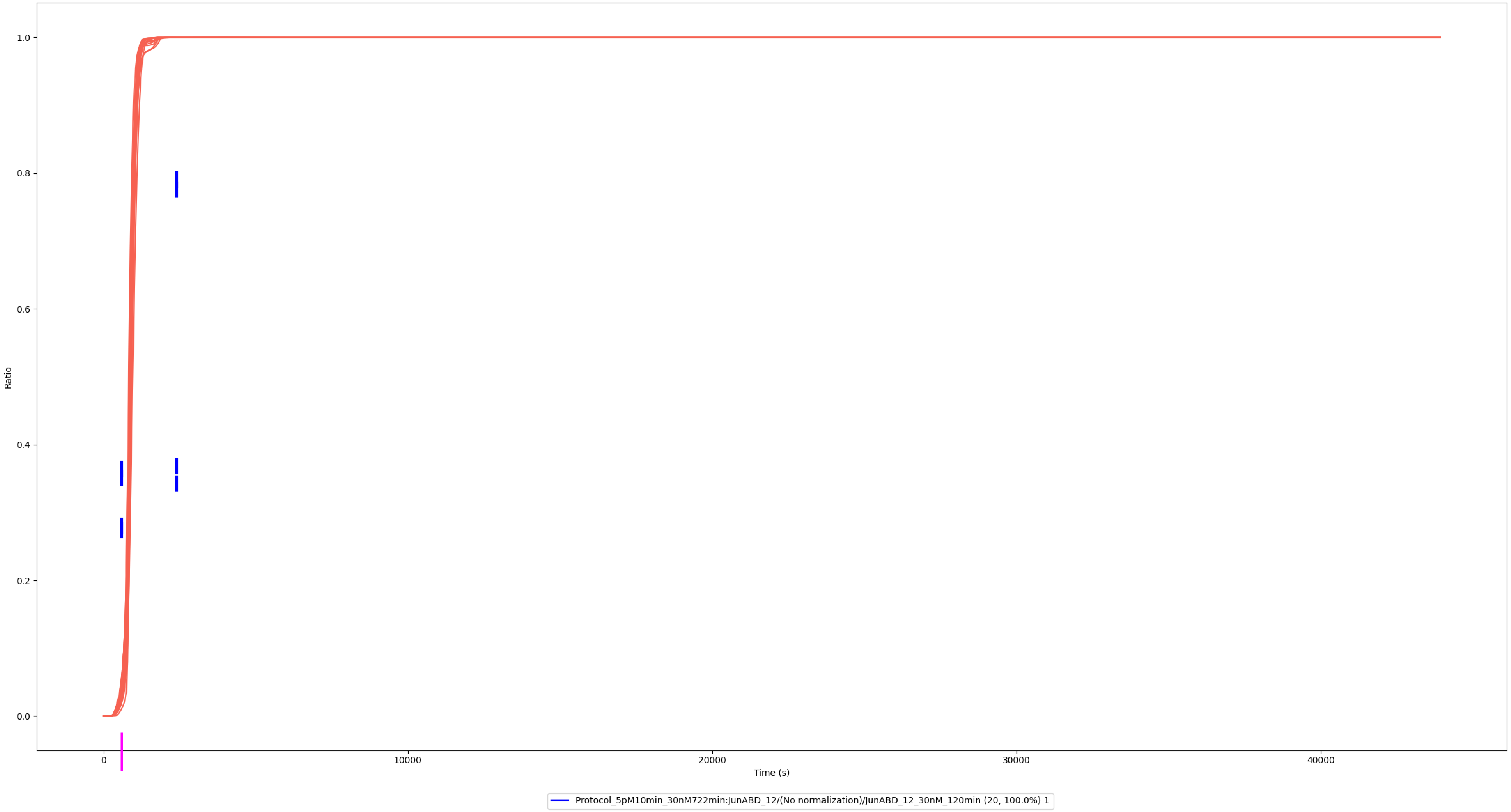

Supplement: Supplementary file 2 [file media-2.zip › DatasetS2.pdf]
